# Tissue macrophages: origin, heterogenity, biological functions, diseases and therapeutic targets

**DOI:** 10.1038/s41392-025-02124-y

**Published:** 2025-03-07

**Authors:** Fan Guan, Ruixuan Wang, Zhenjie Yi, Peng Luo, Wanyao Liu, Yao Xie, Zaoqu Liu, Zhiwei Xia, Hao Zhang, Quan Cheng

**Affiliations:** 1 Department of Neurosurgery, Xiangya Hospital, Central South University, Changsha, China; 2 National Clinical Research Center for Geriatric Disorders, Xiangya Hospital, Central South University, Changsha, China; 3 Xiangya School of Medicine, Central South University, Changsha, China; 4 Department of Oncology, Zhujiang Hospital, Southern Medical University, Guangzhou, China; 5 Institute of Basic Medical Sciences, Chinese Academy of Medical Sciences and Peking Union Medical College, Beijing, China; 6 Department of Neurology, Hunan Aerospace Hospital, Hunan Normal University, Changsha, China; 7 Department of Neurosurgery, The Second Affiliated Hospital, Chongqing Medical University, Chongqing, China

**Keywords:** Prognostic markers, Senescence, Diseases

## Abstract

Macrophages are immune cells belonging to the mononuclear phagocyte system. They play crucial roles in immune defense, surveillance, and homeostasis. This review systematically discusses the types of hematopoietic progenitors that give rise to macrophages, including primitive hematopoietic progenitors, erythro-myeloid progenitors, and hematopoietic stem cells. These progenitors have distinct genetic backgrounds and developmental processes. Accordingly, macrophages exhibit complex and diverse functions in the body, including phagocytosis and clearance of cellular debris, antigen presentation, and immune response, regulation of inflammation and cytokine production, tissue remodeling and repair, and multi-level regulatory signaling pathways/crosstalk involved in homeostasis and physiology. Besides, tumor-associated macrophages are a key component of the TME, exhibiting both anti-tumor and pro-tumor properties. Furthermore, the functional status of macrophages is closely linked to the development of various diseases, including cancer, autoimmune disorders, cardiovascular disease, neurodegenerative diseases, metabolic conditions, and trauma. Targeting macrophages has emerged as a promising therapeutic strategy in these contexts. Clinical trials of macrophage-based targeted drugs, macrophage-based immunotherapies, and nanoparticle-based therapy were comprehensively summarized. Potential challenges and future directions in targeting macrophages have also been discussed. Overall, our review highlights the significance of this versatile immune cell in human health and disease, which is expected to inform future research and clinical practice.

## Introduction

Macrophages are immune cells widely distributed in the blood and tissue of the body. They, along with peripheral blood mononuclear cells and dendritic cells (DCs), belong to the mononuclear phagocyte system (MPS), playing important roles in immune defense, surveillance, and homeostasis.^1,2^ At least three types of hematopoietic progenitors: primitive hematopoietic progenitors, erythro-myeloid progenitors (EMPs), and hematopoietic stem cells (HSCs) exist in vertebrates, which have distinct genetic backgrounds and developmental process.^3,4^ Primitive hematopoietic progenitors are of RUNX1-independent origin, and have been reported to produce macrophages in zebrafish and mice; however, macrophages fail to develop at the stage of RUNX1 absence in humans and mice.^5–10^ Thus, whether macrophages could originate from primitive hematopoietic progenitors is uncertain.^3^ Existing research suggests that tissue-resident cells mainly originate from the yolk sac EMPs and EMP-derived macrophage precursors (PreMacs) in the embryonic stage, and they migrate and colonize in specific tissue sites for further differentiation and maturation. They could self-renew locally and persist in the tissue with or without the complement of HSCs-derived monocytes.^11,12^ Noticeably, the identity of macrophage populations is imprinted by their resident tissue, and tissue-specific transcriptional programs are essential for the maintenance, phenotype, and function features of tissue macrophages.^12–17^

Macrophages play complex and diverse roles in almost all aspects of biological processes in the body.^18^ As an important component of innate immunity, macrophages could migrate into damaged sites induced by chemokines, such as CCL2 and CX3CL1, during infection, inflammation, and tissue damage. They ingest diverse pathogens, apoptotic and dead cells, and tissue fragments by phagocytosis and further digest them in phagolysosomes.^3,19–21^ In addition, they could capture and endocytose antigens and further present them to other immune cells, such as T and B cells, thus involved in initiating the adaptive immune responses.^1,22,23^ In addition, macrophages could secrete various active substances (cytokines, chemokines, complements, enzymes, etc.) to regulate inflammation and immune responses.^1,24–26^ Besides, macrophages could sense various physiological and pathological signals and then participate in tissue repair and remodeling as well as metabolic regulation by removing necrotic cells and cell debris, regulating inflammation, and providing nutrition support for the proliferation and repair of cells, etc.^27–30^

The tumor microenvironment (TME) is a complex environment composed of cellular components such as tumor cells, multiple immune cells, endothelial cells, and non-cellular components such as extracellular matrix.^31,32^ Tumor-associated macrophages (TAMs) is a key component of TME, accounting for about 50% of hematopoietic cells.^33^ TAMs have both anti-tumor and pro-tumor properties. TAMs could promote tumor progression by promoting tumor cell proliferation and invasion, increasing the activity of tumor stem cells, inducing angiogenesis, and inhibiting the activity of cytotoxic T cells and natural killer (NK) cells,^34^ while they could also play an anti-tumor role by the phagocytosis and secretion of pro-inflammatory cytokines for the activation of adaptive immune cells.^35^ Furthermore, the functional status of macrophages is closely related to the development of various diseases, such as rheumatoid arthritis (RA), atherosclerosis (AS), Alzheimer’s disease (AD), diabetes, obesity, trauma, etc.^36–41^ With the development of diverse technologies (gene editing, nano-drug delivery system, etc.) and immunotherapies, targeting macrophages to treat diseases has become a promising therapeutic strategy.^42–44^

In this review, we summarize the research history, origin heterogeneity, and polarization of tissue macrophages. We also discuss their biological functions and roles in cancerous and non-cancerous diseases. Correspondingly, we illustrate multiple therapeutic strategies for targeting tissue macrophages.

## Origins and heterogeneity of tissue macrophages

### Research history and milestone events of study on macrophages

**Fig. 1:**
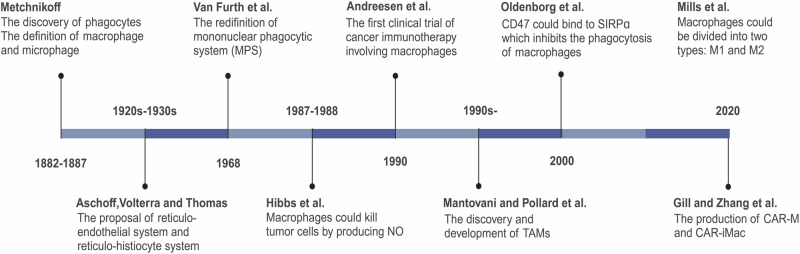
A timeline of research history and milestones of macrophages. NO Nitric Oxide, TAMs tumor-associated macrophages, CAR-M CAR-macrophage, CAR-iMac induced pluripotent stem cells (iPSCs)-derived CAR-expressing macrophage cells

In 1882, experimental pathologist Elie Metchnikoff found that a group of mobile cells around the rose thorn-pierced starfish larvae could quickly clear foreign material. Under the suggestion of zoologist Claus, he named them “phagocytes”, which was considered the discovery of macrophages, and eventually published his first paper on phagocytosis in 1883^45^ (Fig. 1). In 1887, Metchnikoff classified phagocytes into two populations: macrophages and microphages (later known as neutrophils).^46,47^ Since then, a series of concepts about the mononuclear phagocytic system (MPS), such as the “reticulo-endothelial system” of Aschoff and the “reticulo-histiocyte system” proposed and reintroduced by Volterra and Thomas, have been published. However, both of them are proven limited and incorrect several years later.^47,48^

In 1968, based on the similarities in origin, morphology, function, and kinetics of the phagocytes, Ralph van Furth et al. redefined the concept of MPS. They excluded the reticular cells, endothelial cells, dendritic cells, and fibroblasts from the MPS and proposed that macrophages mainly arise from monocytes derived from myeloid progenitor cells.^48^ Over the past few decades after the proposal and amendment, macrophages were thought to have lost their differential and proliferative potential, only to be continuously replenished by circulating monocytes in the blood. However, from the 1990s to 2010s, scientists realized that there is an embryo-derived macrophage lineage with completely different characteristics in heredity, development, and function compared to those derived from HSCs and circulating monocytes.^3,49,50^ These macrophages are termed “tissue-resident macrophages” (TRMs) due to their persistence in the body and close association with specialized tissue cells.^3^

Colony stimulating factor-1 (CSF-1), also known as macrophage colony-stimulating factor (M-CSF), was discovered by Bradley et al. and purified by Stanley et al. It is the first cytokine identified to stimulate hematopoietic cells to differentiate into macrophage colonies.^51–53^ In 1987–1988, Hibbs et al. found that macrophages could kill tumor cells by producing nitric oxide (NO), a product of arginine metabolism.^54,55^ In 2000, Oldenborg et al. demonstrated that CD47, an important ‘self’ marker on the cell surface, could bind to SIRPɑ on the surface of macrophages, generating a series of signaling cascades that inhibit the phagocytosis of macrophages.^56,57^ In 2000, Mills et al. classified macrophages into two subtypes, M1 and M2, based on their differences in metabolism, secretion, and function,^58,59^ laying the foundation for a series of follow-up studies on macrophage polarization. Between the 1990s and 2010s, Mantovani and Pollard et al. detailed the role of macrophages in tumor growth, invasion and metastasis, angiogenesis, and immunosuppression.^60–64^

In 1990, Andreesen’s team used monocyte-derived macrophages (MDMs) on 15 advanced cancer patients who had failed in conventional treatments. It is the first clinical trial of cancer immunotherapy involving macrophages. The results showed that while the primary tumor tissue did not completely disappear, some patients remained stable within six months after treatment. Importantly, no serious adverse events were found except for low fever and discomfort at the intraperitoneal injection site. However, challenges such as the failure to transport macrophages to the tumor site or the lack of plasticity of macrophages leads to a rapid loss of their anti-tumor phenotype.^65,66^ In the following decades, researchers explored numerous methods to enhance the efficacy of macrophage-associated therapies, including combining them with other treatments and applying new techniques such as gene editing. In 2020, Gill et al. engineered human macrophages using chimeric antigen receptor (CAR) to direct their phagocytic activity against tumors. CAR macrophages (CAR-M) exhibited the antigen-specific phagocytosis and ability to clear tumor in vitro, and it was further shown to induce a proinflammatory TME and enhance anti-tumor T cell activity in humanized mouse models.^67^ Besides, in 2020, Zhang et al. developed induced pluripotent stem cells (iPSCs)-derived, CAR-expressing macrophage cells(CAR-iMac) by introducing CAR into hiPSCs and making it differentiate into macrophages. This study showed that CAR-mediated signaling could significantly improve the ability of CAR-iMac to engulf tumor cells and lead to the transformation of CAR-iMac from M2 to M1 type in the presence of specific antigens such as CD19.^68^ In 2020, the FDA approved a Phase I clinical trial, NCT04660929, aimed at treating tumor patients with relapsed/refractory HER2 over-expression with anti-HER2 CAR macrophages (CT-0508, CARISMA Therapeutics). It is the first trial to study the effects of adenovirus transduction CAR-M in humans.^69^

### Developmental origins of macrophages

Macrophages’ origin, migration, and development is a complex process with high similarity between humans and mice.^11,70^ As described earlier in the MPS section, from the beginning, it was thought that macrophages in the human body’s tissues were entirely derived from the HSCs. However, a series of studies showed that macrophages in adult tissues were mainly derived from the yolk sac or fetal liver during the embryonic development.^3,11^ Mouse fate mapping studies have shown that yolk sac-derived EMPs have at least two distinct waves at the origin: at embryonic day 7.5 (E7.5), the yolk sac produces the first wave of EMPs, and they differentiate into macrophages in situ. Then macrophages migrate into the brain rudiment and become the major source of microglia.^8,71–73^ The yolk sac at E8.25 produced the second wave of EMPs. Firstly, this wave of EMPs migrates into the fetal liver. In the fetal liver, EMPs could differentiate into EMP-derived macrophage precursors (PreMacs), which could further develop into heterogeneous TRMs, such as epidermal Langerhans cells and liver Kupffer cells, after transmitting with blood and colonizing in various tissues(except brain tissue).^3,8,72–76^ EMPs disappear during fetal life, but TRMs and some mast cells persist and self-renew in the adulthood.^3^

The definition of TRMs and whether they could originate from HSCs remains controversial. Except for one study, the existing fate-mapping studies have shown that fetal HSCs derived from the Aorta-gonad-mesonephros region could not produce macrophages.^3,50,77–79^ Besides, multiple experimental evidence indicates that TRMs are locally self-renewing long-lived cells in tissues. In contrast, short-lived HSC-derived macrophages rely on circulating monocytes for renewal and could expand massively when receiving stimuli.^3^ Several studies about experimental brain inflammation (autoimmune encephalitis (EAE) and stroke) have shown that monocytes and bone marrow monocyte-derived macrophages (BMDMs) recruited in the brain mediate inflammation and gradually disappear during inflammatory remission. In contrast, microglia do not mediate inflammation and persist consistently.^77,80^ However, the following two examples may support TRMs’ HSC origin. Osteoclasts in the embryonic period contain EMP-derived nuclei and self-maintain in adult bones, and they could integrate HSC-derived nuclei by fusion, resulting in individual adult mouse osteoclasts being a chimera containing nuclei of EMPs and HSCs.^3,81^ Besides, gut lamina propria is replenished by HSCs and BMDMs, which have the property of self-maintaining.^3,82–84^

### Classically activated and polarized macrophages

**Fig. 2:**
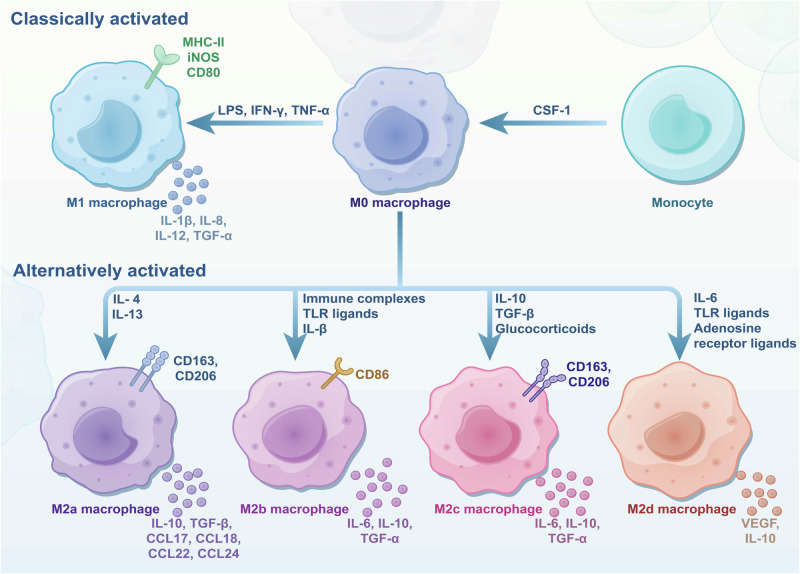
CSF-1 could induce monocytes to differentiate into M0 macrophages. Then, M0 macrophages could further evolve into M1 or M2 macrophages stimulated by Th1-type or Th2-type cytokines. Due to their differences in activation patterns and other aspects, M2 macrophages could be further divided into four subtypes: M2a, M2b, M2c, and M2d. Various macrophage types express different molecular markers and secrete different materials, which play important roles in various physiological and pathological processes. CSF-1 colony stimulating factor-1, MHC-II major histocompatibility complex class II, iNOS inducible nitric oxide synthase, LPS Lipopolysaccharides IFN-γ interferon-gamma, TNF-ɑ tumor necrosis factor-alpha, IL-1β interleukin-1β, IL-8 interleukin-8, IL-12 interleukin-12

Macrophage polarization is a process in which macrophages produce specific phenotypes and functional responses to microenvironmental stimuli and signals.^85,86^ In 2000, Mills et al. classified macrophages into two types, M1 and M2, according to their differences in activation patterns and functions, etc.^59^ (Fig. 2). CD68 is a marker expressed by all monocytes and tissue macrophages.^87^ Firstly, monocytes are stimulated by CSF-1 to form M0 macrophages. Then, M0 macrophages differentiate into M1 macrophages upon activating lipopolysaccharide (LPS) and Th1-type cytokines such as IFN-γ and TNF-ɑ. In contrast, M2 macrophages are formed by activating Th2-type cytokines such as IL-4, IL-10, and TGF-β.^88^ In addition to the activation patterns, there are other differences in receptor expression, cytokine production, and functions between these two types of macrophages.

From the perspective of signaling pathways, the polarization of macrophages is primarily governed by several key pathways. The TLR4/NF-κB^89^ and IFN-γ/JAK/STAT1^90^ pathways predominantly drive M1 polarization, activating pro-inflammatory profiles in macrophages. Conversely, the PI3K/Akt/mTOR,^91^ TGF-β/Smad,^92,93^ NOTCH,^94^ Wnt/β-catenin,^95–98^ IL-4/IL-6/JAK/STAT3^99,100^/STAT6,^101^ TRAF3/STAT6,^102^ and Hedgehog^103^ pathways mainly dictate M2 polarization, promoting anti-inflammatory and pro-tumoral activities. Research has shown that PI3Kγ, a critical isoform of PI3K,^104^ promotes M2 macrophage polarization through MTORC1-dependent activation of CCAAT enhancer binding protein β (CEBPB) and integrin subunit α 4 (ITGA4).^105,106^ This action inhibits the key pro-inflammatory transcription factor NF-κB signaling pathway,^107^ thus contributing to an immunosuppressive TME. In colorectal cancer, tumor cells release IL-4 to promote the expression of CD155 on TAMs,^108^ thus facilitating their M2 polarization and increasing the expression of IL-10 and TGF-β, thereby supporting tumor progression. In glioblastoma, CXCL8 supports the M2-like phenotype in TAMs through the CXCR2-JAK2/STAT3 axis,^109^ further highlighting the role of these signaling pathways in influencing macrophage behavior and impacting disease outcomes in different types of cancers.

M1 macrophages, also known as classically activated macrophages, mediate ROS-induced tissue damage and exhibit strong anti-microbial and anti-tumoral activity.^86^ These macrophages express or secrete markers such as MHC-II, iNOS, CD80, TGF-α, IL-1β, IL-8, and IL-12, contributing to their pro-inflammatory and immune-stimulating roles.^86,110^ On the other hand, M2 macrophages, often referred to as alternatively activated or immunosuppressive macrophages, are further classified into four subtypes based on different stimulatory factors: M2a, M2b, M2c, and M2d. Each subtype exhibits distinct surface markers and secretes specific cytokines and chemokines, playing crucial roles in inflammation resolution, tissue repair, and tumor progression.^86,88^

M2a macrophages, primarily induced by the cytokines IL-4 and IL-13, are one of the most widely studied subsets of M2 macrophages. First characterized in 1992, these macrophages are distinguished by the high surface expression of markers such as CD206, CD209, and Dectin-1. These are critical for recognizing and eliminating invading pathogens like bacteria, fungi, and parasites. Alongside these markers, M2a macrophages exhibit variable expression of CD14, CD163, and CD80/86, ranging from low to medium levels. Functionally, M2a macrophages secrete a variety of anti-inflammatory and tissue remodeling molecules, including IL-10, CCL17, CCL18, CCL22, CCL24, and the enzyme arginase 1 (Arg1), which plays a key role in amino acid metabolism and matrix reorganization.^111–113^ A key function of M2a macrophages is promoting tissue repair and remodeling. Upon tissue injury, IL-4 activates M2a macrophages, producing L-ornithine, a precursor for collagen and polyamines, essential for extracellular matrix (ECM) formation. M2a macrophages also secrete fibronectin and chitinase-like proteins, which aid in ECM reorganization and wound healing. However, fibronectin supports tissue repair and promotes tumor cell proliferation, invasion, and migration, contributing to tumor progression. Additionally, arginase-1 (Arg1) expressed by M2a macrophages depletes L-arginine, inhibiting T-cell proliferation and suppressing immune responses, while IL-10 further reduces pro-inflammatory cytokine production and T-cell activation. This immunosuppressive environment facilitates tumor growth, angiogenesis, and metastasis, making M2a macrophages and their secreted factors potential targets for cancer therapy.^114,115^

M2b macrophages, primarily activated by TLR agonists, immune complexes (ICs), and IL-1β, are known for their dual role in immune regulation and tumor progression.^86,116^ Unlike other M2 subsets, M2b macrophages produce high levels of anti-inflammatory cytokines, such as IL-10, while suppressing pro-inflammatory IL-12, facilitating a shift from Th1 to Th2 responses. They express CD86 and secrete IL-6, TGF-α, and CCL1, contributing to immune escape mechanisms and promoting tumor growth. The polarization of M2b macrophages requires two stimuli, typically ICs and LPS or IL-1β, which activate signaling pathways like NF-κB and PI3K/Akt. Their ability to suppress immune responses while promoting tissue repair and tumor progression highlights their unique regulatory function within the TME.^117,118^

M2c macrophages, stimulated by immunosuppressive molecules such as IL-10, TGF-β, and glucocorticoids, are characterized by CD163, MerTK, and CD206 expression. These macrophages secrete anti-inflammatory cytokines like IL-10 and TGF-β and chemokines such as CCL16, CCL18, and CXCL13,^119,120^ contributing to immune suppression and tissue remodeling. They play a key role in promoting tumor immune evasion and efficiently clearing apoptotic cells via MerTK-mediated phagocytosis.^116,121,122^ Additionally, M2c macrophages degrade the extracellular matrix through the secretion of matrix metalloproteinases (MMP7, MMP8, MMP9) and TIMP1, further aiding tissue repair and sustaining anti-inflammatory responses. Their ability to capture and sequester inflammatory chemokines through decoy receptors like CCR2 and CCR5 also highlights their immunoregulatory functions.^119,120^

M2d macrophages, first identified in the ascites of ovarian cancer patients, are polarized by IL-6 and leukemia inhibitory factor (LIF), with additional activation by adenosine receptor agonists and TLR agonists.^86,123–125^ They exhibit a typical M2 cytokine profile with high IL-10 and low IL-12, expressing markers like CD14, CD163, and TGF-β while showing low levels of CD86. M2d macrophages secrete VEGF, IL-10, and CCL18, contributing to tumor progression by promoting angiogenesis and suppressing T cell proliferation, thereby facilitating immune evasion and tumor growth.^126,127^

The plasticity of macrophages, often called ‘repolarization’ or ‘reprogramming,’ allows them to shift between these phenotypes in response to environmental cues.^128,129^ While this flexibility is crucial for mitigating inflammation and facilitating tissue repair in chronic infections, it can also promote tumor malignancy. The presence of M2 macrophages, with their immunosuppressive and pro-tumoral activities, is often associated with poor prognosis in various cancers. Thus, understanding each macrophage subtype’s specific markers and secreted factors provides key insights into their roles in both physiological processes and disease progression.

The M1/M2 polarization classification of macrophages is currently one of the dominant perspectives in the field and is widely applied to describe the functional roles of macrophages in various pathological conditions. The advantage of this classification is that it provides researchers with a clear framework to understand the dual roles of macrophages in immune responses. M1 macrophages are generally associated with pro-inflammatory and anti-tumor activities, capable of eliminating pathogens and inhibiting tumor growth by secreting inflammatory factors such as IL-12, tumor necrosis factor-alpha (TNF-α), and NO. On the other hand, M2 macrophages are involved in anti-inflammatory responses, tissue repair, and immunosuppression, secreting factors like IL-10 and TGF-β, which aid in tissue regeneration and immune regulation. Therefore, the M1/M2 classification offers an important directional guide for understanding the biological characteristics of macrophages in various diseases. This classification allows researchers to quickly identify the general functional tendencies of macrophages in a particular disease, especially in areas like inflammatory response, tumor immunity, and tissue repair, making it a foundational framework for macrophage research.

However, the limitations of this binary classification have become increasingly apparent, particularly in complex diseases such as cancer, autoimmune disorders, and cardiovascular diseases. While M1 and M2 polarization help researchers outline macrophage functions, the situation is far more complex. With the advancement of single-cell sequencing technology, we now recognize that macrophages exhibit significant heterogeneity across different disease microenvironments, and the simplistic M1/M2 classification fails to capture the full scope of macrophage functionality. Various macrophage subpopulations display distinct biological characteristics in different disease contexts, and the functional differences between these subtypes play a crucial role in disease progression and treatment response. Furthermore, the general M1/M2 classification may hinder the development of precision therapies in specific disease settings. For example, in the TME, some M2-type macrophages may both promote tumor progression and participate in tissue repair, while M1 macrophages, under certain conditions, may exacerbate inflammation or tissue damage. Therefore, although the M1/M2 classification provides a simple framework, its limitations in explaining and treating complex diseases have led researchers to adopt more refined subpopulation analyses. This more nuanced approach helps uncover the specific roles of different macrophage subtypes in various diseases, providing a critical foundation for personalized precision therapies.

### Heterogeneity, molecular markers, and phenotypic characteristics of macrophages

With the development of single-cell sequencing, lineage tracing, and other technologies, the heterogeneity of macrophages has been gradually understood and deciphered.^71,130^ Its heterogeneity is manifested in differences in many aspects, such as origin, distribution, stimuli, antigen expression, secretory factors and functions, etc.^130,131^ By using single-cell sequencing techniques, researchers could track and map the pathways of macrophage maturation from precursor cells, providing key insights into their origin and developmental processes.^11,50,132,133^ Furthermore, macrophages display high heterogeneity across different tissues and microenvironments, which is difficult to capture by traditional bulk transcriptome sequencing methods. Single-cell sequencing analysis allows for high-resolution analysis of gene and protein expression in macrophages, revealing unique subpopulations and functional characteristics in various locations, such as the liver, lungs, kidneys, brain, etc.^134–138^ Additionally, researchers could use a range of softwares or websites(such as CellChat, CellPhoneDB, etc.) to analyze single-cell sequencing data and construct interaction networks between different cell populations in the immune microenvironment, which could be applied for the identification of interactions between macrophages and other microenvironmental cells, as well as related secreted factors, ligands/receptors, and signaling pathways. These insights are crucial for understanding the roles and mechanisms of macrophages within the immune microenvironment.^139–141^

Human and mouse macrophages share a high degree of similarity in important functions such as phagocytosis, antigen presentation, inflammatory responses, and tissue repair, which makes the mouse an effective animal model for macrophage-related research.^142,143^ However, these two types of macrophages differ from one another in many aspects, such as the regulation of transcription factors, expression of surface markers, molecules involved in antigen presentation pathways, and drug responses, etc.^144,145^ For example, human macrophages express high levels of CD14 and human homolog of MHC-II(HLA-DR). In contrast, mouse macrophages could specifically express F4/80, Arginase-1(Arg-1) and Chitinase-like protein 3(YM1).^146–151^ Furthermore, the grouping of macrophage subtypes differs between humans and mice in different tissue and organs.^152^ Therefore, when designing experiments and analyzing data involving macrophages, it is essential to carefully consider the species-specific differences to ensure accurate and relevant results.

#### Macrophages in the bone

Bone macrophages could be divided into the following types: bone marrow macrophage (such as erythroblastic island macrophage, HSC niche macrophage, etc.), osteoclasts, and osteal macrophages (osteomacs).^153,154^

The erythroblastic island (EBI), the first hematopoietic niche to be discovered, primarily consists of a central erythroblastic island macrophage(EIM) surrounded by a cluster of immature erythroid precursor cells.^155,156^ HSCs-derived myeloid progenitors could differentiate into monocytes, and when monocytes enter the EBI microenvironment in the bone marrow, they further differentiate into EIMs.^155,157^ Research has shown that over 90% of mouse native EBIs are F4/80^+^ EPOR^+^ macrophages and EBI in the human fetal liver was also found to contain macrophages that express the EPOR.^157^ Besides, F4/80^+^ EPOR^+^ EIMs also express molecules such as CD169, VCAM1, Mertk, and DNase2α, etc., which are closely related to their functions.^157^The highly expressed adhesion molecules VCAM1 and CD169 (Siglec-1) on EIMs could bind to integrin ɑ4β1(VLA-4) and CD43, the ligands on the surface of erythroid precursor cells, respectively. This intermolecular interaction enables the EIMs to support the proliferation and differentiation of erythroid precursor cells.^158,159^ During the erythropoiesis, erythroid precursor cells undergo enucleation, where they shed their nuclei to become fully functional red blood cells.^160,161^ Mertk mediates the engulfment of pyrenocytes by EIMs, and DNase2ɑ is the key enzyme responsible for removing DNA left over from the enucleation of erythroid precursor cells, which enables EIMs to maintain a clean erythropoiesis environment for supporting the differentiation and maturation of erythroid precursor cells.^162,163^

Osteoclasts are present in the bone marrow, spleen, and blood. In bone, they are located in the resorption bays within the bone endosteum and specialize in bone resorption and remodeling by releasing proteolytic enzymes and acids.^164,165^ Of all the enzymes secreted by osteoclasts, tartrate-resistant acidic phosphatase (TRAP) is unique to osteoclasts and often serves as an important marker for identifying osteoclasts.^12^ Besides, the receptor activator for the Nuclear Factor-κb ligand (RANKL) is considered one of the most important factors in promoting the maturation and maintenance of the activity of osteoclasts. M-CSF could induce RANK expression on the cell membrane of the osteoclast precursor, which binds RANKL from osteoblasts, stromal cells, or T cells and produces an effect inducing osteoclast maturation and differentiation.^12,13^ As mentioned above, they are embryonically derived but could fuse with nuclei from HSC-derived macrophages, finally becoming multinucleated cells in adults. Defective osteoclast activity could contribute to osteopetrosis and bone marrow failure, while excess activity could result in bone loss and osteoporosis.^164^ Besides, an iterative fusion of circulating blood monocytic cells with long-lived osteoclast syncytia is crucial for the postnatal maintenance of osteoclasts, bone mass, and the bone marrow cavity.^164^

Osteal macrophages(OsteoMacs) originate from a resident population of macrophages and express typical macrophage markers such as CD68, F4/80(mouse), and CD169, but lack TRAP expression, which distinguishes them from osteoclasts.^166–169^ They are located adjacent to osteoblasts, osteoclasts, and dormant cells. They exhibit a stellate morphology that allows them to extend across bone surfaces, suggesting they may form an extensive communication network.^167^ OsteoMacs play an important role in bone formation and repair. They could regulate the activity of osteoblasts and the mineralization process of the bone matrix, but more experimental evidence is needed to support the mechanisms involved.^170^ Besides, it has been reported that they could also serve as immune surveillance cells in the bone microenvironment. This subset of macrophages is capable of clearing necrotic cells and debris through phagocytosis as well as responding to antigens.^168,171–173^ Notably, OsteoMacs support the maintenance of murine hematopoiesis by the megakaryocyte-induced up-regulation of Embigin and CD166.^174,175^

#### Macrophages in the peripheral blood

Monocytes, one of the precursors of macrophages, circulate in peripheral blood for approximately 1–3 days after producing and releasing by the bone marrow. Then, they migrate into tissues and subsequently differentiate into various types of macrophages or DCs.^176,177^ Human monocytes could be divided into three subsets: the classical (CD14^++^ CD16^-^), intermediate (CD14^++^ CD16^+^), and nonclassical (CD14^+^ CD16^++^).^177^ When incubated with GM-CSF or CSF-1, all three subsets acquired macrophage morphology, secreted cytokines associated with macrophages, and showed increased phagocytic activity.^178^ In mice, monocytes are categorized into two subsets: the Ly6C^high^ and Ly6C^middle^ subsets based on their Ly6C expression. The Ly6C^high^ subset, which perform pro-inflammatory functions and express high levels of CCR2, are more likely to differentiate into inflammatory M1 macrophages.Conversely, the Ly6C^low^ monocytes patrol along the vascular endothelium, participate in tissue repair, and tend to mature into M2 macrophages.^176^

#### Macrophages in the lung

In the homeostatic lung, there are several major macrophage populations separated by their anatomical location: alveolar macrophages (AMs) in the air-exposed space of the alveolus and two or three interstitial macrophage populations in the interstitial region of lung.^131,179^

Sialic acid-binding immunoglobulin-like lectin (Siglec)-F (SiglecF), a mouse cell surface glycoprotein, could be used to distinguish AMs from other types of lung macrophages, playing an important role in inflammation regulation, pathogen clearance, and the inhibition of autoimmune responses.^180–185^ AMs derived from Yolk sac EMPs and characterized with CD11c^+^ SiglecF^+^ CXC3R1^−^. They fill the alveolar spaces of the lungs after birth and are self-maintained in homeostasis.^12,186^ The production and maturation of AMs depend on GM-CSF and TGF-β-mediated induction of peroxisome proliferator-activated receptor γ (PPARγ), a transcription factor crucial for regulating lipid metabolism.^12,187–189^ AMs play an important role in the clearance of pulmonary surfactant, phagocytosis of inhaled particles, and immunosurveillance.^12,130^

The level of CX3CR1 expressed on monocytes is increased with maturation in bone marrow, which is inversely correlated with the Ly6C marker and CCR2 in the blood. Notably, CX3CR1 was reported to be associated with maturation, recruitment into specific sites, and immunomodulatory effects of monocytes and macrophages.^190–193^ Lung interstitial macrophages (CXC3R1^+^ CD11b^+^ SiglecF^-^) are located primarily between the epithelium and the capillary, which are crucial for immune surveillance in the lungs.^17,194^ They originate from monocytes in the embryonic stage, whose development is largely dependent on homeostatic CSF-1R signaling and could be supplemented by circulating monocytes in adulthood.^131^ They are rare in homeostasis but could increase significantly when faced with immune challenges.^194^ Besides, they could be further divided into two subsets (LYVE1^hi^ MHC-II^low^ and LYVE1^low^ MHC-II^hi^) or three subsets (based on the expression levels of FOLR2, CCR2, TIM4, LYVE1, and MHC-II).^131,136,195–197^ In addition, with the development and application of single-cell sequencing techniques, multiple studies have been conducted to investigate the heterogeneity and functions of macrophages in lung homeostasis and disease states, enhancing our understanding of lung macrophages and diseases associated with them.^136,137,198,199^

#### Macrophages in the liver

The macrophages in the liver include many populations: Kupffer cells (KCs), liver capsular macrophages (LCMs), central vein macrophages, lipid-associated macrophages, etc.^200^ KCs constitute the largest group of resident macrophages in the human body, accounting for approximately 80% to 90% of the resident macrophage population.^201,202^ They are derived from Yolk sac EMPs and line in the sinusoids of the liver in adulthood. KCs have various functions, with F4/80^+^, CLEC4F^+^, and TIM4^+^ recognized as their markers.^12,186^ C-type lectin domain family 4 member F (CLEC4F), an inducible C-type lectin, could be considered a characteristic marker of KCs.^1,203^ It could recognize and bind to specific carbohydrate structures, thus participating in the recognition and clearance of pathogens.^203,204^

KCs could remove cellular debris, senescent cells, and pathogens from the blood. Besides, They promote immune tolerance and are involved in the systemic metabolism of iron, cholesterol, and other lipids.^205^ Two distinct KC populations were reported in mice: KC1 (CD206^Low^ ESAM^-^ CD36^Low^) and KC2 (CD206^hi^ ESAM^+^ CD36^hi^). KC2 populations have higher metabolic activity, express high lipid and carbohydrate metabolism-related genes, and could regulate liver metabolism via the fatty acid transporter CD36.^206^ LCMs are monocyte-derived F4/80^+^ CX3CR1^+^ MHC-II^+^ cells and have been reported to join in neutrophil recruitment and immune surveillance.^201,207^ In addition, a single-cell RNA sequencing (scRNA-seq) study based on human liver tissue attempted to explore the grouping of intrahepatic macrophages, showing that there are two distinct populations of intrahepatic macrophages expressing CD68 across all liver samples analyzed.CD68^+^ macrophage population 1, characterized by high LYZ, CSTA, and CD74 expression, could represent inflammatory macrophages. As for CD68^+^ macrophage population 2, it is considered to have a tolerogenic function due to their high expression of MARCO and VSIG4, etc, which are associated with immune tolerance.^136^

#### Macrophages in the heart

Cardiac and pericardial macrophages are crucial in maintaining heart homeostasis and responding to pathological conditions.^208^ In recent research based on the scRNA-seq and other experimental technologies, human cardiac macrophages could be divided into three distinct groups according to the expression levels of CCR2 and HLA-DR: CCR2^+^ HLA‐DR^low^, CCR2^+^ HLA‐DR^hi^ and CCR2^‐^ HLA‐DR^hi^ cells.^209^ Similarly, in a recent study utilizing a combination of cell tracking and scRNA-seq, researchers identified four transcriptionally distinct macrophage populations in mice: TIMD4 cluster (TIMD4^+^ LYVE1^+^ MHC-II^low^ CCR2^-^), MHC-II cluster (TIMD4^-^ LYVE1^-^ MHC-II^hi^ CCR2^-^), CCR2 cluster (TIMD4^-^ LYVE1^-^ MHC-II^hi^ CCR2^+^), and ISG cluster (TIMD4^-^ LYVE1^-^ MHC-II^hi^ CCR2^+^) were observed.^210^ TIMD4 cluster operates independently of blood monocytes while maintaining the ability for self-renewal. In contrast, the MHC-II cluster shows partial replacement by monocytes, and the two CCR2^+^ subpopulations, the CCR2 and ISG clusters, are fully derived from monocytes.^210^ The population of TIMD4^+^ macrophages plays a key role in reducing inflammation and facilitating tissue repair through the phagocytosis of apoptotic cardiomyocytes.^211,212^ Moreover, they are crucial in regulating fibrosis and aiding in the repair process after cardiac injury, contributing to the maintenance of the structural and functional integrity of the heart.^209^ Notably, LYVE1 on macrophages plays a vital role in preserving vascular homeostasis by interacting with hyaluronic acid, which is expressed on the surface of smooth muscle cells.^213^

Beyond the heart tissue, the mouse pericardial cavity surrounding the heart is populated by two main macrophage populations: Gata6^+^ pericardial and MHCII^+^ pericardial macrophages.^208,214^ Gata6^+^ pericardial macrophages displayed transcriptional profiles similar to those of Gata6^+^ macrophages found in the peritoneal and pleural cavities, but differed from the profiles of resident cardiac macrophages.These cells express the transcription factor GATA6, which is crucial in regulating cardiac damage and preventing fibrosis after myocardial infarction.^208,214^ In contrast, MHCII^+^ pericardial macrophages, characterized by high levels of MHCII molecules, are primarily responsible for antigen presentation, which promotes adaptive immune responses. These macrophages assist in the recognition and clearance of damaged tissues during cardiac injury or inflammation.^208,214^

#### Macrophages in the spleen

In the spleen, at least four distinct subsets of macrophages are present in discrete anatomic regions: the red pulp, the white pulp, and the marginal zone separating the two, which have significant spatial, phenotypic, and functional diversities.^130,215^ Red pulp macrophages (RPMs) are thought to originate from the Yolk sac and fetal liver progenitors. In contrast, the other three subsets(marginal zone metallophilic macrophages, marginal zone macrophages, and white pulp macrophages) derive from adult bone marrow/blood monocytes.^12^ RPMs(F4/80^+^ VCAM1^+^ CD11b^Low^) could engulf and clear the senescent red blood cells, platelets, and other cells from the blood, which is vital for avoiding the development of autoimmune responses as well as the recovery of iron and heme.^1,27,216^ Noticeably, they rely on a series of transcription factors (Spi-C, NRF2, PPARγ, LXRɑ, and SREBP1) to regulate the active metabolism of iron and lipids, further regulate the homeostasis of splenic cells and red blood cells.^3,186^ Furthermore, VCAM1, a downstream molecule of Spi-C, has also been reported to be associated with immune regulation and iron metabolism.^217–219^ However, the effect of its expression on RPMs, particularly in relation to macrophage function, still requires further research.

A distinct CD169^+^ metallophilic macrophage subpopulation in the marginal zone of the mouse spleen could interact with the antibody-producing B lymphocytes and DCs, playing important roles in sinusoidal immunity.^220,221^ Besides, a MARCO^+^ SIGNR-1^+^ macrophage subpopulation in the outer section of the marginal zone is reported to participate in antigen capture.^222^ White pulp macrophages (WPMs) (CD68^+^, F4/80^-^) are located in B cell follicles of the white pulp, which are similar to tingible body macrophages in the germinal centers of lymph nodes,^222^ and they participate in the phagocytosis and clearance of apoptotic B cells.^130^

#### Macrophages in the lymph nodes

A group of sinusoidal CD169^+^ macrophages that resemble the metallophilic cells in the marginal zone of the spleen are located in the subcapsular sinuses of the lymph nodes, and they could deliver captured antigens to DCs for the activation of B and T lymphocytes.^130,223^ Medullary macrophages express CD68 and F4/80, the expression of which could be strongly enhanced by the phagocytosis of apoptotic lymphocytes. Notably, lymph nodes are often referred to as a graveyard for macrophages, as they undergo local turnover within this site.^130^

#### Macrophages in the intestine

In anatomy, the walls of both the large and small intestines could be subdivided into four layers: mucosa, submucosa, muscularis propria, and serosa (or adventitia in certain regions). The mucosa is further subdivided into three sections: the epithelium, lamina propria, and muscularis mucosae.^131,224^ Different macrophage subsets have been identified in different intestinal layers, with the lamina propria being the most abundant.^225^

Lamina propria macrophages (CD64^+^ MHC-II^hi^ CD206^+^) are in the mucosa lamina propria, located beneath the intestinal epithelial layer.^226,227^ They are initially fetal-derived and rapidly replaced by short-lived MDMs in a CCR2-dependent manner after birth and require live microbiota to thrive.^228,229^ Notably, Lamina propria macrophages are essential for gut barrier homeostasis. First, small intestinal lamina propria macrophages engulf surrounding material (such as apoptotic cells (ACs)) within the lumen and lamina propria, collect antigens, and support epithelial stem cell proliferation within intestinal crypts by providing Wnt ligands.^230–233^ Furthermore, macrophages in the lamina propria of the small intestine secrete large amounts of IL-10, which are critical for the induction of microbiota-specific regulatory T Cells.^229,234^

Besides, a group of long-lived macrophages could be found at the sub-tissular niches of the submucosa and the muscular external layers.^231^ The macrophages in the external layers express markers such as TIM4 and MHC-II and could be self-renewal in niches.^1,228,235^ Besides, they are close to blood vessels, as well as the submucosal plexus and muscular plexus, playing important roles in the maintenance of intestinal movement, as well as support for the growth and function of neuronal bodies in the enteric nervous system as well as blood vessels.^227,236–239^

#### Macrophages in the central nervous system

In the central nervous system (CNS), different populations of TRMs are found in defined anatomical locations: microglia in the CNS parenchyma and other macrophage subgroups located in the CNS interfaces, including ventricles, meninges, and perivascular space.

The mouse microglia have been reported to express F4/80, CX3CR1, and CD11b, which originate from Yolk sac EMPs without any contribution from HSCs in homeostasis.^71,236,240^ Specifically, a research indicated that CD45^-^ c-kit^+^ erythromyeloid progenitors in the yolk sac could be identified as the source of immigrating macrophages in the developing brain and represent the direct precursor of the definitive microglia population in the CNS.^241^ Besides, other two researches using mouse fate mapping deemed that microglia may derive from Runx1^+^ or CD206^+^ macrophages.^71,242^ Notably, In the brain, only microglia express marker CX3CR1.^241,243^ In addition, microglia could be distinguished from other macrophage subtypes in the CNS by a group of markers, including SALL1, P2RY12, TMEM119, and HEXB.^244^ These molecules have been reported to participate in the transcriptional regulation, development, and differentiation, inflammation regulation, as well as neuroprotection function of microglia.^245–250^ After their settle-down in the CNS, the embryonic microglia could be self-maintained through a cell-autonomous proliferation.^251,252^ Once planted in its tissue niche, microglia depend on CSF-1 receptor (CSF-1R) ligands locally released by histiocytes, primarily CSF-1 released by neurons and IL-34 released by astrocytes, to mature, thus performing specific functions during the CNS development and homeostasis.^253–255^ Notably, adult microglia are long-lived cells that maintain a stable network throughout their life cycle only with rare proliferation.^256,257^ They act as immune sentinels to protect the brain from pathogens. Besides, they could also maintain brain homeostasis through various mechanisms, such as scavenging ACs as well as regulating neurogenesis and synaptic activity.^258–262^

Brain perivascular macrophages (PVMs) are a group of macrophages localized in the perivascular spaces of CNS, including the leptomeningeal macrophages, stromal choroid plexus macrophages, etc., with distinct zonation and phenotype compared to microglia.^1,263^ Besides, compared to the single origin of microglia, the origin of other macrophages in the CNS is more diverse. Some researches support that PVMs, such as leptomeningeal macrophages, seem to be only derived from Yolk sac EMPs without the contribution of HSC-derived progenitors and circulating monocytes during adulthood.^242,264–266^ However, another research shows that stromal choroid plexus macrophages originate initially from embryonic EMPs, but could be postnatally replaced by circulating monocytes. In addition, intraventricular macrophages, including the Kolmer epiplexus cells, are of embryonic origin, while dural macrophages could be partialliy replenish by monocytes.^264,266^ Brain PVMs were reported to regulate cerebrospinal fluid (CSF) flow dynamics via the control of arterial motion, and the TIM4^+^ subgroup in them could promote proper dynamics of the ECM.^267^

It is worth noting that once the blood-brain barrier (BBB) is compromised, monocytes and macrophages from peripheral blood could also enter the brain.^268–270^

#### Macrophages in the skeletal muscle

The lineage tracing and bone marrow transplant experiment results demonstrate that mouse skeletal muscle-resident macrophages are CD11b^+^ F4/80^+^ CD64^+^. They originate from embryonic hematopoietic progenitors in the yolk sac and fetal liver and definitive HSC in the bone marrow of adult mice.^271^ By using single-cell sequencing technology, researchers have identified three different macrophage subpopulations in skeletal muscle: a population of locally self-renewing F4/80^+^ LYVE1^+^ TIM4^+^ macrophages (also named self-renewing resident macrophages) and two other populations F4/80 ^+^ TIM4^-^ macrophages and F4/80^Low^ CD11C^+^ MHCII^+^ cells that are monocyte-derived.^272^ It has been reported that local CSF-1 from fibro-adipogenic progenitors (FAPs) is essential for the survival of both TIM4^-^ monocyte-derived and TIM4^+^ self-renewing resident macrophages in adult skeletal muscle.^273^ Regardless of the muscle type, three transcription factor genes, Maf, Mef2c, and Tcf4, are differentially expressed by skeletal muscle macrophages.^271^ Notably, these macrophages could be important in maintaining tissue homeostasis and promoting muscle growth and regeneration.^271^

#### Macrophages in the kidney

There are two different kinds of macrophages in the kidney: kidney-resident macrophages (KRMs) and bone marrow-derived kidney macrophages (BMKMs).^274^ KRMs originate from Yolk sac EMPs, and the predominant markers in mice are CD64, F4/80, and CD11c.^275,276^ Besides, scRNAseq analysis and experimental results indicate that CD74 and CD81 may be potential cell surface markers for kidney resident macrophages in multiple species.^138^ Compared to those in other developing organs (brain, lung, and liver, etc.), kidney macrophages show increased expression of the transcriptional regulators Ahr, Irf9, Nfatc1, and Nfatc2, which are closely associated with the cytokine expression, secretion of NO and arginine, as well as activation of macrophages.^50,277–280^ KRMs could monitor and clear macromolecules, especially circulating immune complexes, which are transported through the capillary around the renal tubules. In addition, they may be involved in promoting renal vascular and ureteric bud branching development.^275,276^ Mouse KRMs showed metabolic quiescence in the homeostasis. In the lupus nephritis mouse model, the expression of OXPHOS and glycolysis genes was up-regulated, while the expression of fatty acid metabolism genes was down-regulated, suggesting that inhibition of this glycolytic switch by KRMs may be a therapeutic approach to control renal inflammation.^281^

#### Macrophages in white adipose tissue

Macrophages have been reported to play important roles in lipid metabolism, inflammatory responses, and energy expenditure in adipose tissue. Lean white adipose tissue (WAT) macrophages are predominantly derived from Yolk sac EMPs, and express markers like F4/80, CD11b, and CD206. Compared to macrophages in obese WAT, those located in lean WAT are generally metabolically quiescent, showing lower dependency on oxidative phosphorylation (OXPHOS).^3,12^ In obesity, mouse macrophages in WAT undergo dramatic remodeling in their state of cellular metabolism and function, leading to a pro-inflammatory state.^12,282^ Specifically, many factors induced by excessive lipid accumulation and hypertrophy of WAT, such as mechanical stress, hypoxia, etc., could stimulate macrophages in adipose tissue to secrete pro-inflammatory mediators, such as TNF-ɑ or IL-1β, which could further activate inflammatory pathways, such as JNK or IKKβ pathway in adipocytes. This mechanism is involved in many metabolic pathological processes or diseases, such as non-alcoholic fatty liver disease (NAFLD), insulin resistance, etc.^12,283,284^ Furthermore, inflammatory cytokines secreted by senescent cells could induce macrophages to proliferate and express the nicotinamide adenine dinucleotide (NAD)-consuming enzyme CD38, thus promoting a decrease in tissue NAD^+^ level during senescence.^285^

#### Macrophages in skin

Skin resident macrophages have two main cell types: Langerhans cells (LCs) and dermal macrophages (DMs). Fate-mapping studies revealed that skin LCs originate from embryonic fetal liver monocytes and yolk sac-derived macrophages.^80,240,286^ Adult epidermal LCs are live-long cells and could self-renew under homeostatic conditions. In contrast, endogenous LCs are replaced by monocyte-derived progenitors within their niche^287,288^ under severe inflammation, infection, or injury. Depending on transcription factors RUNX3, AHR, and ID2, LCs could further differentiate.^7,289^ By single-cell sequencing and mass-cytometry analysis of CD34^+^ HSCs derived human LCs obtained from cord blood, researchers successfully identified four distinct subgroups of human LCs:two steady-state subgroups LC1 (CD207^hi^, CD1a, EpCAM) and LC2 (CD207^low^, CD1b, CD1c, HLA-DR), as well as two activated-state subgroups activated LCs (aLC) (CCR7^low^, CD83, CD40), and migratory LCs (migLC) (CCR7^hi^, CXCR4).^290^ The LC1 and LC2 subgroups could be distinguished due to their distinct expression levels of Langerin (CD207), the CD1 family members (CD1a, CD1b, CD1c), and EpCAM.^291^ CD207 could be considered as a specific marker for LCs, which are involved in the capture, internalization, and presentation of antigens.^292–294^ CD83, a well-characterized marker of DC activation, could express on mature LCs and might be involved in T-cell activation.^295–297^ CCR7 could promote the migration of LCs to the lymph nodes.^298^ LCs play an essential role in skin immune surveillance and the maintenance of homeostasis. They are the only antigen-presenting cells in the epidermis that could migrate into draining lymph nodes after exposure to antigens and present antigens to T cells to initiate immune responses.^293^ In addition, LCs could reorganize the epidermal layer of the Keratinocytes by continuously isolating external antigens, keeping regulatory T cells in a steady state, thus controlling epidermal tolerance towards autoantigens and commensal microbiota, as well as the pattern of undifferentiated Keratinocytes in the suprabasal layers.^131,299–302^

DMs mainly derived from EMPs and could self-sustain in a CSF-1-dependent manner in a steady state. Besides, they could also be minimally supplemented by monocyte-derived macrophages after birth. It has been reported that several macrophage subsets with different anatomical locations and tissue functions have been identified in the adult dermis, such as sensory nerve-associated macrophages(CX3CR1^hi^, LYVE1^low^, and MHC-II^hi^) as well as vascular-associated macrophages (CX3CR1^low^, LYVE1^hi^, and MHCII^low^).^196,303,304^ Sensory nerve-associated macrophages could promote nerve regeneration after injury by degrading myelin in damaged fibers. Newly grown axons at lesion sites appear to recruit macrophages from other dermal sources, and these cells could acquire a sensory nerve-associated macrophage phenotype over time.^131,196,303^ In addition, vessel-associated macrophages are crucial for dermal blood vessel integrity, and the regulation of antifibrotic activity and immune cell recruitment.^196,304^

## Biological functions of tissue macrophages

**Fig. 3:**
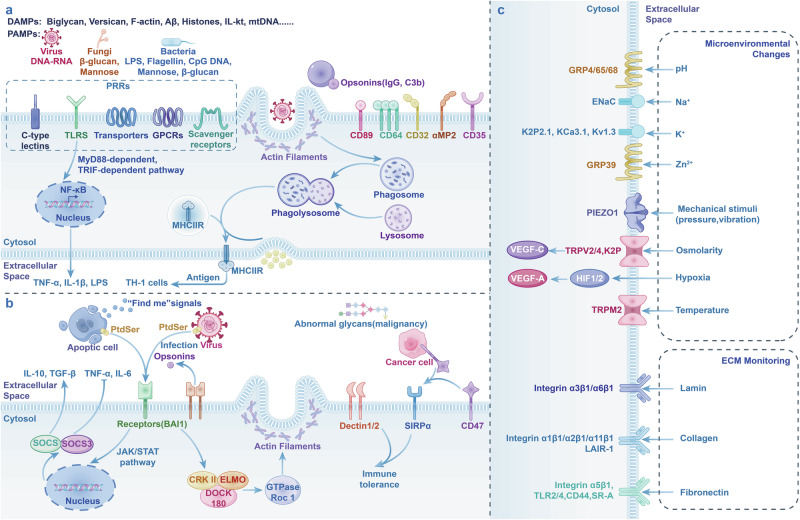
TRMs in inflammation and homeostasis. **a** The canonical pro-inflammatory response is initiated by either PRRs or opsonin receptors. PRRs can directly recognize DAMPs (usually cell-derived molecules, e.g., Biglycan, versican, F-actin) and PAMPs (usually microorganism-derived molecules, e.g., Foreign DNA, flagellin, mannose). The opsonin receptor-mediated recognition process involves binding foreign particles labeled by opsonins and opsonic receptors, including Fcγ receptors. The recognition activates actin polymerization, pro-inflammatory cytokines, and other responses. The phagosome fuses with the lysosome. In late endosomal MIICs, most newly synthesized MHC-II molecules are likely loaded in an HLA-DM-dependent mechanism. The antigens and MHC-II will form MHC-II peptide complexes and then be delivered to the plasma membrane to be available to stimulate antigen-specific CD4^+^ T lymphocytes. Pro-inflammatory cytokines (e.g., TNF-α, IL-1β, LPS). **b** Resolution of inflammation. Apoptotic cells can release “find me” signals (EG. ATP, Lys phosphatidylcholine, CX3CL1) to recruit TAMs. Phagocytosis is facilitated by receptors (e.g., BAI1, Mer, Axl, αvβ3-integrin) directly binding with eat-me signals (e.g., PtdSer, Calreticulin, LPC) on apoptotic cells or indirectly recognizing bridging molecules, such as MFGE8, C1q, protein S, etc., which bind to eat-me signals. The recognition activates actin polymerization, immune-resolution cytokines, and other responses. Pro-inflammation cytokines (TNF-α, IL-6, etc.) decrease, and immune-resolution cytokines (IL-10, TGF-β) increase. Some viruses display PtdSer on their surface and mimic apoptotic cells, which allows them to evade the immune system and facilitate entry into host cells. Tolerant responses have also been attributed to Dectin-1/2 and SIRPα. Altered glycosylation is a universal feature of cancer cells; some abnormal glycans (e.g., galectin 9) promote cancer growth and immune tolerance through Dectin 1/2 activation. Some tumor cells express CD47 that interacts with SIRPα, expressed on the surface of macrophages and dendritic cells, inhibiting phagocytosis and maintaining self-tolerance. **c** TRMs sense physical factors and cytokines in the microenvironment. SIRPα signal regulatory protein alpha, LPC lysophosphatidylcholine, PRRs pattern recognition receptors, PAMPs pathogen-associated molecular patterns, DAMPs damage-associated molecular patterns, MIIC MHC class II compartment, LPS lipopolysaccharides, LPC lysophosphatidylcholines, HLA human leukocyte antigen

TRMs play a pivotal role in many physiological processes, including clearance of cellular debris, inflammation and resolution, tissue remodeling, defense, and metabolic function. There are diverse channels or receptors on the cell membrane of TRMs that can sense microenvironment changes, making them respond to maintain tissue homeostasis (Fig. 3).

### Phagocytosis and clearance of cellular debris

Phagocytosis is the engulfment and clearance process of granule cell or cell debris, including microorganisms, foreign matter, senescent cells, damaged cells, and mutated cells. This function is predominantly in charge of professional phagocytes with high phagocytosis efficiency, including macrophages, neutrophils, dendritic cells, monocytes, etc.^305^ As the principal phagocytes, macrophage has the powerful function of phagocytosis and clearance of cellular debris. Accurate and specific recognition of the phagocytic target is macrophage’s first and foremost step.

Pathogen-associated molecular patterns (PAMPs) are molecular structures found on the surface or inside of microorganisms, such as bacteria, viruses, fungi, and parasites, such as LPS, peptidoglycan, viral RNA, unmethylated CpG DNA. Damage-associated molecular patterns (DAMPs) are the other critical part in the activation of the immune system. DAMPs originate from internal sources, which are usually tissue damage, trauma, ischemia, cancer, and autoimmune diseases. The examples of DAMPs include HMGB1, ATP, uric acid, heat shock proteins, DNA from necrotic cells. Macrophages can directly recognize PAMPs and DAMPs through surface pattern recognition receptors (PRRs) and opsonic receptors and introduce them into the cell through receptor-mediated endocytosis. The effect of recognition can be divided into two types. Some PRRs induce phagocytosis by recognition of PAMPs/DAMPs. Other PRRs can activate macrophage secreting proinflammatory cytokine but cannot trigger phagocytosis.

The PRRs with phagocytosis-inducing function mainly include C-type lectins (e.g., mannose receptor and dectins), scavenger receptors, and partial toll-like receptors (TLRs). Mannose receptor and scavenger receptor (SR) can effectively mediate macrophage phagocytosis, kill and eliminate pathogenic bacteria or apoptotic tissue cells through the recognition and binding of mannose/fucose residues on the surface of bacteria or fungi and LPS/lipoteichoic acid or phosphatidylserine on the surface of ACs.^306^ Controversially, whether the role of SR in inducing macrophage phagocytosis is direct or indirect is unclear.^307^ Scavenger receptor A (SR-A), MARCO, CD36, and CD14 are also in this question.^305^ In humans and other mammals, TLRs are divided into 10 main types (TLR1 to TLR10), each with a different extracellular domain that recognizes specific molecular patterns of PAMPs/DAMPs. As for the cytosolic domain, there are two main transduction modes, one is myeloid differentiation factor 88 (MyD88) dependent, and the other is MyD88 independent. The MyD88-dependent pathway is common to TLR signal transduction except TLR3. MyD88 is the main adaptor protein in the TLR signal transduction pathway. MyD88 then recruits IL-1R-associated protein kinase (IRAK) through DD, and then initiates downstream signal transduction through signal molecules tumor necrosis factor receptor-associated factor 6 (TRAF6), β-transforming growth factor-activated protein kinase (TAK1), TAK1 binding protein 1, 2 (TAB1, 2), activating nuclear factor (NF-κB) or activator protein-1 (AP-1), and finally inducing the expression of inflammatory cytokines such as IL-1, IL-6, IL-8, IL-12, TNF-α and other genes.^308^ Other PRRs without the capability of triggering phagocytosis can induce the production of inflammatory cytokines. After the sensation of lipoproteins of pathogens, including broad bacteria, fungi, and viruses, TLR2 can bind with TLR1 or TLR6 to form heterodimer for recognition of its ligands, namely triacyl and diacyl lipoproteins, respectively.^309^ Sequentially, the heterodimers induce macrophages and DCs to secret various inflammatory cytokines. Besides TLR1/2/6, the relatively functional specificity of TLRs is well identified (such as TLR4 & LP, MD2, DAMPs^310^; TLR3, TLR7, TLR8, TLR9 & nucleic acids of bacteria and viruses^311^). However, the mechanistic bridge of TLR recognition and macrophage phagocytosis is not perspicuously established, but phagocytic gene programs^307^ and a series of pathways are demonstrated in this process.^306^ TLR3 plays a key role in macrophage- hematopoietic stem and progenitor cells (HSPCs) interactions. High ROS levels within the HSPCs are associated with increased surface calreticulin (Calr), leading to macrophage phagocytosis, whereas low ROS levels allow HSPCs to continue dividing after interacting with macrophages. TLR3 signaling induces surface Calr in a manner that promotes “grooming” rather than cell death, indicating a protective effect on HSPCs.^312^

Another type of recognition pattern is mediated by opsonic receptors, among which antibody molecules (IgG) and complement components are well-researched. The recognition process involves the binding of foreign particles labeled by opsonins and opsonic receptors, including Fcγ receptors^313^ and complement receptors (CRs).^314^ Meanwhile, the effect of these receptors is not isolated, and there are interdependent interactions and cooperation between phagocytic receptors. For example, many receptors need to interact with several lgG.^315^ These scattered receptors are recruited and aggregated, resulting from the alteration of a well-flowing phospholipid bilayer, the effect of transmembrane glycoproteins and cytoskeleton^316,317^ (Fig. 3a).

Besides receptor-mediated endocytosis, pinocytosis is a type of non-phagocytic endocytosis that allowing cells to engulf and digest large particles or cells. Based on the vesicle size, there are two types of pinocytosis—macropinocytosis and micropinocytosis. Macrophages can intake larger target cells and cell debris by micropinocytosis, which usually helps in nutrient uptake, immune responses, and cell signaling. Under the trigger of specific stimuli, membrane folds are extended to form giant macropinocytosomes containing large amounts of extracellular fluid, nutrients, pathogens, soluble antigens, and liquid macromolecules into macrophage.^318^ Micropinocytosis is to uptake smaller vesicles that helps in the general maintenance of the cellular environment. Micropinocytosis occurs in most cells by three recognized mechanisms – clathrin-mediated endocytosis, caveolin-mediated endocytosis, non-coated vesicle endocytosis that is clathrin- and caveolin-independent.^319^

After the recognition, a series of signal pathways are activated to form a phagocytic cup, pseudopod, and phagosome. Emerging techniques are applied to measure phagosome dynamics, especially imaging and fluorescence-based methods in observing phagocyte formation.^320^ The forming of phagosome can be summarized in 4 steps (namely dynamic probing, receptor clustering induced by particle binding and receptor recognition, phagocytic cup formation, and phagosome sealing).^321^ Driven by Arp2/3-dependent construction of branched actin networks, membrane protrusions are extended. The hydrolysis of PI (4,5) P2^322^ and Rho-family GTPase^323^ seems to be determinate in actin remodeling during the phagocyte formation by activating WASP (Wiskott-Aldrich syndrome protein) and N-WASP for Arp2/3 complex activation.^324^ BAR (Bin-Amphiphysin-Rvs) domain-containing proteins can polarize actin and recruit scissor-associated proteins such as dynamin to facilitate phagosome sealing.^325,326^ Immunofluorescence localization shows that contractility of myosin, actin, and actin-binding proteins participate in phagocyte formation in macrophages. In the formation of macrophage phagosome, Myosins II and IXb, myosin IC, and myosin V were concentrated in the early stage, later stage, and phagosome sealing, respectively.^327^After forming new and early phagosome, phagosome maturation was performed to construct a hostile and degradative environment to facilitate phagocytic prey destruction.^328^ Phagosome maturation is a process where newly formed phagosomes undergo fusion and fission with endocytic organelles via a “kiss-and-run” mechanism.^329^ This interaction allows the phagosome to acquire necessary molecules for each stage of maturation. Eventually, phagosomes fuse with lysosomes to form phagolysosomes, which degrade the phagosomal contents.^330^ Subsequently, lysosomes form from complicated fission and fusion of phagolysosomes, early endosomes, and late lysosomes.^331^

Apoptosis is a form of programmed cell death crucial for organ development, tissue remodeling, regeneration, lesion healing, and homeostasis.^332^ The process of AC engulfment by responding phagocytes, predominantly macrophages, is called efferocytosis.^333^ Efferocytosis is a multi-stage apoptotic cell clearance mechanism, usually considered the final step of apoptosis. Efferocytosis is performed by phagocytic cells but distinct from phagocytosis and is crucial for the resolution of inflammation and tissue homeostasis. During apoptosis, dying cells release ‘find me’ signals to attract circulating phagocytes to their location for clearance. These ‘find me’ signals are diverse, which include nucleotides (ATP, UTP), lipids (LPC), chemokines (CX3CL1, S1P).^334–336^ To ensure that apoptotic cells are efficiently recognized and removed, ACs expose ‘eat-me’ singals on the outer leaflet of their plasma membrane. The most well-known ‘eat me’ signal molecule is phosphatidylserine (PtdSer), and others include calreticulin, oxidized lipids and altered/abnormal glycosylation.^337–340^ For preventing unwarranted phagocytosis of healthy cells, ‘do not eat me’ signals are essential to build up self-tolerance. Key ‘do not eat me’ signals include CD47, MHC I, and protective glycans^57,341–343^ (Fig. 3b).

As people age, they experience a gradual decline in overall physical function and accumulate more senescent cells. Senescent cells are cells that have stopped dividing and have entered a state of irreversible growth arrest, and untimely phagocytosis of them can lead to the breakdown of homeostasis, tissue deterioration, and tumorigenesis. Like ACs, these senescent cells also express ‘eat-me’ signals. Additionally, they secrete various bioactive molecules known as the senescence-associated secretory phenotype (SASP). These signals help macrophages recognize senescent cells and further clearance. However, it was found that senescent cells can upregulate the “do not eat me” CD47-QPCT/L axis to evade efferocytosis and inhibit macrophage-mediated clearance.^344^ Moreover, both senescent and aged macrophages exhibit impaired efferocytosis, contributing to STING signaling mediated inflammation.^345^

Apart from canonical roles of macrophages, TRMs in different tissue have additional roles. Splenic macrophages are specialized in facilitating blood filtration. For example, splenic red pulp macrophages (RPMs) are a specialized population which is derived from fetal monocyte. RPMs play a role in maintaining blood homeostasis and immune function, for example, taking up splenic red pulp by direct contact and phagocytosing blood-borne pathogens. In the clearance of eryptotic red blood cells, RPMs can recycle heme and iron from broken down senescent red blood cells.^346^ During this process, A transcription factor, Bach1, in red-pulp macrophages senses heme, an iron-containing product from erythrocyte degradation and ensure effective heme degradation by controlling HO-1 levels, a heme enzyme.^216^ The skin outmost layer is host to Langerhans cells, the primary immune cells in epidermis. Langerhans cells are marked with high CD207 (Langerin), EpCAM, MHC-II, and CD11c expression levels. Langerhans cells is important for skin immunity because they are known to migrate to the skin-draining lymph nodes after capturing antigens and undergo a maturation process, and then present antigens to T cells to initiate immune responses.^293^ They contribute to skin homeostasis because of their phagocytosis in cleaning up debris such as apoptotic keratinocytes^347^ and control regulatory T cells at steady state.^299^

### Antigen presentation and immune response

Antigen cross-presentation is vital for initiating adaptive immune responses against cancer, infections, and immune tolerance. TRMs, such as those in the liver (Kupffer cells), lungs (alveolar macrophages), and spleen (splenic macrophages), can capture extracellular antigens through phagocytosis and receptor-mediated endocytosis. They are professional antigen-presenting cells (APCs) that display peptides from internalized intracellular and extracellular antigens on major histocompatibility complex class I (MHC-I) proteins for presentation to T cells.^348^ The exact phenotype and antigen access of TRMs vary depending on the tissue. However, macrophages resident in the spleen, lymph nodes, liver, and peritoneum regularly encounter antigens carried by blood or lymph. This makes them optimal for antigen uptake and well-suited for cross-presenting to CD8^+^ T lymphocytes.^349^

In the spleen, macrophages are distinguished by their localization in the red and white pulp regions, which are demarcated by the marginal zone. Marginal Zone Macrophages (MZMs) and Marginal Metallophilic Macrophages (MMMs) are situated in the marginal zone. These macrophages are defined by their expression of sialic-acid binding immunoglobulin-like lectin 1 (CD169), macrophage receptor with collagenous structure (MARCO), and DC-Sign-related protein 1 (SIGNR1, CD209b). They are instrumental in capturing antigens from the bloodstream. Targeting antigens to metallophilic macrophages has been shown to facilitate the generation of cytotoxic T lymphocytes (CTLs) following the transfer of blood-borne antigens or adenovirus to CD8^+^ dendritic cells.^222,350^

In lymph nodes, macrophages can be categorized into several distinct subpopulations based on their anatomical locations. TAMs in the subcapsular sinus are identified as F4/80^−^ CD169^+^, while those in the medullary sinus are identified as F4/80^+^ CD169^+^, and medullary cord macrophages are dentified as F4/80^+^ CD169^−^.^351^ These macrophages are directly exposed to lymph fluid, enabling them to effectively capture lymph-borne antigens for presentation to T cells. In vivo studies have demonstrated that when a nanogel containing a tumor-specific synthetic long peptide antigen (LPA) and a TLR9 agonist is administered, F4/80^+^ CD169^+^ MSMs and F4/80^+^ CD169^−^ MCMs exhibit cross-presentation capabilities. This is evidenced by their ability to induce antitumor responses through the activation of tumor-specific CD8^+^ T lymphocytes.^352^ Further research indicates that CD169^+^ macrophages residing in lymph nodes can phagocytose apoptotic tumor cells and are crucial for the initial activation of tumor antigen-specific CD8^+^ T cells. Moreover, CD169^+^ CD11c^+^ macrophages demonstrate superior cross-presentation compared to CD169^+^ CD11c^−^ macrophages and CD169^−^ CD11c^+^ CD8^+^ dendritic cells.^353^ Tonsils harbor CD11c HLA-DR CD14^+^ cells, which have been identified as macrophages rather than DCs. These tonsillar macrophages efficiently phagocytose fluorescently labeled necrotic cells, as confirmed by flow cytometry.^354^ However, in vitro studies involving MelanA and NS3 antigen cross-presentation reveal that these macrophages are less effective in cross-presentation compared to major dendritic cell subsets.^355^

FOLR2^+^ TAMs found in both healthy and malignant breast tissues. The density of FOLR2^+^ macrophages within tumors has been positively correlated with improved patient survival outcomes. A robust correlation exists between FOLR2 expression in tumors and various immune pathways, including T cell receptor (TCR) signaling, PD-1 signaling, and antigen processing.^356^ Additionally, macrophages engaged in these processes exhibit high TIM4 expression, a receptor known to modulate cholesterol metabolism in macrophages by suppressing type I interferon signaling and enhancing SREBP2 activation.^357^

TIM4 also directs the slow progression of phagosomes, preserving antigens for cross-presentation. The peritoneal cavity, where gut and ovarian tumors commonly metastasize, is primarily populated by two distinct classes of macrophages.^358^ Small peritoneal macrophages (SPMs) originate from bone marrow myeloid precursors, are sparse under normal conditions, but undergo significant expansion during inflammation and tumor advancement. In contrast, large peritoneal macrophages (LPMs) constitute the predominant population under steady-state conditions, originating from embryonic precursors.^84,359^ Function displays high TIM4 expression and is associated with better prognosis in patients. During initiation of primary tumors or early colonization of metastatic sites, TIM4-mediated uptake of tumor cells can induce specific transcriptional remodeling of LPMs, further prolonging the integrity of ingested antigens, facilitating cross-presentation, and finally inducing anti-tumoral CD8 responses.^360^

Kupffer cells show antigen cross-presentation and efficient CD8^+^ T-cell proliferation, similar to classical DCs from the spleen. Antigen cross-presentation by Tie2 CD11b^low^ liver endothelial cells and CD11b F4/80 Kupffer cells depend on intercellular adhesion molecule-1 rather than intracellular interferon-gamma.^361^ However, the function of Kupffer cell cross-presentation may be immunosuppressive, as they contribute to the induction of tolerance of orthotopic liver transplantation in rats.^362^

Kidney-resident macrophages hold their homeostatic ability to monitor and clear macromolecules transported across peritubular capillaries, particularly of small antigen-antibody immune complexes in a FcγRIV^-^ mediated recognition manner.^274^ Furthermore, they are potentially involved in kidney organogenesis by promoting proper vascular network assembly.^363^

### Regulation of inflammation and cytokine production

Tissue-specific macrophages are pivotal in promoting and resolving inflammation through the production and regulation of cytokines. When exposed to an inflammatory stimulus, such as an infection, macrophages can undertake several critical responses. Macrophages promote the recruitment of leukocytes to the infection site by secreting chemokines and various cytokines. Activating Vascular Endothelium: Through the release of TNF-α, macrophages enhance the activation of the vascular endothelium, thereby facilitating the ingress of leukocytes. Macrophages activate a range of immune cells, including natural killer (NK) cells, T cells, and B cells, by producing cytokines such as TNF-α, interleukin-6 (IL-6), interleukin-12 (IL-12), and interleukin-1β (IL-1β). Engaging in Adaptive Immunity: Macrophages also contribute to the activation of the adaptive immune system through antigen presentation and the production of additional cytokines. These mechanisms collectively enhance the body’s ability to respond to and manage inflammatory challenges.^25^

Granulocyte-macrophage colony-stimulating factor (GM-CSF, also known as colony stimulating factor 2, CSF2) is a cytokine that stimulates the production of various myeloid cell subsets in response to stress, infections, and cancers. For example, GM-CSF stimulates the functional activity of mature granulocytes and macrophages, enhancing their capacity under immune stress. The differentiation of megakaryocytic progenitors and erythroid progenitor cells can also be activated by GM-CSF, thereby influencing the production of platelets and red blood cells. Myelopoiesis refers to differentiating cells into the myeloid, non-lymphoid cell lineage. This process is initiated by binding GM-CSF to GM-CSFR on myeloid cell precursors, which triggers a cascade of signaling events downstream of the GM-CSFR. This cascade ultimately produces myeloid-specific transcription factors, including PU.1 and interferon regulatory factor 4 (IRF4).^364,365^ The critical role of GM-CSF in myeloid cells is demonstrated in GM-CSF-transgenic (Tg) mice, which exhibit significantly increased counts of myeloid subsets such as macrophages, neutrophils, and eosinophils compared to control mice.^366,367^ Furthermore, studies on GM-CSF-deficient (GMCSF^−/−^) mice reveal that while GM-CSF is essential for emergency myelopoiesis in response to infection, cancer, and stress, it is not required for basal myelopoiesis.^368,369^

Macrophages exhibit significant heterogeneity, with their functions and activation states influenced by their tissue-specific microenvironments. For example, intestinal macrophages help maintain gut homeostasis by sampling luminal contents and secreting anti-inflammatory cytokines like IL-10 and IL-1β to regulate the activity and function of regulatory T cells (Tregs)^370^ and Th17 cells,^371^ respectively. Macrophages expressing CX3CR1, a receptor crucial for tissue-specific migration and adhesion, are important in counteracting inflammatory responses and maintaining barrier integrity in the gut lamina propria and the mucosal layers. They present antigens to T cells in the gut-associated lymphoid tissues (GALT), such as Peyer’s patches and mesenteric lymph nodes, and release microbial products and cytokines such as IL-22 released.^25^ They uptake ACs and induce an anti-inflammatory phenotype through TGF-β and IL-10 production by macrophages, supplemented by cytokines produced by local fibroblasts. Inflammatory macrophages, recruited during tissue damage or infection, release pro-inflammatory cytokines such as IL-1β, TNF-α, and IL-6, crucial for initiating and sustaining immune responses.

In the typical pro-inflammatory response, broad metabolic adaptations are essential for TRMs to effectively perform their roles. Pro-inflammatory macrophages often rely on glycolysis, while anti-inflammatory macrophages increase their TCA cycle activity and fatty acid oxidation. These metabolic pathways are regulated by various signals, including cytokines like IL-4, which promote the anti-inflammatory phenotype.^372–374^ Additionally, heightened TCA cycle activity leads to the accumulation of α-ketoglutarate (α-KG). α-KG has been shown to attenuate canonical pro-inflammatory cytokine production by inhibiting the inhibitor of nuclear factor kappa-B kinase subunit beta (IKKβ) and to stimulate the production of canonical anti-inflammatory cytokines through the activation of the histone demethylase Jumonji domain-containing protein D3 (JMJD3).^375^

MicroRNAs (miRNAs) are small, noncoding RNAs that play crucial roles in gene regulation in animals and plants by binding to the mRNAs of protein-coding genes, thereby guiding their post-transcriptional repression. MiRNAs are important regulators of macrophage polarization. Certain miRNAs, such as miR-155 and miR-125a/b, are upregulated in pro-inflammatory M1 macrophages, enhancing the production of inflammatory cytokines. Conversely, miRNAs like miR-187 and miR-378-3p support the M2 phenotype, aiding in anti-inflammatory responses and tissue repair.^376,377^

Recent in vitro studies indicate that efferocytosis also stimulates the proliferation of pro-resolution macrophages, partially depending on pathways downstream of the TAM receptor Mer (also known as MerTK) and phagolysosomal degradation.^378^ Co-culturing ACs with either LysM^Cre^Mertk^fl/fl^ BMDMs or wild-type BMDMs treated with a phagolysosomal acidification inhibitor diminished BMDM proliferation.^378^ After phagolysosomal degradation of ACs, macrophages activate metabolite sensing pathways, including the nuclear receptor family of transcriptional regulators, which can suppress inflammation.^379–381^ For instance, macrophages from LXRα/β-deficient mice (Nhr1h3^−/−^ Nhr1h2^−/−^), which lack downstream activation of the LXR metabolite sensing pathway, exhibit increased expression of pro-inflammatory genes such as IL-1β in response to ACs.^382^

In conclusion, to restore homeostasis after inflammatory responses, pro-resolution macrophages are essential. The macrophages clear ACs,^380^ produce anti-inflammatory factors such as IL-10 and transforming growth factor beta (TGF-β).^383^ They inhibit leukocyte recruitment through several mechanisms matrix metalloproteinase (MMP).^384^ Further facilitation of tissue repair by producing growth factors and remodeling the ECM^385^ (Fig. 3b).

### Tissue remodeling and repair

Efferocytosis, mediated by TRMs, is critical for maintaining tissue homeostasis and enabling tissue remodeling and repair after injury. This process, which involves the clearance of apoptotic cells, is intricately linked to tissue renewal and the prevention of pathological conditions. Here, we summarize the roles of macrophage-mediated efferocytosis across various systems.

In the CNS, microglia, the resident macrophages, regulate tissue homeostasis and repair. Within the hippocampal dentate gyrus, microglia balance neurogenesis and apoptosis through efferocytosis.^386^ During retinal development, Morales et al. recently clarified the involvement of microglia in efferocytosis during retinal development and that microglia collaborate with Müller glia to facilitate this process.^387^ Microglias also prevent the degeneration of myelin, which covers neuronal axons and maintains their structural integrity. The myelin disorders could lead to neurodegenerative diseases such as dementia, Parkinson’s disease (PD) and Alzheimer’s disease (AD). In acute demyelination, microglia synthesize desmosterol to resolve inflammation, accelerate remyelination, and maintain axonal integrity.^388^ Dysregulation in myelin maintenance, often tied to signaling pathways like TGFβ1-TGFβR1, contributes to neurodegenerative diseases such as PD and AD through abnormal lipid metabolism. SRI-011381 hydrochloride, a small-molecule activator targeting these pathways has shown promise in addressing myelin-related disorders.^389^ In the visual system, certain scavenger receptors directly participate in regulating the circadian rhythm by clearing outer segments of photoreceptors via retinal pigment epithelial cells.^390^ While not strictly efferocytosis, this process involves scavenger receptors, suggesting a potential role for efferocytosis in circadian rhythm maintenance.

Macrophage-mediated efferocytosis is fundamental to immune regulation. In lymphoid follicles, apoptotic B cells activate follicular macrophages, transforming them into tingible body macrophages, which prevent autoimmune diseases.^391^ Similarly, large peritoneal macrophages execute efferocytosis to maintain peritoneal equilibrium and self-tolerance.^392^ During pregnancy, decidual macrophages perform efferocytosis to uphold homeostasis at the maternal-fetal interface.^393^

In the heart, macrophages play essential roles in development, repair, and remodeling. Resident cardiac macrophages expressing MHC-II facilitate arachidonic acid metabolism and efferocytosis, while their dysfunction can disrupt cardiac growth.^394^ Following ischemic injury, resident macrophages orchestrate inflammation to improve remodeling, while recruited macrophages influence infarct size.^395^ Overexpression of EphrinB2 enhances these reparative processes, while its deletion exacerbates cardiac dysfunction by impairing lymphangiogenesis.^396^ These findings highlight the dual roles of macrophages in both injury repair and pathological progression.

Kupffer cells, the liver-resident macrophages, are indispensable for liver repair. They secrete WNT ligands and hepatocyte growth factors, promoting the differentiation of hepatic progenitor cells into functional hepatocytes.^397^ The absence of Kupffer cells delays liver recovery significantly, highlighting their role in tissue regeneration.^397,398^

In bone tissue, efferocytosis is central to in the digestion and recycling of ECM. Osteoclasts, specialized macrophages located in bone dndosteum,^164^ mediate bone resorption by secreting enzymes like cathepsin K, while macrophages release IGF1 to stimulate osteoblasts for bone formation.^82,165,399^ Osteoclasts are recruited to resorption sites by TGF-β1 and secrete IGF1, thereby enhancing osteoblast activity and promoting bone formation in vivo.^400,401^ Bone marrow stromal cells also perform efferocytosis to regulate bone marrow homeostasis and mitigate bone loss.^402^ This coordinated activity ensures skeletal integrity and adaptation to physiological demands.

Macrophages also secrete soluble mediators that stimulate local stromal and progenitor cell proliferation, crucial for the repair process.^403^ Dermal macrophages are vital for skin wound healing.^404,405^ Subsets such as TIM4+ and MHC-II+ perivascular macrophages interact with sensory neurons to promote proper dermal innervation.^303,406^ During injury recovery, macrophages produce IGF1 and PDGF-CC to stimulate fibroblast activity and facilitate wound closure.^406,407^ Early-stage depletion of macrophages during skin wound repair in mice impairs normal re-epithelialization and vascularization. Depletion of macrophages in the later stages of tissue repair leads to fibrosis in both the skin and liver^398^. In muscle tissue, macrophages secrete paracrine factors like IGF1 and glutamine, enhancing satellite cell proliferation and myogenesis. Macrophages associated with muscle tissue secrete paracrine molecules such as IGF1,^408^ the metalloprotease ADAMTS1,^407^ and glutamine in response to injury. These molecules stimulate the proliferation and differentiation of muscle-resident stem cells (satellite cells) and promote myogenesis.^409^

Within the male genitourinary system, Sertoli cells function as specialized phagocytes responsible for preventing the accumulation of apoptotic germ cells in the seminiferous tubules through efferocytosis. Smoothelin-like 2 has been identified as a regulator of efferocytosis and lactate metabolism in mouse Sertoli cells to achieve homeostasis.^410^ Additionally, “find me” signals attract neutrophils, and neutrophil-mediated efferocytosis has been implicated in inflammation^411,412^ and colorectal cancer.^413^ Macrophage-mediated efferocytosis supports regeneration in various contexts, including neonatal heart repair,^414^ kidney recovery,^415^ peripheral nerve restoration,^416,417^ and etc.^418–420^ Evolutionary conservation of these functions is evident in limb regeneration in salamanders^421^ and tail fin regrowth in zebrafish,^288^ as well as for repair after injury in zebrafish^422^ and Drosophila.^423,424^ This underscores the universal importance of macrophages in tissue regeneration across species.

Macrophage-mediated efferocytosis is a cornerstone of tissue remodeling and repair, playing versatile roles across diverse systems. Its broad regulatory and regenerative capacities offer critical insights into both physiological processes and therapeutic potential.

### Multi-level regulatory signaling pathways/crosstalk involved in homeostasis and physiology

Endocrine organs maintain systemic balance by producing hormones that regulate target tissues, stabilizing deviations from set points. Similarly, tissue homeostasis ensures a steady state free of inflammation or damage. Examples of homeostatically maintained variables include cell number and composition within tissue compartments, ECM density, composition, and stiffness, as well as the volume, oxygen concentration, pH, temperature, and osmolarity of interstitial fluids.^292^ TRMs, alongside autonomic nervous system afferents, C-fiber nociceptors, and mast cells, function as key homeostatic controllers, sensing changes in the tissue microenvironment and modulating these variables. TRMs achieve this by detecting ECM signals, producing growth factors, and releasing cytokines to either promote ECM synthesis or facilitate its degradation.^275,293,294^ For instance, macrophages release proteases to degrade ECM components and clear cellular debris upon sensing damage signals, such as extracellular ATP, low pH, or ECM fragments.^271^ They also recruit immune cells like monocytes and neutrophils, critical for tissue disinfection and repair during early injury stages.^160,271^

Macrophages contribute to vascular homeostasis and repair by supporting angiogenesis and vascular remodeling.^295^ In hypoxic conditions, macrophages secrete proangiogenic factors like VEGF-A, regulated by HIF1 and HIF2, to promote endothelial cell formation.^293,296^ Conversely, retinal macrophages counteract excessive vascular growth by producing WNT ligands, maintaining the retinal vascular plexus.^297^

In the pulmonary system, alveolar macrophages maintain surfactant homeostasis by recycling surfactant lipids and proteins produced by alveolar type 2 epithelial cells.^298^ The homeostasis of pulmonary surfactant in the lungs is maintained by a delicate balance: alveolar epithelial cells secrete surfactant lipids and proteins, while alveolar macrophages remove these substances. Specifically, macrophages in the alveoli eliminate excess surfactant phospholipids and proteins, with PPAR-γ in alveolar macrophages sensing surfactant lipids.^102^ Innate immune cells in the lungs also respond to mechanical forces via mechanosensory ion channels (MSICs) (e.g., PIEZO1). Activation via PIEZO1 leads to the secretion of EDN1 and promotes HIF1-alpha stabilization and CXCL2 expression, mediating immune responses like neutrophilia and bacterial clearance during abnormal cyclical hydrostatic pressure^299^ (Fig. 3c).

Fever is a beneficial response in host defense, yet its underlying mechanism remains unclear. Elevated body temperature sensitizes transient receptor potential melastatin 2 (TRPM2), a Ca^2+^-permeable cation channel found in various immune cells, including macrophages. Depletion of TRPM2 in macrophages has been shown to reduce cytokine release and fever-induced phagocytic activity, possibly mediated through redox signals. However, the precise mechanism requires further investigation.^300^

TRMs sense osmolarity to regulate the growth of lymphatic vessels via VEGF-C.^301,302^ They also influence the proliferation and differentiation of various local parenchymal, stromal, and progenitor cells.^303^ The increased density and proliferation of the lymphatic capillary network are monitored by tonicity-responsive enhancer binding protein (TonEBP) within MPS cells that infiltrate the skin’s interstitial spaces. TonEBP interacts with the promoter region of the vascular endothelial growth factor-C (VEGF-C) gene (Vegfc), leading to the secretion of VEGF-C by macrophages.^300^ Additionally, macrophages sense hypoxia and produce VEGF-A to promote angiogenesis^425^.

Macrophages detect pH variations in their microenvironments through pH-sensing G protein-coupled receptor 65 (GPR65) and two other proton-sensing receptors, GPR4 and GPR68 (also known as ovarian cancer G protein-coupled receptor 1, OGR1). When exposed to acidic extracellular pH, GPR65 triggers an anti-inflammatory response, whereas GPR4 and GPR68 promote pro-inflammatory responses.^304–306^ A murine model study showed that deficiency in GPR65 enhances the recruitment of macrophages and neutrophils to the colon, accompanied by increased expression of pro-inflammatory mediators.^307^ Moreover, tumors in patients with obesity and multiple cancers like CRC and HCC exhibited increased GPR65 expression, which drives intensified macrophage signaling and tumor cell-derived acid production, and finally promotes accelerated tumor growth.^424^ These ion-receptors detect changes in the ionic environment, particularly during inflammation, tissue damage, or infection. Epithelial sodium channel (ENaC), expressed on macrophages, plays a role in detecting changes in sodium levels, particularly in high-salt environments such as inflamed tissues. Under cardiac oxidative stress fibrosis and maladaptive remodeling, ENaC can activate macrophage recruitment and M1 polarization.^426^ ENaC may be a critical molecule in promoting macrophage migration and polarization.^427^ K2P2.1, KCa3.1 and Kv1.3 in macrophages can sense environmental potassium. K2P2.1, a two-pore potassium channel regulates NLRP3 inflammasome activation by controlling potassium efflux and maintaining plasma membrane potential in macrophages. This channel promotes the secretion of pro-inflammatory cytokines like IL-1β and the activation of caspase-1 during inflammatory responses.^428^ KCa3.1, an intermediate conductance calcium-activated potassium channel, reduces plaque instability in advanced atherosclerosis by limiting macrophage polarization toward the pro-inflammatory M1 phenotype.^429^ Inhibiting KCa3.1 may also offer therapeutic potential in macrophage-related disorders such as asthma,^430^ multiple sclerosis,^431^ and stroke,^432^ where controlling inflammation is critical. Kv1.3 influences macrophages in acute liver injury (ALI) by regulating their migration and infiltration into damaged liver tissues.^433^ Kv1.3 regulates macrophage inflammatory responses in atherosclerosis by modulating the ERK/NF-κB signaling pathway.^434^ In microglia, Kv1.3 promotes a pro-inflammatory state in disease-associated microglia by interacting with immune signaling proteins like STAT1 and TLR2.^435^ ZnR/GPR39 responds to extracellular zinc and plays an anti-inflammatory role by enhancing IL-10 production.^436^ ZnR/GPR39 helps in controlling hepatic insulin receptor signaling and mitigating liver fibrosis and inflammation.^437^

After acute depletion of Kupffer cells, NOTCH, TGF-β family receptors, and LXR signaling pathways play crucial roles in repopulating liver macrophages to maintain cell population homeostasis. DLL4 regulates the NOTCH transcriptional effector RBPJ, activating poised enhancers that rapidly induce LXRα and other factors determining the Kupffer cell lineage 4.

Splenic red pulp macrophages (RPM) and bone marrow macrophages (BMM) play roles in degrading senescent erythrocytes and recycling heme-associated iron. The transcription factor SPI-C is normally inhibited by the transcriptional repressor BACH1 in both RPM and F4/80^+^ VCAM1^+^ BMM. During pathologic hemolysis that leads to the loss of RPM and BMM, excessive heme triggers BACH1 degradation and derepression of Spic in monocytes. This process generates new RPM and BMM to facilitate iron recycling.^116^

The tissue-specific roles of macrophages are integral to normal physiology. They aid in vascularization, pulmonary function, fever responses, osmolarity and pH regulation, and iron recycling, underscoring their centrality in maintaining homeostasis across diverse systems. These specialized functions highlight macrophages as indispensable players in both systemic and tissue-specific regulatory networks.

## Role of tissue macrophages in diseases

As pivotal cell types within the innate immune system, Macrophages perform multifaceted biological functions across various diseases. They serve as the primary line of defense and play crucial roles in maintaining tissue homeostasis, modulating inflammatory responses, and promoting wound healing. Recent studies have unveiled the dual roles of macrophages in disease progression, particularly in conditions such as cancer, cardiovascular diseases, autoimmune disorders, metabolic diseases, and neurodegenerative diseases. They influence disease development through various mechanisms, including regulating the immune milieu, promoting or inhibiting inflammation, participating in tissue repair, and affecting cell death pathways. In this section, we will focus on elucidating the specific mechanisms by which macrophages operate within these diseases and their diversity and plasticity throughout disease progression. This exploration aims to provide a foundational understanding of their complex roles in disease regulation.

### Macrophage and cancer

#### Macrophages in the tumor microenvironment

The TME is a habitat for tumor cells and typically features extensive cellular infiltration. Tumor cells engage in crosstalk with surrounding stromal and immune cells, reshaping the microenvironment.^438,439^ This interaction facilitates tumor immune evasion, angiogenesis, increased drug resistance, phenotypic plasticity, and co-adaptive evolution under environmental pressures.^440–442^ As a crucial component of the TME, Macrophages play a dual role in the progression of almost all types of cancers.^443^

Cancer, characterized as a distinct chronic inflammatory condition, chemotactically attracts a significant infiltration of macrophages known as TAMs^444^ during its progression. Granulocyte-Macrophage Colony-Stimulating Factor (GM-CSF) is the principal cytokine driving TAM recruitment to the tumor tissue. In the early stages of tumor development, low levels of GM-CSF induce effective chemotaxis and antigen presentation by DCs, exerting an anti-tumor effect. However, as cancer progresses into its later stages, GM-CSF levels gradually increase, promoting TAM recruitment and thus facilitating tumor progression.^445,446^ Moreover, GM-CSF also enhances TAMs’ production of IL-8, which generates lymphotoxicity and induces tumor cells to produce more GM-CSF, thereby exacerbating TAMs’ impact on shaping the microenvironment. Beyond GM-CSF, cytokines such as IL-17, IL-34, CXCL4, CXCL12, CCL2, CCL5, CCL20, and CSF2 also play significant roles in TAM recruitment, with other tumor-associated stromal cells participating in this process.^21,447–455^ Pan-cancer analysis has revealed that macrophages are significantly expanded in the blood of most cancer patients, and the number of macrophages is notably higher in patients who are non-responsive to immune checkpoint inhibitors compared to those who are responsive. More importantly, antigen processing and presentation ability is significantly downregulated in myeloid cells in the peripheral blood of cancer patients.^456^ Recent research has shown that tumor nucleotide metabolism can regulate TAM recruitment. Researchers have demonstrated that cytidine deaminase (CDA) in tumor cells aids in producing and releasing uridine diphosphate (UDP) and other uracil nucleotides. These nucleotides act as signaling molecules that bind to the P2Y6 receptor, predominantly expressed on TAMs. This interaction promotes TAM recruitment and an immunosuppressive phenotype, thereby protecting the tumor from cytotoxic T-cell infiltration and rendering it resistant to immune checkpoint blockade (ICB) therapies, such as anti-PD-1.^457^ Interferon-Induced Protein 35 (IFI35) recruits TAMs through the proteasomal regulation of non-classical NF-κB signaling via p105^458^ in glioblastoma.

#### M1-type macrophages in the tumor microenvironment

In the early stages of tumor development, when tumor cells have not fully “evolved”, and their ability to shape the microenvironment is not yet perfected, their surface ligands/receptors facilitate recognition and phagocytosis by macrophages, enabling effective antigen presentation. Furthermore, the early stages of a tumor are akin to damage to native tissues, and the induced inflammatory response facilitates the polarization of macrophages to M1, thereby secreting pro-inflammatory and immune-activating cytokines. In collaboration with Th1 cells, they effectively inhibit tumor progression by producing nitric oxide (NO) and reactive oxygen species (ROS).^459^ Chemokines produced by M1 macrophages, such as CCL5, CXCL9, and CXCL10, also recruit activated T cells and NK cells to exert anti-tumor immunity.^460^ Therefore, M1 polarization of macrophages is critically important in anti-tumor immunity. For instance, in ovarian cancer, overexpression of guanylate binding protein 5 (GBP5) not only promotes classic cell pyroptosis in ovarian cancer cells via the JAK2/STAT1 pathway but also induces the secretion of CXCL9/10/11, which promotes M1 polarization of macrophages to reverse the immunosuppressive TME.^461^ In tumors, knocking down the YTHDF2 protein (an m6A modification reader protein) can encourage macrophage and M1 polarization recruitment, thereby enhancing CD8^+^ T cell anti-tumor immunity.^462^ A novel platinum drug, naphplatin, can activate endoplasmic reticulum calcium release in macrophages, thus activating the MAPK p38 and NF-κB signaling pathways, which promote the reprogramming of M2-type TAMs to M1-type macrophages with anti-tumor effects.^463^ In hematological malignancies, polarizing TAMs to M1 is crucial for curbing tumor progression.^464^

#### M2-type macrophages in the tumor microenvironment

Cancer, characterized as a unique form of chronic, uncontrollable inflammation, predominantly involves M2-type macrophages in the composition of TAMs, with a smaller proportion of M1-type macrophages. However, TAMs exhibit high heterogeneity, meaning they often represent an intermediate phenotype between M1 and M2 types. Moreover, the presence of TAMs is closely linked to cancer staging and prognosis. Early-stage tumors predominantly harbor anti-tumor M1-type TAMs, while pro-tumor M2-type TAMs characterize later stages. Consequently, the ratio of M2 to M1 TAMs, known as the TAMs polarization index, has been recognized as a prognostic marker for cancer.^465^ Increasing evidence suggests that some TAM subgroups can express M1 and M2 macrophage genes,^466^ and the functional heterogeneity of TAMs is closely associated with their spatial distribution. Therefore, the spatial distribution of M1 and M2 TAMs provides a more accurate reflection of disease prognosis than merely assessing the presence of different TAM phenotypes in the TME.^467^ Furthermore, TAMs secrete various cytokines that promote the development of corresponding tumor phenotypes,^468^ such as direct secretion of growth factors that stimulate tumor growth.^469^ For instance, M1 macrophages upregulate inducible nitric oxide synthase (iNOS), which metabolizes L-arginine to L-citrulline and nitric oxide, creating a microenvironment unfavorable for tumor progression. In contrast, M2 macrophages exhibit an opposite metabolic pattern, where upregulated arginase (Arg) 1 metabolizes L-arginine into L-ornithine and polyamines, tumor-supporting factors.^470^ Additionally, TAMs secrete vascular endothelial growth factor (VEGF) and matrix metalloproteinases (MMPs) to remodel the extracellular matrix, thereby facilitating tumor invasion and metastasis.^471^ They also release TGF-β, Arg1, Indoleamine 2,3-dioxygenase (IDO), IL-4, and IL-10 to shape the overall tumor immunosuppressive microenvironment, thereby limiting the maturation of dendritic cells and the normal function of CD8^+^ T cells.^472^ TAMs also recruit Treg and Th2 cells by releasing Aryl hydrocarbon receptor (AhR), CCL17, CCL18, CCL22, and CCL24.^473^ At the same time, they decrease the expression of anti-tumor effector T cell (CTL, Th1) surface markers such as MHC-I, MHC-II, CD80, and CD86 while upregulating surface PD-L1 to facilitate immune escape by tumor cells.^474^

#### The transformation and regulation mechanisms of tumor-associated macrophage phenotypes

In the TME, as tumors progress and evolve under conditions such as hypoxia and through the reshaping of the immune and metabolic landscape, M1 macrophages gradually transition into M2 macrophages, thus fostering tumor development. This polarization is influenced by cytokines such as IL-4 and IL-13, secreted by Th2 cells, eosinophils,^475^ and basophils.^476–478^ Additionally, tumor cells further drive M2 polarization by secreting macrophage colony-stimulating factors (CSFs) and transforming growth factor (TGF)-β. After interacting with tumor cells, macrophages can undergo a phenotypic shift from expressing M1 markers to increasing expression of M2 markers such as Arg1 and CD163.^479^ Interestingly, even the death of tumor cells can promote M2 polarization within the microenvironment.^480,481^ In pancreatic ductal adenocarcinoma (PDAC), extracellular vesicles release the KRASG12D protein into the microenvironment during autophagy-dependent ferroptosis. Macrophages that ingest these vesicles undergo M2 polarization via the STAT3 signaling pathway due to the presence of this protein.^482^ Surprisingly, the β2-microglobulin (B2M) subunit of Class I Major Histocompatibility Complex (MHC-I), which is crucial for the function of CD8^+^ T cells, can interact with PIP5K1A in gliomas and promote the secretion of MYC-induced TGF-β1, leading to M2 polarization of TAMs.^483^ OAS1 (2’-5’-oligoadenylate synthetase 1) is a member of the interferon-stimulated gene family and plays a crucial role in antiviral processes. Interestingly, pan-cancer analysis has revealed significant differential expression of OAS1 across various tumors. Overexpression of OAS1 can result in CTL (cytotoxic T lymphocyte) dysfunction and promote M2 polarization of macrophages.^484^ Interestingly, TRIM56, an interferon-induced E3 ubiquitin ligase that is overexpressed upon double-stranded DNA stimulation and regulates the production of type I interferon via the stimulator of interferon genes (STING) pathway, has been found through pan-cancer analysis to promote M2 polarization of macrophages in gliomas. Additionally, it can serve as an immunological biomarker for glioma prognosis.^485^ The stiffness of the extracellular matrix also affects TAM polarization. In stiff matrices, tumor cells secrete more CSF-1, which promotes the accumulation of M2 macrophages.^486,487^ Viral infections can also influence TAM polarization; Epstein-Barr Virus (EBV) induces M2 polarization in nasopharyngeal carcinoma by producing Ataxia Telangiectasia and Rad3-related protein (ATR), which supports tumor progression.^488^ Other cells within the TME also contribute to the M2 phenotype transition of TAMs. Platelets, for example, can bind to TAMs through CD62P interacting with P-selectin glycoprotein ligand-1 (PSGL-1) expressed on TAMs, activating the JNK/STAT1 pathway, which promotes the transcription of C5 and the release of C5a, leading to a pro-tumoral phenotype of TAMs.^489^

The distinct microenvironmental localization of M1 and M2 macrophages,^490^ also significantly reflects their biological functions. Generally, macrophages are primarily located at the tumor core, at the interface between tumor cells and the stroma, and within the tumor stroma itself.^491^ M2-type TAMs tend to be positioned near blood vessels and necrotic areas,^492^ demonstrating high adaptability to the hypoxic tumor environment. They frequently interact with endothelial cells,^493^ promoting angiogenesis and assisting in migrating tumor cells towards invasive areas.^494^ TAMs also play varying roles in different types of tumors. In gliomas, TAMs predominantly foster tumor angiogenesis^492,495^ and promote glioma metastasis. Microglia exhibit strong pro-inflammatory effects.^496,497^ In lung cancer, TAMs mainly facilitate tumor growth and metastasis.^498^ In triple-negative breast cancer (TNBC), depletion of TAMs significantly reduces tumor growth, recurrence, and invasion.^478,499^ In pancreatic ductal adenocarcinoma (PDAC), TAMs primarily contribute to tissue fibrosis, creating an environment conducive to tumor cell invasion, metastasis, and immune escape.^500^ Reprogramming M2-type TAMs into M1-type has been proven to be an extremely effective tumor immunotherapy approach,^461,463,501^ offering new avenues for cancer treatment by altering the TME to stimulate an anti-tumor immune response.

Due to differences in ontogeny and/or local stimuli, the simple M1/M2 dichotomy cannot fully characterize macrophages for advancing research and precision clinical treatment. With the rise of pan-cancer analysis, single-cell sequencing, and spatial transcriptomics, several key molecules have been identified that play critical roles in defining functional subgroups of TAMs. These molecules help distinguish macrophage subtypes and uncover their diverse functions within the TME. SPP1 is closely associated with M2 polarization, particularly in lung adenocarcinoma, where it promotes tumor angiogenesis and supports TAM infiltration. TREM2 is linked to immunotherapy resistance in multiple cancers, including melanoma, where TAMs with high TREM2 expression exhibit immunosuppressive properties. Inhibiting TREM2 has been shown to enhance immunotherapy response and inhibit tumor growth. APOE and APOC1 are widely expressed in breast and lung cancers, playing important roles in lipid metabolism and highlighting TAMs’ significance in metabolic regulation. Inhibiting APOC1 can reprogram M2-type TAMs into M1-type, thereby enhancing the efficacy of anti-PD1 immunotherapy. VEGFA, a key factor in angiogenesis, is highly expressed in TAM subgroups that promote tumor growth by enhancing endothelial cell proliferation and migration. CXCL9 and CXCL10, chemokines highly expressed in M1-type TAMs, especially those associated with inflammation, promote T cell recruitment, boosting anti-tumor immune responses, and are often observed in patients who respond well to immune checkpoint inhibitors. COL1A1, COL1A2, and COL3A1 are associated with collagen production in TAMs, particularly in renal cell carcinoma and lung cancer, influencing matrix remodeling and invasive tumor growth and potentially affecting responses to immune checkpoint therapies. HMOX1, a molecule involved in heme clearance, is highly expressed in specific TAM subgroups, indicating its role in maintaining antioxidant balance within the TME. These molecules help differentiate M1 and M2 macrophages and reveal the functional diversity of TAMs across various cancers. Understanding these pathways may provide novel therapeutic targets for improving cancer immunotherapy.^502^

#### Metabolic differences between M1 and M2 macrophages

**Fig. 4:**
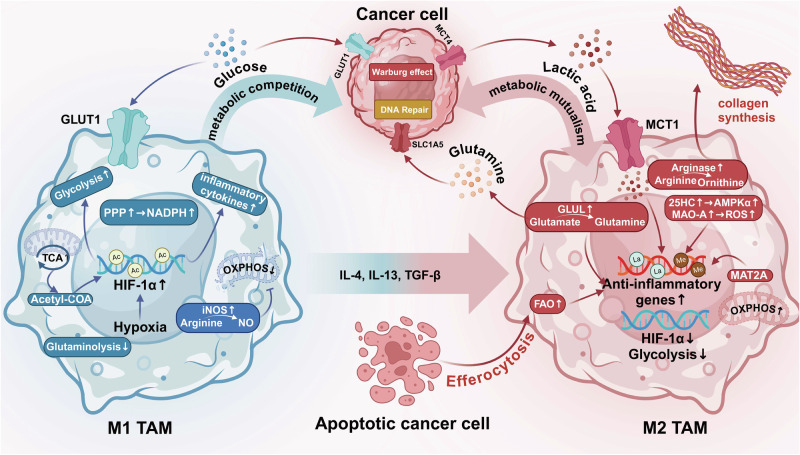
Metabolic differences between M1 and M2 macrophages. In the tumor microenvironment, the metabolic differences between M1 and M2 macrophages are closely linked to the onset and progression of cancer. Typically, both M1 macrophages and cancer cells primarily undergo glycolysis, leading to metabolic competition. As the malignancy of the tumor progresses, M1 macrophages in the tumor microenvironment are gradually reprogrammed into M2 macrophages, shifting their metabolism to oxidative phosphorylation. Furthermore, the exchange of metabolic products between M2 macrophages and cancer cells further promotes the manifestation of the tumor’s biological characteristics. This process highlights the complexity of the tumor microenvironment and underscores the crucial role of macrophages in tumor development. ppp pentose phosphate pathway, NADPH nicotinamide adenine dinucleotide phosphate (reduced form), HIF-1α hypoxia-inducible factor-1 alpha, OXPHOS oxidative phosphorylation, GLUT1 glucose transporter type 1, MCT4 monocarboxylate transporter 4, MCT1 monocarboxylate transporter 1, SLC1A5 solute carrier family 1 member 5, IL-4 interleukin-4, IL-13 interleukin-13, TGF-β transforming growth factor-beta, FAO fatty acid oxidation, 25HC 25-hydroxycholesterol, AMPKα AMP-activated protein kinase alpha, MAO-A monoamine oxidase A, ROS reactive oxygen species, MAT2A methionine adenosyltransferase 2A

From a metabolic perspective, M1 macrophages engage in anaerobic glycolysis and the pentose phosphate pathway to synthesize large quantities of proteins and fatty acids,^503^ whereas M2 macrophages prefer oxidative phosphorylation and fatty acid oxidation.^504^ Compared to normal macrophages, TAMs exhibit lower activities of glyceraldehyde 3-phosphate dehydrogenase (GAPDH) and succinate dehydrogenase (SDH),^505^ indicating that they require fewer nutrients to function within the TME, reducing their competition with tumor cells for glucose and thereby diminishing macrophage-tumor cell nutrient competition.^506^ High levels of glycolysis conducted by tumor cells can extensively deplete glucose in the microenvironment, inhibiting the anti-tumor activity of M1 macrophages. Furthermore, in gliomas, the reduced level of glycolysis in TAMs is often associated with poor patient prognosis.^497^ Although M2 TAMs show an increased tendency for glycolysis in breast and pancreatic cancers, even if they compete with tumor cells for nutrients like glucose, TAMs still favor tumor invasion and growth.^507,508^ Interestingly, tumor cells and TAMs form a compartmentalized metabolic unit where tumor cells secrete lactate and CSF-1. High lactate levels promote histone lactylation in macrophages, facilitating their transition to the M2 phenotype,^509–513^ while CSF-1R enhances the recruitment of circulating monocytes and their polarization towards M2 macrophages. Pan-cancer analysis revealed that lactate dehydrogenase LDHA is significantly overexpressed in various cancers and is closely associated with poor prognosis. Moreover, high LDHA expression is often positively correlated with macrophage infiltration and reduced antitumor activity of CD8^+^ T cells.^514,515^ Interestingly, research has unexpectedly found that the microbiome metabolite D-lactate (DL) can actually inhibit the PI3K/Akt signaling pathway and enhance the NF-κB signaling pathway, thereby reprogramming M2-type TAMs into M1-type, contrary to previous conclusions.^516^ Macrophages reciprocate by supplying tumor cells with growth factors such as epidermal growth factor (EGF), VEGF, interleukin-10 (IL-10), and matrix metalloproteinases (MMPs), thereby remodeling the tumor immune-suppressive microenvironment and promoting invasion and metastasis.^517^ Conversely, the metabolic relationship between tumor cells and cancer-associated fibroblasts (CAFs) is somewhat opposite.^518^ CAFs release large amounts of lactate into the matrix, which tumor cells take up and shift their metabolic mode from glycolysis to mitochondrial metabolism. CAFs even transfer healthy mitochondria to tumor cells to boost their mitochondrial function and invasion.^519^ The interaction between CAFs and TAMs also promotes the M2 polarization of TAMs,^520^ evidencing the cooperative adaptation and evolution of macrophages with surrounding stromal and tumor cells. Furthermore, studies show that TAMs upregulate the mTOR inhibitory regulator REDD1 under hypoxic conditions, reducing glycolysis and promoting tumor angiogenesis.^521^ However, overexpression of HIF-1α can stimulate glycolysis and PPP intermediates in macrophages, paradoxically inducing their polarization towards the M1 phenotype^522^ (Fig. 4).

M2-type TAMs exhibit high glutamine metabolic enzymes and transport proteins expression, indicating a high glutamine metabolism.^523,524^ Glutamine synthetase (GLUL), which converts glutamate to glutamine, facilitates the polarization of TAMs towards the M2 phenotype.^525^ Experimentally, depleting glutamine or inhibiting GLUL activity can effectively reduce the production of α-ketoglutarate, thus diminishing epigenetic modifications associated with macrophage polarization and ultimately inhibiting M2 polarization.^375,526^ This significant reduction in glutamine metabolism can even reprogram M2-type TAMs into M1-type. Furthermore, glutamine produced by TAMs can be absorbed by cancer cells through SLC1A5 and metabolized by GLS1 to counteract the damage induced by DNA-damaging agents.^527^ Inhibiting the fatty acid oxidation (FAO) of TAMs can also promote reprogramming from M2 to M1, which aligns with the metabolic characteristics of M2-type TAMs.^528,529^ Additionally, high-density lipoprotein can polarize M2-like macrophages to M1-like phenotypes by activating the MAPK p38 and NF-κB p65 pathways, mitigating immune suppression in the TME.^530^ Cholesterol metabolism also impacts the immunoregulatory capabilities of TAMs. Studies have found that 25-hydroxycholesterol (25HC) accumulation in TAMs alters the typical regulatory pathways of IL-6 and IL-13. 25HC promotes the phosphorylation of STAT6 by activating AMP kinase α (AMPKa), activating the STAT6-dependent signaling pathway, and promoting the expression of the anti-inflammatory gene Arg1.^531^ Interestingly, arachidonic acid can inhibit M2 polarization of macrophages, but its inflammatory metabolite PGE2, by inhibiting PPARγ, can indirectly enhance macrophage OXPHOS and thus promote M2 polarization.^532^ Recent research indicates that methionine metabolism also plays a significant role in TAM phenotypic transformations. Methionine adenosyl transferase II α (MAT2A) promotes the expression of RIP1 by facilitating the trimethylation of histone H3K4 (H3K4me3), which leads to the transformation of TAMs into the M2 subtype and contributes to the malignant progression of gastric cancer.^533^ Furthermore, the metabolic confrontation of nicotinamide between macrophages and cancer-associated fibroblasts (CAFs) can regulate the tumor immune microenvironment by modulating the activity of CD8^+^ T cells.^534^ In hepatocellular carcinoma, the absence of SIRT5, in conjunction with oncogenes, synergistically promotes the production of bile acids by liver cancer cells, thereby promoting M2 polarization of TAMs.^535^ Interestingly, monoamine oxidase A (MAO-A), typically active only in the brain, also promotes the immunosuppressive phenotype polarization of TAMs through oxidative stress responses.^536^

#### The relationship between macrophages and tumor development

##### Role in promoting tumor growth and metastasis

Cancer stem cells (CSCs) are often characterized by high expression of the anti-inflammatory gene NRF2 and by shaping a TME rich in TGF-β, thereby promoting chemotherapy resistance, metastasis, and recurrence. In glioblastomas, TAMs secreted TGF-β1 promote the growth of glioblastoma stem cells driven by the integrin αvβ5-Src-STAT3 signaling pathway.^537^ CD11b^+^ /CD163^+^ TAMs can also stimulate glioblastoma stem cell development by secreting pleiotrophin (PTN), which activates the protein tyrosine phosphatase receptor type Z1 (PTPRZ1) receptor on the surface of these cells.^538^ Recent studies have shown that cancer stem cells (CSCs) release large oncosomes (LOs) to mimic the presence of necrotic cells in the microenvironment, thereby “deceiving” macrophage precursors and inducing them to adopt an immunosuppressive phenotype. The activation of NRF2 signaling in CSCs promotes the translocation of IL-33 from the nucleus to the cytoplasm, where NRF2 also acts as a transcriptional activator to increase the expression of the membrane protein ATG9B. ATG9B encapsulates IL-33, forming large oncosomes that are released into the microenvironment. Interestingly, ATG9B, as a lipid transferase, creates a docking site for ANXA1 on the surface of the oncosome. ANXA1, a glucocorticoid-regulated anti-inflammatory protein, can be released extracellularly and mediate the resolution of inflammation. Additionally, ANXA1 binds to the membrane phospholipid phosphatidylserine (PtdSer), a well-known “eat-me” signal. The ANXA1 protein on the large oncosomes binds to FPR^+^ macrophage precursors in the microenvironment, driving their differentiation into immunosuppressive Arg1^hi^ CD206^+^ TAMs, further assisting CSCs in establishing a TGF-β-rich microenvironment.^539^ Pan-cancer analysis has revealed that Family with sequence similarity 109, member B (FAM109B) is significantly elevated across various tumor types and is associated with poor prognosis. Its expression is linked to aggressive progression and poor prognosis in low-grade glioma (LGG) patients, serving as an independent prognostic marker for LGG. Glioma grading is negatively correlated with FAM109B DNA promoter methylation. Additionally, immune infiltration and single-cell analysis show that FAM109B is significantly expressed in TAMs.^540^ Recent studies have shown that TAMs in the glioblastoma microenvironment, after engulfing cholesterol-rich myelin debris, transform into lipid-laden macrophages (LLMs), undergoing significant metabolic rewiring, accumulating large amounts of lipids, and altering their inflammatory response. The uptake of myelin debris not only triggers epigenetic changes in TAMs but also leads to the suppression of chromatin accessibility and immune activation-related genes, thus driving TAMs toward an immunosuppressive phenotype and weakening their anti-tumor immune function. LLMs transfer myelin-derived lipids to mesenchymal-like glioblastoma cells, supporting the high metabolic demands of the tumor, promoting its growth and proliferation, and managing lipotoxicity through the esterification of cholesterol. The LXR signaling pathway plays a key role in regulating cholesterol accumulation and export in LLMs, with lipid transporters ATP Binding Cassette Subfamily A Member 1 (ABCA1) and ABCG1 facilitating lipid efflux, which protects tumor cells from lipotoxicity and provides essential components for membrane construction. A symbiotic relationship forms between TAMs and mesenchymal-like glioblastoma cells, with tumor cells instructing TAMs to enhance their lipid scavenging abilities. TAMs provide the lipids necessary for tumor growth. This crosstalk is critical in the lipid-poor microenvironment of glioblastoma.^541^ In breast cancer, LSECtin on TAMs enhances the stemness of breast cancer cells by interacting with its receptor BTN3A3.^542^ TAMs also release the soluble glycoprotein NMB (GPNMB), which binds to the CD44 receptor on tumor cells, activating the expression of IL-33 and its receptor IL-1R1L, ultimately enhancing the stemness of the tumor cells.^543^ TAMs release exosomes containing lncMMPA, which interacts with miR-548s to increase mRNA levels of ALDH1A3, promoting tumor cell glucose metabolism and proliferation. Moreover, lncMMPA promotes M2 polarization of TAMs within the microenvironment.^544^ Under hypoxic conditions, TAMs increase the synthesis and secretion of galectin 3. The elevated galectin-3 promotes tumor growth through ROS generation and NF-κB activation.^545^ SnRNA/snoRNA-derived nuclear RNA 3, a major dicer-independent RNA, selectively inhibits transcription of Nos2 in macrophages, reducing iNOS expression by lowering chromatin accessibility at the Nos2 promoter and enriching Mi-2β and H3K27me3, thus promoting tumor growth.^546^ TAMs’ RON signaling activation promotes the secretion of IL-35, enhancing the growth of breast cancer,^547^ while their secretion of IL-6 also promotes breast cancer stem cell enrichment through STAT3 activation.^548^ Through integrated multi-omics analysis, JUN and its regulatory network positively correlate with TAMs and fibroblasts. Macrophage- and fibroblast-derived fibronectin 1 (FN1) can activate the Hippo pathway via JUN, promoting tumor metastasis. This mechanism may be universally present across various types of tumors.^549^ Moreover, TAMs facilitate tumor invasion and distant metastasis, which are closely linked with poor prognosis across various cancers. The collaborative interaction between cancer-associated fibroblasts (CAFs) and TAMs also promotes TME matrix remodeling. CAFs secrete CXCL14 and chitinase 3-like 1 (Chi3L1) to recruit and promote M2 polarization of TAMs. In return, M2 TAMs secrete MMPs and tissue proteases, degrading the ECM.^550^ TAMs also promote the growth of bladder cancer by stimulating the PI3K/AKT pathway through collagen.^551^ ECM degradation fragments further recruit TAMs, reshaping the ECM to optimize conditions for tumor cell metastasis and angiogenesis.^552^ In melanoma, the absence of TRIM59 in M2 TAMs, through upregulation of MMP9 and Madcam1, promotes tumor migration and invasion.^553^ In breast cancer, under the stimulation of TGF-β, TAMs promote the expression of collagen cross-linking enzymes such as lysyl hydroxylase 2 (LH2) and lysyl oxidase (LOX), which directly participate in collagen cross-linking, hardening the tumor ECM and facilitating invasion.^554^ Interestingly, TAMs at potential metastatic sites and those extravasating through endothelium re-arrange collagen in the ECM, creating micro-tracks that facilitate tumor cell invasion.^555^ Conversely, TAMs can spontaneously secrete collagen molecules and tightly cross-link them within the ECM, forming a physical barrier that impedes the infiltration and killing by external immune cells.^556^ Beyond reshaping the microenvironment, TAMs can further induce epithelial-mesenchymal transition (EMT) in tumor cells, promoting metastasis by releasing IL-1, IL-6, TNF-α, and TGF-β, which upregulate EMT markers in tumor cells such as vimentin and β-catenin.^557–559^

##### Role in inhibiting tumor growth and spread

While many TAMs exhibit an M2 phenotype that promotes tumor growth, the M1 subtype of TAMs serves as a critical mechanism for tumor elimination. Repolarizing M2 TAMs to the M1 phenotype may also represent a novel and effective anti-cancer strategy.^560^ In the context of ependymomas, a subset of inflammatory CCL2^+^ TAMs has been identified, characterized by high pro-apoptotic gene expression and immune response-related factors such as IL-1β, CCL3, and CCL4.^561^ This suggests that CCL2^+^ TAMs may be active in initiating immune responses by promoting inflammatory reactions and regulating tumor cell apoptosis, thereby influencing the TME. Interestingly, tumor cells release small amounts of the tumor suppressor factor PTEN into the microenvironment. PTEN binds to PLXCD2 on the surface of M2-type TAMs and activates the downstream JAK2/STAT1 signaling pathway, inducing the reprogramming of M2-type TAMs into M1-type. This reprogramming enhances the anti-tumor abilities of CD8^+^ T cells and NK cells.^562^ Additionally, studies have shown that macrophages can regulate nicotinamide metabolism in fibroblasts by secreting exosomes (EVs) containing NAMPT, which inhibit NNMT expression in fibroblasts through the SIRT1/NICD axis, thereby enhancing the cytotoxicity of CD8^+^ T cells.^534^ This cholinergic metabolic communication mediated by EVs enhances the efficacy of anti-PD-1 therapy. Furthermore, exosomes from M1-like macrophages expressing OX40L (CD134) (OX40L M1-exos) can effectively inhibit the progression of nasopharyngeal carcinoma by engaging the OX40/OX40L pathway and reprogramming M2-like TAMs into M1-like macrophages.^563^ TAMs with high enolase (PCB) expression exhibit enhanced phagocytic capacity, and PCB downregulates PD-L1 transcription via transcription factor IRF, promoting the infiltration of CD8^+^ T cells.^564^ Moreover, TAMs lacking the YTH N6-methyladenosine RNA binding protein F2 (YTHDF2) can be effectively reprogrammed into an M1 phenotype by IFN-γ-STAT1, exerting antitumor effects.^565^ Ferroptosis, a recently discovered form of cell death, has also been utilized in cancer treatment. Recent studies have shown that 1-stearoyl-2-15-HpETE-sn-glycero-3-phosphatidylethanolamine (SAPE-OOH) is an important surface marker of ferroptotic tumor cells and binds to TLR-2 on the surface of macrophages, promoting the phagocytosis of ferroptotic tumor cells by macrophages. However, ferroptosis inducers are not cell-specific and can lead to phospholipid peroxidation in both tumor and non-tumor cells. The accumulation of lipid peroxides in the endoplasmic reticulum (ER) of macrophages caused by ferroptosis inducers inhibits the transport of TLR2 to the plasma membrane. It leads to its retention in the ER by disrupting the interaction between TLR2 and its chaperone, CNPY3. Subsequently, the ER-retained TLR2 recruits the E3 ligase MARCH6 and initiates proteasome-dependent degradation.^566,567^ ScRNA-seq and pan-cancer analysis have revealed that CLDN18 is a key gene regulating antibody-dependent cellular phagocytosis (ADCP) immunotherapy in hepatocellular carcinoma. Its expression is closely associated with the infiltration of M1 macrophages in the TME of liver cancer.^568^ Additionally, based on extensive single-cell and spatial transcriptomic data, Proteasome Activator Complex Subunit 2 (PSME2) has been identified as a pan-cancer biomarker for M1 macrophage infiltration. In osteosarcoma cells, overexpression of PSME2 can significantly inhibit tumor proliferation, migration, and invasion activities.^569^ Interestingly, pan-cancer analysis has revealed that macrophage migration inhibitory factor (MIF) and CD74 are both significantly overexpressed in various cancers, with MIF serving as a marker for M0 macrophage infiltration and CD74 being a marker for M1 macrophage infiltration in the TME. Studies have shown that blocking the MIF-CD74 signaling pathway on macrophages and dendritic cells can effectively activate anti-tumor immune responses in “cold” tumors, such as melanoma. Additionally, CD74 expression significantly increases in certain cancers after receiving immune checkpoint blockade (ICB) therapy, strongly indicating that CD74 activation triggers immune responses in most cancers.^570,571^

##### Impact on tumor angiogenesis

Cancer fundamentally represents a chronic, uncontrolled inflammatory injury. Correspondingly, most TAMs predominantly exhibit M2-like functions, playing a role in counterbalancing and repairing the tissue damage caused by cancer. A critical step in tissue repair is angiogenesis,^572^ which ensures adequate nutrient supply by restoring blood flow to the damaged area. Similarly, the uncontrollable proliferation of tumor cells compresses the surrounding normal tissue, creating necrotic zones, and results in numerous avascular areas unless new blood vessels form promptly.^573^ Under the pressures of hypoxia and nutrient deprivation, tumor tissues can also undergo necrosis. Therefore, angiogenesis is a critical target in cancer therapy.^573,574^ Long-standing research has identified TAMs as accomplices in promoting tumor angiogenesis.^575,576^ For instance, TAMs associated with matrix remodeling in the TME are highly correlated with angiogenesis. In contrast, M1 pro-inflammatory macrophages tend to induce vascular quiescence. Consequently, reversing M2-like TAMs to an M1 phenotype can be highly beneficial for targeting tumor angiogenesis.^577–579^

TAMs secrete a plethora of angiogenic factors such as EGF,^580^ PDGF,^581^ PAF, VEGF,^582^ FGF2,^115^ and MYDGF,^583^ as well as inflammatory cytokines like TNF-α,^584^ IL-1,^585^ chemokines (CXCL8,^586,587^ CCL18^588^), and other pro-angiogenic activators including adrenomedullin (ADM),^589^ PGE2,^590^ thymidine phosphorylase (TP),^591^ and urokinase-type plasminogen activator (uPA). These factors induce endothelial cell proliferation and chemotaxis, thereby driving angiogenesis. Particularly, a subset of monocytes expressing Tie2, termed Tie2-expressing monocytes (TEMs), are a crucial source of angiogenic TAMs within tumor tissues.^592–595^ These cells are chemotactically attracted by angiopoietin-2 (ANG2) derived from tumor endothelial cells (EC),^594,596^ and their interaction with ECs stimulates further expression of Tie2 in TAMs. On the other hand, tumor ECs also secrete IL-6, which further promotes the M2 polarization of macrophages, thus facilitating tumor progression.^597^ Moreover, tumor cells can deliver Tie2 proteins to TAMs via exosomes.^598^ However, recent studies have found that Tie2-expressing TAMs may be dispensable for tumor angiogenesis and post-chemotherapy tumor recurrence.^599,600^ Under hypoxic conditions, HIFs^601–603^ promote transcription of vast amounts of VEGF, further regulated by transcription factors induced by IL-1β, such as STAT3 and NF-κB,^604–607^ and CCL18.^588^ Additionally, TAMs enhance pericyte coverage and increase vascular density by secreting PDGF-B and adenosine deaminase 2 (CECR1),^608^ further promoting vascular maturation. TAMs expressing lymphatic endothelial hyaluronan receptor 1 (Lyve-1) coordinate with αSMA^+^ cancer-associated fibroblasts (CAFs) in the tumor vascular microenvironment to create a conducive environment for angiogenesis.^581^ Neurotransmitters can also enhance tumor angiogenesis, with catecholamines promoting the M2 polarization of TAMs, thereby significantly increasing their expression of factors such as VEGFA.^609^ Blocking β-adrenergic receptors can effectively inhibit tumor growth and angiogenesis.^610^ Abnormalities in cholinergic metabolism also promote the M2 subtype polarization of TAMs and the extensive proliferation of endothelial cells, thus enhancing tumor angiogenesis.^611^ In glioblastoma, single-cell sequencing has identified a group of hypoxia-TAMs that respond strongly to hypoxic environments. These TAMs are activated by adrenomedullin-mediated paracrine signals, leading to unstable tumor vascular structures. This instability results in high vascular permeability, impacting drug delivery efficiency.^612^ Single-cell sequencing and SCENIC (Single-cell regulatory network inference and clustering) analysis of ependymoma cells have identified a group of CD44^+^ TAMs that highly express VEGFA driven by the transcription factor TEAD1, thereby promoting tumor angiogenesis.^561^ In head and neck squamous cell carcinoma, TAM-derived exosomes containing miR21-5p enhance tumor angiogenesis through the YAP1/HIF-1α axis.^613^ Tumor cells can also release exosomes containing miR-301a-3p, which, through the PTEN/PI3K/AKT signaling pathway, induces M2 polarization of macrophages and promotes angiogenesis.^614^ Annotation of single-cell data from various tumors has identified a new type of TAM, SPP1^+^ TAMs, highly associated with tumor angiogenesis.^615,616^ SPP1^+^ TAMs are identified by the specific expression of the core gene SPP1 and often express genes such as FN1, IL1RN,^617^ MARCO, and VEGFA.^618^ They were first discovered in colorectal cancer and later found in lung and breast cancers.^619,620^

Traditional therapies targeting tumor angiogenesis typically suppress vascular formation, hoping to “starve” tumor cells. However, this approach can exacerbate hypoxia in the tumor core, further promoting proliferation and metastasis. Recent studies have demonstrated that protocatechuic acid nanoliposomes (PCN) can inhibit tumor growth and metastasis by stabilizing vascular tight junctions and increasing pericyte coverage. This is achieved through the occupation of EPCR and the activation of PAR-1, which induces heterodimerization of PAR-1 and PAR-3. Consequently, this leads to Gα13-RhoA-mediated activation of Tie2 and stabilization of vascular structures via the AKT-FOXO3a signaling pathway.^621^

##### Formation of immune evasion

In the metabolic immunological microenvironment of tumors, M1 macrophages, which originally possess anti-tumor functions, are ‘tamed’ and transformed into M2-type TAMs that promote tumor development, becoming significant ‘accomplices’ in tumor initiation and progression. The primary function of M2-type TAMs is immune suppression, mainly through inhibiting the cytotoxic functions of T cells or inducing their exhaustion,^622^ thereby facilitating tumor immune evasion. TAMs release immunosuppressive cytokines such as TGF-β^623^ and IL-10, which not only weaken the killing ability of NK cells and induce their exhaustion^624^ but also promote the further differentiation of Th cells into Treg cells,^625,626^ suppressing anti-tumor immunity while also inhibiting the maturation and chemotaxis of dendritic cells.^627^ TAMs release a variety of chemokines, including CCL5, CCL17, CCL20, and CCL22, which alter the infiltration spectrum of T cells within the TME, resulting in reduced infiltration of CD8^+^ T cells and increased infiltration of Tregs.^628^ TAMs also release arginase Arg1, which consumes significant amounts of L-arginine in the microenvironment. L-arginine is crucial for T cell proliferation, TCR formation, and the shaping of immune memory.^629^ Some M2 TAMs also present antigens to T cells, but disruptions occur during the immunological synapse formation, leading to T cell non-responsiveness to external antigens.^630^ In the early stages of cancer development, tumor cells upregulate phagocytic signals on their surface, leading to early macrophage phagocytosis. However, fragments of apoptotic tumor cells or certain components can form DAMPs that induce inflammatory responses when phagocytosed by macrophages. Macrophages that ingest apoptotic bodies containing tumor cell DNA activate the interferon-inducible protein AIM2, thus upregulating their expression of PD-L1 and IDO^481^ and promoting the synthesis of TGF-β and IL-10 through activation of LXR and RXR by the lipid components of tumor apoptotic fragments.^480^ Although apoptotic tumor cells can induce TAM immune tolerance to some extent, avoiding inflammatory damage also promotes tumor immune evasion. However, recent studies have found that thymosin alpha-1 can reverse the M2 polarization of macrophages induced by exocytosis by activating the TLR7/SHIP1 axis, thereby reversing the immunosuppressive process.^631^

In breast cancer, TAMs synthesize large amounts of collagen by activating TGF-β signaling, promoting fibrosis. This process consumes significant amounts of arginine in the microenvironment (CD8^+^ T cell proliferation and cytotoxic activity are highly dependent on arginine metabolism). It drives the synthesis of proline and ornithine (tumor-supporting factors), severely inhibiting the killing power of CD8^+^ T cells.^554,632,633^ Pan-cancer analyses have found that apolipoprotein E (APOE^+^) TAMs in triple-negative breast cancer promote the exhaustion of CD8^+^ T cells.^634,635^ Moreover, co-culturing triple-negative breast cancer-derived cancer-associated fibroblasts (CAFs) leads to the reprogramming of blood monocytes into an immunosuppressive subset of STAB1^+^ TREM2^+^ high-lipid-associated macrophages (LAM), reducing anti-tumor immune effects.^636^ The absence of neutral ceramidase can also lead to the emergence of an immunosuppressive TREM2-related macrophage subset.^637^ On the other hand, although PARP inhibitors (PARPi) can promote TAMs to revert to an anti-tumor phenotype,^638^ the response to PARPi in BRCA1-deficient breast tumors is severely limited by pro-tumorigenic TAMs, which not only inhibit CD8^+^ T cells but also suppress tumor cell DNA damage induced by PARPi, thus inhibiting the activation of the DNA-sensing STING pathway and reducing inherent anti-tumor immune responses.^639^ In colorectal cancer, high expression of CXCL1 promotes the infiltration of TAMs, suppressing the killing power of both CD4^+^ and CD8^+^ T cells, thereby facilitating immune escape.^640^ Inhibition of CXCL1 expression can also suppress M2 polarization of TAMs.^641^ Colorectal cancer cells regulate the PTEN/AKT and SCOS1/STAT1 pathways by releasing extracellular vesicles containing miR-21-5p and miR-200a, inducing PD-L1 expression and M2 polarization in TAMs.^642^ TAMs’ released extracellular vesicles can also promote upregulation of PD-L1 in tumor cells.^643^ Pan-cancer analysis has identified that Krüppel-like factor 3 (KLF3), a key transcriptional repressor, is abnormally expressed across various tumor types, particularly in pancreatic cancer, and is closely linked to immune pathways. It is also highly expressed in TAMs.^644^ Interestingly, after cancer cells are attacked in liver cancer, they secrete prostaglandin E2 (PGE2). In addition to its inherent ability to inhibit the growth of CD8^+^ T cells, PGE2 can also induce CX3CR1^+^ TAMs to shift into a pro-tumor phenotype. These TAMs then secrete interleukin-27 (IL-27), which induces CD8^+^ T cells to upregulate the immune checkpoint molecule TIM-3 and decrease the expression of TNF-α and IFN-γ, leading to CD8^+^ T cell exhaustion and facilitating immune evasion.^645^ Furthermore, liver cancer cells activate the Smad signaling pathway through TGF-β in the microenvironment, which in turn activates the expression of the downstream molecule SOX18. SOX18 acts as a transcriptional activator to upregulate the expression of CXCL12 and PD-L1 in liver cancer cells, thereby recruiting TAMs and Tregs and suppressing the anti-tumor immune response.^646^ In hepatocellular carcinoma, integrated multi-omics analyses have revealed that three key transcription factors—ILF2, YBX1, and HMGA1—are regulated by the long non-coding RNA HCG18. ScRNA-seq shows that HCG18 co-localizes with macrophages and stem cells and is positively correlated with M0-type macrophages but negatively correlated with M1-type and M2-type macrophages. High expression levels of HCG18, ILF2, YBX1, and HMGA1 are strongly positively associated with cancer stem cells. Moreover, pan-cancer analysis indicates that high expression of HCG18 implies high sensitivity to immune checkpoint therapy.^647^ Moreover, the role of TAMs in the TME is closely related to the overall metabolic state of the body. For example, in obesity, specific inflammatory factors such as interferon-γ, tumor necrosis factor (TNF), leptin, insulin, and palmitate activate the mTORC1 pathway, enhancing macrophage glycolysis activity, thereby increasing PD-1 surface expression and suppressing T cell activity. However, high PD-1 expression on TAMs inhibits their glycolysis (a typical feature of M2 macrophages). In obese models, anti-PD-1 treatment significantly enhances the glycolysis activity of TAMs (a feature of M1 macrophages). It boosts their antigen-presenting ability and the expression of co-stimulatory signals. It suggests that while obesity promotes immune suppression in the TME, it may respond more positively to tumor immune therapy.^648,649^ TAMs’ ketone metabolism also suppresses anti-tumor immunity. In hepatocellular carcinoma, TAMs highly express 3-oxoacid CoA-transferase 1 (OXCT1), promoting the formation of ketone metabolism by-product succinate, which promotes H3K4me3 at the upstream promoter of Arg1. Transcriptionally activated Arg1 leads to CD8^+^ T cell exhaustion.^650^ Interestingly, single-cell sequencing revealed a novel subset of interstitial CD68^+^ SOX2^+^ double-positive TAMs in glioblastoma under conditions of cytomegalovirus infection, which is closely associated with poor clinical prognosis and resistance to ICB therapy. Additionally, this subset promotes the generation of immunosuppressive FXYD6^+^ T cells.^651^ Pan-cancer analysis has revealed that elevated CD93 expression is closely associated with poor prognosis and immune evasion in the majority of cancers. CD93 expression correlates strongly with the presence of monocyte/macrophage lineages and neutrophils while showing a negative correlation with Th1, MDSCs, NK cells, and T follicular helper cells in nearly all cancers. Therefore, CD93 could serve as a prognostic marker for malignant cancers.^652^ CD44 and CD147 have also been found to be abnormally expressed in various cancers and are often closely associated with resistance to immune checkpoint therapy. The specific knockout of CD44 can inhibit M2 polarization of macrophages in the TME.^653,654^ Tumor cells can also induce TAMs to highly express IL-15Rα, thereby reducing tumor cell production of CX3CL1 and lowering the recruitment of CD8^+^ T cells, which is a significant reason for the inefficacy of anti-PD-1 agents.^655^ Additionally, tumor cell oxidative stress-generated ROS suppresses the release of their miR-155-5p exosomes, thereby increasing PD-L1 expression in TAMs.^656^ TAMs also release IL-23 to promote Foxp3 expression, thereby stabilizing the immunosuppressive function of Tregs in the microenvironment.^657^ Type I IFNs can effectively reprogram TAMs into an anti-tumor phenotype, but when interferon signals coexist with M-CSF signals, they promote Arg1 expression through the STAT3 signaling pathway.^658^ Through single-cell sequencing analysis of numerous patients across multiple cancer types who did not respond to immune checkpoint blockade (ICB) therapy, a unique niche of TIM3^+^ VISTA^+^ TAMs was identified. This genetic signature is associated with negative prognostic and predictive impacts across various cancers. The interaction between HMGB1/VISTA exposed by immunogenic cell death (ICD) of cancer cells and TIM3/VISTA on TAMs inhibits the paracrine IFN response, thereby hindering the production of pro-inflammatory TAMs. This further reduces the neoantigen load, ultimately causing PD-1/PD-L1-targeted ICB therapy to function inadequately. Instead, it increases the ecological proportion of this unique TAM subset within the overall macrophage population.^659^

Current macrophage-targeted cancer immunotherapies primarily include inhibiting TAM recruitment, inducing TAM repolarization, depleting TAMs within the TME, and enhancing TAM phagocytic activity. Targeting chemokine ligands and receptors can reduce TAM recruitment. In contrast, antibody-mediated therapies targeting TAM-specific markers, selective chemotherapeutic agents that eliminate TAMs, nanoparticles (NP), and other nanotechnologies for receptor-mediated depletion can effectively mitigate TAM’s negative impact on antitumor therapies. Furthermore, TAMs can be reprogrammed to adopt an antitumor phenotype, or the CD47-SIRPα axis can be blocked to enhance TAM phagocytic activity, promoting their role in antitumor immunity.

However, despite these advancements, clinical trial outcomes may not always meet expectations, with challenges in translating preclinical success into sustained clinical efficacy. For instance, inducing TAM depletion can lead to off-target effects, such as the non-specific depletion of monocytes, which may compromise immune homeostasis beyond the TME. Therefore, selective targeting of TAMs while preserving TRMs is critical to reducing side effects and ensuring therapeutic safety and efficacy. Additionally, approaches like targeting inflammatory cytokines, blocking inhibitory receptors, and enhancing antigen presentation can provoke immune-related adverse effects, as the immune system may start attacking normal tissues. Another promising strategy is to inhibit TAM recruitment to the TME, which could enhance the effectiveness of existing immunotherapies. This method involves disrupting chemokine signaling and using monoclonal antibodies or small molecule inhibitors. However, future clinical studies must carefully consider potential resistance mechanisms and side effects, such as accelerated metastasis. Stimulating TAM phagocytic activity is another promising approach, as it can potentially destroy large populations of tumor cells, enhancing the efficacy of chemotherapy and immunotherapy. Though research on this technology is still limited, focusing on the CD47-SIRPα signaling pathway and leveraging macrophages as key players in the fight against tumor cells could yield encouraging results in future studies.

Lastly, the repolarization of TAMs towards an M1-like phenotype has garnered significant interest. While reducing M2 TAM abundance or promoting the switch from M2 to M1 could be crucial in cancer therapy, this approach faces challenges due to the poorly understood polarization mechanisms and the unpredictable nature of patients’ immune responses. Additionally, it remains difficult to fully attribute TAM’s tumor-promoting role solely to M2 macrophages, as M1 macrophages may contribute to inflammation and tumor development. The subtle distinctions between these phenotypes and the numerous factors influencing their plasticity—differing across cancer types and patients—complicate the definition of therapeutic strategies.

In conclusion, while macrophage-targeted therapies hold great promise in precision medicine, developing effective immunotherapies requires a multidisciplinary approach. This involves devising comprehensive treatments that address the complex mechanisms regulating macrophage behavior within the TME. As research advances, future strategies must integrate multiple approaches to precisely modulate TAM functions, considering both the pro- and anti-tumor effects of macrophages in cancer progression.

### Inflammation and autoimmune disease

As previously mentioned, macrophages play a critical coordinating role in the body’s physiological and pathophysiological states. These cells are not only broadly active in inflammation response, immune modulation, and tissue repair but are also increasingly recognized for their role in autoimmune diseases. Although the pathogenesis of autoimmune diseases primarily involves T cells and B cells, these cells may not fully explain all the etiologies and pathological processes of autoimmune diseases, making the role of macrophages particularly significant.^660^ In conditions such as RA, systemic lupus erythematosus (SLE), and systemic sclerosis (SSc), macrophages display high plasticity, exhibiting pro-inflammatory or anti-inflammatory properties depending on the microenvironmental conditions. For example, macrophages promote tissue healing and fibrosis in SSc by producing TGF-β and other anti-inflammatory factors. Conversely, in rheumatoid arthritis, macrophages exacerbate inflammation and tissue damage by releasing pro-inflammatory cytokines such as IL-6 and TNF-α. Additionally, the failure of macrophages to clear apoptotic cells and process self-antigens may be a contributing factor in triggering and sustaining autoimmune responses.

Despite the increasing recognition of the role of macrophages in many autoimmune diseases, their precise roles in disease progression and their specific functions and subtypes in different types of autoimmune diseases are still not fully understood. A deeper understanding of macrophages’ functions and regulatory mechanisms is crucial for developing new therapeutic strategies.

#### Rheumatoid arthritis (RA)

RA is characterized by extensive infiltration of macrophages in the synovium, with the severity of the disease positively correlated with the degree of macrophage infiltration.^661^ This phenomenon serves as a reliable biomarker for the disease.^662^ Increasing research indicates that the activation of macrophages plays a key role in the pathogenesis of RA, where excessive activation of pro-inflammatory M1 macrophages and incomplete polarization of anti-inflammatory M2 macrophages significantly exacerbate the condition. Recent studies have identified a specific type of macrophage, characterized by high expression of CX3CR1 and low expression of Ly6C, F4/80, and IA/IE, termed arthritis-associated osteoclastogenic macrophages (AtoM). These are precursors to pathogenic osteoclasts in arthritis.^37^ Local disease conditions are typically directly related to synovial macrophage production of IL-6 and TNF-α.^663–667^ IL-6 activates STAT3, which promotes the upregulation of nuclear factor κB receptor activator ligand (RANKL) in osteoblasts and enhances osteoclast differentiation, leading to joint destruction in RA.^668^ Studies show that the histone demethylase inhibitor GSK-J4, by reducing H3K27me3, decreases macrophage IL-6 expression, effectively delaying disease progression.^669^ The NOTCH signaling pathway is another crucial factor contributing to RA’s imbalance between M1 and M2 macrophages. Thus, using NOTCH inhibitors in arthritis models can effectively alleviate TNF-induced M1 polarization of macrophages,^670^ and NOTCH signaling is critical in regulating osteoclast differentiation and bone resorptive activity.^671^ ERK, a vital member of the MAPK family, also plays an important role in macrophage polarization. The adipocytokine nesfatin-1 promotes high expression of CCL2 in RA synovial fibroblasts via the ERK/MAPK pathway, enhancing M1 polarization and chemotaxis, thereby exacerbating RA conditions.^672^ Nesfatin-1 has been identified as a potential risk factor for RA.^673^ As previously mentioned, M1 macrophages primarily rely on aerobic glycolysis,^674^ while M2 macrophages depend on oxidative phosphorylation. Hypoxic conditions in the synovial microenvironment promote the transcriptional enhancement of glycolytic enzymes through HIF-α, the expression of the key pro-inflammatory cytokine IL-1β, and M1 polarization of macrophages.^503,675^ Additionally, lysine acetyltransferase 2 A (KAT2A) supports glycolytic reprogramming of macrophages by inhibiting NRF2 activity and its downstream antioxidant molecules, promoting M1 polarization and synthesis of NLRP3 and IL-1β. Specific KAT2A chemical inhibitor MB-3 significantly improves synovitis and bone destruction in a collagen-induced arthritis model.^676^ Therefore, inhibiting M1 polarization and enhancing M2 polarization can effectively alleviate inflammatory responses in RA.^677,678^ For instance, activation of SIRT1 promotes phosphorylation of AMPKα, increases M2 gene expression, and inhibits M1 gene expression, and SIRT1 transgenic mice exhibit milder arthritis symptoms.^679^ Proteins c-Fos and c-Jun, whose expression levels are elevated in RA synovial tissues, act by reducing the synthesis of Arg-1, diminishing the anti-inflammatory properties of macrophages.^680,681^ Similarly, IL-10 effectively reduces the release of pro-inflammatory cytokines by macrophages by inhibiting the NF-κB signaling pathway.^682,683^ GRK2 inhibits the migration and pro-angiogenic characteristics of Flt-1^+^ macrophages through the PPARγ signaling pathway, reducing synovitis and M1 polarization in a mouse model of RA.^684^ Recent research has found that exosomes derived from anti-inflammatory (M2) macrophages, modified on their surface with oligo-lysine and matrix metalloproteinase (MMP)-cleavable polyethylene glycol (PEG), can clear cfDNA after MMP cleavage and induce M2 polarization, significantly inhibiting RA and providing robust cartilage and bone protection.^685^ Additionally, single-cell sequencing has identified a large number of IL-1B highly expressed macrophages with enhanced NLRP3 inflammasome activity in the peripheral blood and synovial fluid of inflammatory arthritis induced by PD-1 inhibitors (PD-1-IA). Yet, this macrophage subtype is not observed in RA.^686^ Significant research potential remains concerning the heterogeneity of macrophage subtypes in RA.

In the synovial lining, synovial lining cells (SLCs), key players in RA, can be categorized into type A (macrophage-like synoviocytes, MLS) and type B (fibroblast-like synoviocytes, FLS).^687,688^ Both cell types coexist in the synovial layer, typically contributing to the production of synovial fluid and the maintenance of joint function. However, in RA, MLS secrete a plethora of pro-inflammatory cytokines, chemokines, and growth factors that not only activate FLS but also cause them to release IL-6, prostaglandin E2 (PGE2), and matrix metalloproteinases (MMPs). This cascade of reactions leads to persistent extracellular matrix degradation and exacerbates synovial inflammation.^689^ Furthermore, FLS promotes osteoclastogenesis in RA by producing fibroblast growth factor 2 (FGF2), further aggravating the disease.^690^ A newly discovered gene, Merlot, regulates the NFATc1-GSK3β axis to inhibit osteoclast differentiation and promote apoptosis, effectively alleviating the progression of RA.^691^ Studies have shown that citrullinated and malondialdehyde-acetaldehyde-modified fibrinogen, by activating macrophages to release soluble mediators like PDGF-BB, can induce FLS to transform into an invasive phenotype, worsening RA.^692^ Macrophage extracellular traps (METs) can also induce severe synovial inflammation. METs stimulate the DNA sensor cGAS in FLS, promoting proliferation, invasion, migration, and tumor-like biological behaviors and releasing many inflammatory cytokines such as TNF-α, IL-1β, MMP9, and MMP13.^693^ Moreover, FLS can also influence the phenotype of macrophages in RA. For instance, exosomes produced by FLS that contain PTX3 not only promote the expression of IL-6 and TNF-α in macrophages and induce an invasive phenotype in these cells. Interestingly, FLS can also induce macrophages to develop a non-M1/M2 polarized phenotype; inflammatory mediators like PGE2 produced by FLS can polarize macrophages toward heparin-binding EGF-like growth factor (HBEGF) polarization. HBEGF-type inflammatory macrophages, besides releasing a vast array of inflammatory mediators, can conversely prompt FLS to convert into a destructive invasive phenotype.^694^ This complex interplay highlights the critical role of cell-cell interactions within the RA synovial microenvironment, influencing disease progression and offering potential targets for therapeutic intervention.

#### Systemic lupus erythematosus (SLE)

In SLE, macrophages play a critical role in disease progression by regulating adaptive immunity. Macrophages in SLE patients exhibit an abnormally high activity level, with the degree of activation correlating directly with disease activity and potentially life-threatening conditions.^695,696^ Macrophages activate humoral immunity through the CD40/CD40L co-stimulatory molecule, promoting plasma cell differentiation, antibody secretion, and class switching, thus influencing the disease process.^697^ Compared to healthy controls, SLE patients have increased expression of CD40L on peripheral blood macrophages.^698^ Mouse experiments have shown that overexpression of CD40L can induce lupus-like autoimmune diseases, whereas neutralizing CD40L can significantly reduce autoreactive B cell activation and antibody production.^699,700^ Single-cell sequencing of SLE samples revealed that macrophages and DCs in SLE patients express higher levels of interferon-stimulated genes (ISGs) and that M1 macrophages dominate in SLE.^701^ High levels of IFN-γ and TNF-α in the serum of SLE patients promote the polarization of macrophages towards the M1 phenotype.^702^ M1 macrophages further exacerbate SLE inflammation by secreting IL-1β, IFN-γ, CXCL10, CCL2, IL-6, and TNF-α.^703–708^ IL-6, in particular, appears to enhance the activity of B cells in SLE, and inhibiting IL-6R reduces the number of circulating plasma cells and the levels of autoantibodies.^708^ Moreover, the impaired phagocytic function of macrophages^709^ and the serum of SLE patients can accelerate macrophage apoptosis, another significant factor contributing to SLE.^710,711^ This primarily manifests in the inability to clear apoptotic cells and immune complexes in a timely manner. Recent studies show that macrophages lacking Late Endosomal/Lysosomal Adaptor, MAPK And MTOR Activator 5 (LAMTOR5) suffer from lysosomal dysfunction and abnormal mTORC1 activation, leading to ineffective phagocytosis and digestion of external apoptotic cell debris.^712^ Macrophages usually bind immune complexes through Fc receptors, and the genetic diversity of Fc receptors typically determines the efficiency of immune complex clearance, which is closely associated with regional SLE prevalence and directly affects disease progression.^713^ SLE patients’ macrophages highly express pyruvate kinase M2 (PKM2), and overexpression of PKM2 enhances the activity of TLR4, TLR7, and TLR9 pathways.^714^ In SLE, impaired gut barriers lead to the release of serum LPS, which induces macrophage pyroptosis through the Caspase 11-GSDMD pathway. Notably, pyroptotic macrophages promote the differentiation of mature B cells independently of T cells.^715^ Interestingly, during differentiation, the red blood cells of SLE patients fail to switch from glycolysis to oxidative phosphorylation, thereby retaining mitochondria. These mitochondria-containing red blood cells become a new source of interferons upon being phagocytosed by macrophages.^716^

Research indicates that disulfiram can effectively alleviate inflammation caused by SLE by inhibiting gasdermin D (GSDMD) and IL-1β-mediated pyroptosis.^717^ An increase in oxysterols, especially 7α, 25-dihydroxycholesterol (7α, 25-OHC), suppresses STAT activation and the production of IFN-β, chemokines, and cytokines in macrophages when 7α, 25-OHC binds to its receptor EBI2.^718^ Additionally, miR-4512 can inhibit innate immune activation and the formation of neutrophil extracellular traps (NETs) in SLE by targeting TLR4 and CXCL2.^719^ Recent studies show that spermine, by binding to the FERM and SH2 domains of JAK1, inhibits the phosphorylation of JAK1 induced by IFN-I, IFN-II, IL-2, and IL-6, thus suppressing cytokine signaling. Treatment with spermine alleviates autoimmune lesions in SLE mice and reduces IFN-I signaling in monocytes from SLE patients.^720^ IL-4-induced M2 macrophages can effectively inhibit the development of SLE.^721,722^ Meanwhile, knockout of scavenger receptors and low levels of TGF-β are associated with poor prognosis in SLE.^723,724^ Interestingly, despite the detection of significant levels of the anti-inflammatory cytokine IL-10 in SLE patients, the levels of IL-10 unexpectedly correlate positively with disease activity.^725,726^ This may reflect a feedback mechanism where the body controls excessive inflammation by increasing IL-10 production. However, in the presence of IFN-α, the anti-inflammatory effects of IL-10 can be reversed to pro-inflammatory actions. IL-10 activates STAT1, promoting the expression of CXCL9, CXCL10, IFN-γ, and platelet-activating factor, further enhancing inflammation and M1 macrophage polarization.^727^ Therefore, inhibiting IFN-α can effectively improve the inflammatory damage caused by SLE.^728^ Overexpression of AKT2 in macrophages can interact with IRF3 and phosphorylate IRF3 at Thr207, weakening its nuclear translocation and thereby reducing the production of IFN-α, which in turn slows the progression of SLE.^729^ Research has shown that the 21-mer phosphopeptide P140 can effectively halt the progression of SLE by inhibiting the formation of NETs. However, in a localized Imiquimod (IMQ)-induced lupus model, following the administration of P140, a significant accumulation of CX3CR1-positive macrophages was observed in the lungs of lupus-prone mice, along with the development of pulmonary fibrosis. This macrophage response was associated with increased citrullinated histone H3 (H3cit) in the cytosol, the expression of interleukin-1 receptor type 1 (IL-1R1) on the cell membrane, and the production of intracellular ROS.^730^

#### Systemic sclerosis (SSc)

SSc is a complex chronic autoimmune disease characterized by a significant gender disparity, with a much higher incidence in women than in men.^731^ The progression of the disease primarily involves three core processes: extensive vascular abnormalities, immune system dysregulation, and fibrosis of the skin and internal organs.^732,733^ Decades ago, researchers first discovered high levels of CD163^+^ macrophages infiltrating SSc.^734^ Macrophages are situated at a crucial intersection of inflammation and fibrosis in SSc, playing an indispensable role. Increasing evidence demonstrates that macrophage activation, particularly M2 polarization, is crucial in the pathogenesis of SSc. M2 polarized macrophages can promote the activation of fibroblasts,^735^ releasing a large amount of factors such as TGF-β, PDGF, and CCL18, which further promote tissue fibrosis.^736^ In SSc, immune complexes in patients can induce monocytes to secrete secreted phosphoprotein 1 (SPP1),^737^ macrophage colony-stimulating factor (M-CSF), and IL-6.^738^ These factors collectively influence the disease process: SPP1 can promote the activation and migration of lung fibroblasts. At the same time, the autocrine effects of M-CSF and IL-6 drive further polarization of macrophages toward the M2 phenotype. Single-cell sequencing studies have revealed a subgroup of CD163^+^ macrophages in SSc, where SPP1^+^ macrophages accumulate in areas with higher degrees of fibrosis^739,740^ and show enhanced proliferative capabilities. Additionally, in patients with diffuse cutaneous systemic sclerosis (dcSSc), a group of macrophages expressing FCGR3A has been identified that express pro-fibrotic cytokines and chemokines such as IL-6 or CCL18, further driving the progression of the disease.^741^ Research has found that novel nanoparticle polymers of lactic acid and glycolic acid (PLG) can mitigate TGF-β induced fibrotic reactions by inhibiting the activation of MARCO^+^ macrophages.^742^ These findings highlight the critical roles of macrophages in SSc and suggest potential targets for therapeutic interventions to modulate macrophage behavior and mitigate disease symptoms.

Although initial analyses of the lungs, skin, and blood of patients with SSc suggested that macrophages primarily polarize into an M2 pro-fibrotic phenotype, recent studies indicate that macrophages in SSc exhibit a mixed M1/M2 phenotype.^743–745^ These cells express both M2 markers, such as CD204, CD163, and CD206, and M1 markers, including CD80, CD86, and TLR4.^746^ SSc predominantly shows a shift from a pro-inflammatory state in the early stages to a balanced pro-inflammatory and anti-inflammatory state in the later stages.^747,748^ ADAR1 is extensively expressed in macrophages during the early stages of bleomycin-induced SSc and plays a crucial role in developing skin and lung fibrosis. The absence of ADAR1 significantly alleviates skin and lung sclerosis by inhibiting the expression of inducible nitric oxide synthase (iNOS) and IL-1β in macrophages through weakening the NF-κB signaling pathway.^749^ Recent studies have found that downregulating the transcription factor Fli1 in macrophages might be the primary reason for mixed polarization. This leads to concurrent upregulation of the M2 characteristic CD163 and the M1 characteristic CXCL10 when Fli1 is downregulated.^750^ As previously mentioned, the M1 polarization of macrophages is driven by JAK/STAT-dependent type II IFN signaling, while M2 polarization is associated with JAK/STAT-dependent IL-4/IL-13 signaling. Therefore, using broad inhibitors of the JAK signaling pathway is effective for treating SSc. For example, ruxolitinib, which blocks both IFN and IL-4/IL-13 pathways, can reduce M1 and M2 markers and successfully prevent skin and lung fibrosis.^747^ Additionally, studies have found that depleting B cells in SSc can promote the differentiation of pro-fibrotic macrophages, thereby inhibiting tissue fibrosis.^751^

In autoimmune diseases, pro-inflammatory macrophages are often key drivers of disease progression or primary contributors to inflammatory tissue damage. Anti-macrophage therapies in these diseases focus primarily on reducing the production of abnormal pro-inflammatory cytokines derived from macrophages, inhibiting monocyte recruitment and differentiation in inflamed areas, and upregulating anti-inflammatory cytokines. For example, in treating RA, using CD64-targeted immunotoxins to selectively eliminate synovial inflammatory macrophages and inhibit M1 macrophage polarization while inducing M2 polarization has been identified as a promising drug development strategy. Additionally, macrophage-derived extracellular vesicles are considered optimal drug carriers due to their minimal toxicity and strong targeting capabilities. Research has shown that macrophage-derived microvesicle-coated poly (lactic-co-glycolic acid) (PLGA) nanoparticles encapsulating tacrolimus can significantly inhibit the progression of RA in mice, demonstrating potential as an effective biomimetic targeted therapy for RA.^752^

With the rapid advancement of single-cell sequencing technologies, macrophage classification in inflammatory diseases has evolved beyond the simple M1/M2 dichotomy, revealing a more diverse set of macrophage subtypes that better align with different disease states. This offers more potential targets for precision and personalized therapies, paving the way for future treatment strategies.

### Cardiovascular diseases

Macrophages play a crucial role in the development of cardiovascular diseases, especially in conditions such as myocardial infarction and atherosclerosis. In the heart, 8% of non-myocardial cells are macrophages. Under disease conditions, both the number of these cells and their phenotypes undergo significant changes. As key components of the innate immune system, macrophages are massively recruited to the damaged area via CC chemokine receptor type 2 (CCR2) following cardiovascular injury, becoming the predominant immune cells in the region.^753–755^ They participate in crosstalk with other cells by releasing various mediators, influencing the chemotaxis and function of other immune cells to modulate immune responses, promoting or inhibiting the formation of endothelial cells (ECs), and directly driving fibroblast activation and proliferation. Their differentiation into myofibroblasts, thus regulating fibrosis.^756,757^ During cardiac pathology, macrophages, through their diverse phenotypes and functions, significantly influence the course of the disease. For instance, macrophages contribute to myocardial repair after a myocardial infarction by clearing dead myocardial cells. In atherosclerosis, they exacerbate vascular inflammation and plaque formation by ingesting oxidized low-density lipoproteins and transforming into foam cells. Additionally, macrophages play a key role in cardiac fibrosis, promoting extracellular matrix production and cell proliferation. Cardiac fibrosis is a common pathological outcome of various cardiovascular diseases. Excessive deposition and abnormal distribution of collagen can lead to dysfunction in cardiac contraction and relaxation.^758,759^

#### Typing of macrophages in the cardiovascular system

The cardiovascular system’s macrophages primarily originate from MDM and TRM that develop from the embryonic yolk sac during early gestation.^760^ As mentioned earlier, MDM can be subdivided into pro-inflammatory M1 and anti-inflammatory M2 types.^761^ TRMs establish stable spatial and functional connections with specific tissue cells^762,763^ and proliferate in fixed tissues in adult organisms to maintain their function. Additionally, these cells have significant anti-inflammatory effects, including clearing apoptotic cells and mitochondria and inhibiting myocardial fibrosis and hypertrophy.^756^ Regarding vascular macrophages, 60% of the macrophages in the vessels of newborn mice originate from the yolk sac, but this decreases to only 20% in adults. As age increases, embryo-derived macrophages are gradually replaced.^762^ Arterial resident macrophages typically express the Lyve-1 positive marker; some also express CCR2,^764^ and they have weaker phagocytic abilities than MDMs. In newborn mice, these macrophages exhibit low MHC-II expression, but as they mature, high MHC-II expressing resident macrophages gradually dominate.^765^ Normally, arterial resident macrophages maintain their numbers mainly through local self-renewal, a process dependent on M-CSF and CX3CL1 produced by endothelial and mesenchymal cells,^753^ with only a small portion deriving from infiltrating monocytes.^766^ Regarding cardiac macrophages, heart macrophages are spindle-shaped cells in the interstitial spaces between myocytes, fibroblasts, and endothelial cells.^767^ They are closely associated with blood vessels and are significant in signaling processes.^768^ Unlike vascular resident macrophages, heart resident macrophages almost do not express CCR2; macrophages that do express CCR2 are primarily derived from monocytes.^70^ Similarly, in cardiac tissue, macrophages that originally do not express CCR2 are gradually replaced by those that do express CCR2, while the expression of MHC-II molecules also continuously increases.^195,209,769,770^ Based on MHC-II, Ly-6C, CCR2, and CD11c, cardiac macrophages can be categorized into four major types: 1. Ly-6C^−^ MHC-II^hi^ CX3CR1^hi^ CD206^int^ MerTK^+^ CD11c^low^ CCR2^−^ CD64^+^ Macrophages; 2. Ly-6C^−^ MHC-II^low^ CX3CR1^int^ CD206^hi^ MerTK^+^ CD11c^low^ CCR2^−^ CD64^+^ Macrophages; 3. Ly-6C^+^ MHC-II^hi/low^ CX3CR1^hi^ CD206^hi/int^ MerTK^+^ CD11c^low^ CCR2^−^ CD64^+^ Macrophages; 4. Ly-6C^−^ MHC-II^hi^ CX3CR1^hi^ CD206^int^ MerTK^+^ CD11^hi^ CCR2^+^ CD103^−^ CD64^+^ Macrophages. Subgroups 1 and 2 are most common under steady-state conditions.^770^

In vascular diseases, TRMs primarily perform phagocytic and immune surveillance roles, while MDMs primarily engage in inflammation and vascular remodeling. During the early stages of vascular diseases, macrophage proliferation mainly relies on the recruitment and differentiation of circulating monocytes. Among these, monocytes expressing high levels of Ly-6C predominantly differentiate into M1-type MDMs and promote vascular inflammation by releasing IL-6 and TNF-α.^763^ In cardiac diseases, circulating monocytes are chemotaxed to lesion sites via CCL2/CX3CL1 and differentiate into macrophages expressing high levels of MHC-II and CCR2. CCR2^+^ macrophages primarily exhibit pro-inflammatory characteristics similar to M1 macrophages, while CCR2^-^ macrophages that appear in the later stages of injury possess anti-inflammatory and reparative functions similar to M2 macrophages.^771^ The extent of myocardial fibrosis induced by macrophages varies across different diseases, and macrophages play a dual role in regulating fibrosis.^757,772,773^ As previously mentioned, macrophages can promote fibrosis by releasing pro-fibrotic mediators such as TGF-β, PDGF, IL-10, VEGF, and amphiregulin (AREG), which induce activation of fibroblasts via receptors like TGF-βR, EGFR, and PDGFR on their surfaces, leading to excessive collagen production. Additionally, they can secrete inhibitors of matrix-degrading enzymes, such as tissue inhibitors of metalloproteinases (TIMPs), resulting in myocardial scarring. Conversely, fibroblasts may interact with macrophages by expressing CSF-1 and secreting macrophage chemoattractants like CCL2. Fibroblasts can also support macrophage activation by providing IL-6. In the TME, macrophages can undergo partial EMT, differentiating into fibroblast-like macrophages capable of secreting collagen and remodeling the TME. A similar subtype is expected in the heart, although it has not yet been identified.^774–777^ Moreover, macrophages can secrete MMPs to degrade the ECM and promote other cells in the microenvironment to release the same, collectively balancing myocardial fibrosis. While the activation of fibroblasts by macrophages is closely associated with M2 polarization, it is generally believed that polarization towards the M2 phenotype over time can suppress inflammation, thereby reducing fibrosis.^778^ Additionally, macrophages help resolve chronic inflammation in heart diseases through phagocytic actions and secretion of anti-inflammatory mediators such as TGF-β and IL-10, thus promoting cardiac repair.^779,780^ However, the pro-inflammatory mediators released by macrophages during the phagocytosis of necrotic tissue, such as IL-1β, IL-6, and IL-23, bind to corresponding receptors on fibroblasts, inducing an increase in pro-fibrotic factor release, thereby inducing fibrosis and adverse cardiac remodeling.^776,781^

#### Atherosclerosis (AS)

Atherosclerosis (AS) is the most extensively studied arterial disease. It is a chronic arterial condition marked by inflammation and lipid accumulation within the vessel walls, leading to plaque formation. Areas of disturbed blood flow within vessels are prone to atherosclerosis. Low shear stress from varying directions and angles of blood flow can disrupt endothelial function, which promotes morphological changes in endothelial cells and allows large molecules such as low-density lipoprotein (LDL) to more easily penetrate the endothelial layer of the vessel wall. Simultaneously, LDL binds to proteoglycans in the intima, retaining it within the endothelial layer.^782,783^ The transformation of LDL into its oxidized state, oxLDL, is a critical step in the pathogenesis of atherosclerosis. oxLDL further activates endothelial cells, causing them to release adhesion molecules and chemokines (M-CSF, CCL2, and VCAM-1).^784–786^ During the formation of atherosclerosis, monocytes from the bone marrow and liver are recruited to the plaque via CCL2/CCL7-CCR2 and CX3CR1 pathways.^787^ Early in the formation process, the recruitment predominantly involves monocytes expressing high levels of Ly-6C. Once recruited to the plaque, these monocytes differentiate into macrophages.

M1 macrophages dominate in progressive atherosclerotic plaques, primarily releasing a plethora of pro-inflammatory cytokines (IL-1β, IL-6, and TNF-α) that influence the progression of atherosclerosis (AS), including plaque instability, thrombus formation, and the chronic inflammation caused by plaques.^38,788,789^ Similar to rheumatoid arthritis, M1 macrophages in AS can also promote thrombosis and plaque formation through mechanisms such as macrophage recruitment, angiogenesis, and endothelial activation mediated by the IL-1β/NLRP3 axis.^790–792^ Interestingly, the absence of macrophage mTORC2, through signaling pathways involving FOXO1 and IL-1β, can lead to atherosclerosis.^793^ Additionally, lysosomal damage induced by cholesterol crystals can promote NLRP3-mediated inflammatory responses, with cholesterol content closely related to the number of circulating monocytes.^794,795^ In THP-1-derived macrophages, TRIM64 can activate NF-κB through the ubiquitination of IκBα, creating a positive feedback loop that exacerbates inflammation and atherosclerosis in these cells.^796^ IL-6, another significant pro-inflammatory cytokine, can exacerbate chronic inflammation in AS through trans-signaling, leading to the proliferation of vascular smooth muscle cells, thrombus formation, and increased lipid accumulation in macrophages.^797^ Studies have found that the inflammasome AIM2 promotes macrophage foam cell formation by inhibiting the ABCA1 protein. TNF can induce the production of ROS and further endothelial dysfunction, promoting the formation of oxLDL.^798^ Moreover, inhibiting the Hedgehog signaling pathway can improve early atherosclerosis by promoting autophagy and reducing foam cell formation.^799^ Most M2-polarized macrophages release anti-inflammatory cytokines such as IL-10 and TGF-β, which significantly inhibit the progression of AS. IL-10 and TGF-β can also reduce or stabilize plaque through cholesterol efflux and promote collagen formation.^800–802^ However, M2 macrophages expressing CD163 can enhance angiogenesis through the HIF-α/VEGFA pathway, promoting thrombus formation and macrophage infiltration.^803,804^

In atherosclerotic plaques, macrophages ingest apolipoprotein B-containing lipoproteins (apoB-LPs) to form lipid-laden cells called foam cells.^757,786^ Foam cells express fewer inflammatory genes, primarily expressing genes involved in the uptake, processing, and efflux of lipids.^805^ Macrophages primarily use scavenger receptors such as SR-A, CD36, and lectin-like oxidized LDL receptor-1 (LOX-1) to intake circulating lipids.^806,807^ LOX-1 can promote the development of atherosclerosis (AS) inflammation by activating NF-κB and MAPK signaling pathways.^808^ Oxidized LDL (oxLDL) exacerbates AS by inhibiting the expression of KLF2 in M2 macrophages, which induces these cells to produce pro-inflammatory cytokines.^809^ Additionally, oxLDL restricts the autophagic degradation of the NLRP3 inflammasome, thereby promoting the formation of the inflammatory environment in AS.^810^ After foam cell formation, endoplasmic reticulum stress and apoptosis-related molecular pathways cause them to release MMPs extensively, expanding the necrotic core of the plaque. In the early stages of AS, macrophages react to other apoptotic cells or components, which can inhibit the formation of the necrotic core within the plaque. However, in the mid to late stages, the phagocytic capacity of macrophages significantly decreases, and their rate of apoptosis increases, leading to uncontrollable chronic inflammation and the formation and expansion of the necrotic core within the arterial intima.^811,812^ In late-stage plaques, reduced expression of Baf60a, a component of the SWI/SNF chromatin remodeling complex, leads to decreased mitochondrial integrity and increases adhesion, apoptosis, and plaque formation.^813^ The rupture of atherosclerotic plaques, leading to acute myocardial infarction and stroke, is one of the direst consequences of atherosclerosis. Plaques prone to rupture contain a large necrotic core and a thin fibrous cap. They are characterized by high MMP activity, hydrolysis of ECM proteins, dedifferentiation of VSMCs, impaired exocytosis, and chronic inflammation.^757,766^ Macrophages contribute to plaque rupture by secreting different MMPs; M1 macrophages primarily release MMP1, MMP3, and MMP10, while M2 macrophages mainly release MMP11, MMP12, and MMP15 ^814,815^。

Research has discovered that asprosin upregulates the expression of ABCA1 and ABCG1 via the p38/Elk-1 pathway, inhibiting lipid accumulation in macrophages and reducing atherosclerosis burden in apoE^-/-^ mice.^816^ Additionally, inhibiting the MCT4 on the surface of macrophages can decrease lactate efflux, thereby enhancing p300-mediated histone lactylation at H3K18la, initiating macrophage repair, and promoting the resolution of inflammation.^817^ Increasing studies suggest that activation of PPARs can effectively prevent atherosclerosis.^818,819^ PPARα can reduce the uptake of glycated LDL (glyLDL) by inhibiting lipoprotein lipase (LPL) and decrease the levels of triglyceride-rich lipoproteins (Tg-Lp) by inhibiting the apolipoprotein B48 receptor (apoB48R) pathway.^820^ PPARα also increases the expression of ABCA1, ABCG1, and SR-BI in macrophages to promote cholesterol efflux.^786^ Furthermore, PPARγ promotes the polarization of M2 macrophages and inhibits the polarization of macrophages towards the M1 phenotype.^819^

#### Myocardial Infarction (MI)

Myocardial infarction (MI) refers to the process where myocardial ischemia leads to extensive death of cardiac muscle cells. MI primarily progresses through three phases: the inflammatory phase, the anti-inflammatory phase, and the repair phase, all of which involve extensive participation of macrophages.^775^ At the onset of injury, many necrotic cardiac cells release damage-associated molecular patterns (DAMPs), which release numerous chemotactic signals via pattern recognition receptors such as TLRs. Ly6C^hi^ monocytes are recruited to the ischemic infarct area via the CCR2/CCL2 signal and differentiate into CCR2^+^ MHC-II^hi^ macrophages,^821^ playing a primary role in the inflammatory phase. Compared to CCR2^+^ TRMs, monocyte-derived CCR2^+^ macrophages possess stronger pro-inflammatory capabilities. Interestingly, tissue-resident CCR2^-^ macrophages can inhibit the recruitment of monocytes, playing a crucial role in preventing myocardial fibrosis after heart damage.^210^ CCR2^+^ macrophages have a strong phagocytic function, working alongside neutrophils to clear debris. Still, they also secrete proteases, ROS, and inflammatory cytokines such as TNF-α, IL-1, IL-6, monocyte chemoattractant protein-1 (MCP-1), and MMPs. NETs can promote the pro-inflammatory polarization of macrophages, leading to myocardial remodeling.^822^ The cytokines synthesized by macrophages during the inflammatory phase are not conducive to benign repair outcomes following myocardial infarction; they promote fibrosis. For instance, IL-1, NLRP3, IL-6, IL-34, and angiotensin II^757,823–828^ contribute to this process. IL-1α promotes myocardial tissue fibrosis and remodeling by inducing the release of IL-6, MCP-1, and connective tissue growth factor (CTGF).^829^ Compared to IL-1α, IL-1β has a dual role in myocardial tissue fibrosis. Studies have found that IL-1R1-dependent IL-1β inhibits TGF-β-induced fibroblast contractile activity and α-smooth muscle actin expression, promoting matrix metalloproteinase synthesis, thereby reducing fibrosis. On the other hand, IL-1β induces upregulation of the AT1 receptor faster than TNF-α, and their combined effects further promote extracellular matrix remodeling and fibrosis.^830,831^ LncRNA MIAT, by downregulating IL-1β and TNF-α, inhibits macrophage inflammation, but its activation by ATP-induced NLRP3 inflammasomes is inhibited.^832^ IL-34 activates the NF-κB signaling pathway to promote recruitment and polarization of macrophages after myocardial ischemia in mice and humans, and knocking out IL-34 reduces cardiac remodeling, dysfunction, and fibrosis.^824^ Additionally, the adipocyte-derived adiponectin (ADPN) via the ADPN/AdipoR2/HMGB1 axis promotes macrophage M2 polarization, reduces IL-6 release, and stabilizes mitochondrial metabolism.^833^ IL-7, by regulating macrophage infiltration and polarization, promotes apoptosis of myocardial cells, thereby exacerbating myocardial I/R injury. Anti-IL-7 antibodies affect the production of cytokines by T helper cells (Th)1 and Th2 and promote macrophage polarization towards M2, thus reducing myocardial damage.^834^ Endothelial cells produce small extracellular vesicles (EVs) containing KLF2 that alleviate mouse myocardial ischemic injury by inhibiting the recruitment of Ly6C^hi^ monocytes.^835^ The caspase recruitment domain family member 9 (CARD9) can upregulate macrophage expression of lipocalin 2 (LCN2) and MMP9, leading to myocardial cell apoptosis and adverse remodeling after MI.^836^

**Fig. 5:**
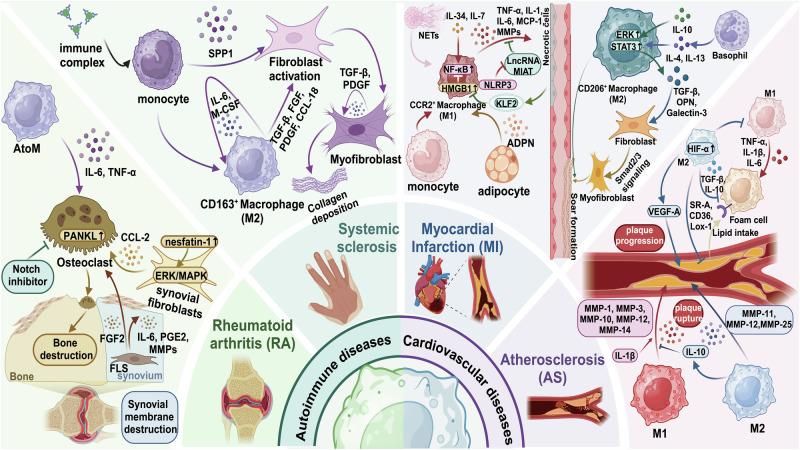
Role of Tissue Macrophages in Diseases. Macrophages play a pivotal role in developing a wide range of diseases, with distinct functions attributed to tissue-resident and monocyte-derived macrophages, as well as their M1/M2 polarization, which varies across different diseases. In autoimmune diseases such as rheumatoid arthritis and systemic sclerosis, macrophages damage tissue by releasing pro-inflammatory cytokines (e.g., IL-6 and TNF-α) and promote multi-tissue fibrosis through anti-inflammatory and fibrogenic factors. In cardiovascular diseases, including atherosclerosis and myocardial infarction, macrophage polarization facilitates phagocytosis of necrotic tissue, fibrotic repair, myocardial remodeling, and the formation and rupture of atherosclerotic plaques. AtoM arthritis-associated osteoclastogenic macrophages, IL-6 interleukin-6, TNF-α tumor necrosis factor-alpha, PANKL parathyroid hormone-related protein, CCL2 chemokine (C-C motif) ligand 2, ERK extracellular signal-regulated kinase, MAPK mitogen-activated protein kinase, FGF2 fibroblast growth factor 2, PGE2 prostaglandin E2, SPP1 secreted phosphoprotein 1, TGF-β transforming growth factor-beta, PDGF platelet-derived growth factor, CCL-18 chemokine (C-C motif) ligand 18, IL-34 interleukin-34, IL-7 interleukin-7, IL-1 interleukin-1, MCP-1 monocyte chemoattractant protein-1, NLRP3 NOD-like receptor pyrin domain-containing 3, KLF2 Krüppel-like factor 2, ADPN adiponectin, NF-κB nuclear factor kappa-light-chain-enhancer of activated B cells, HMGB1 high-mobility group box 1, IL-4 interleukin-4, IL-13 interleukin-13, OPN osteopontin, HIF-α hypoxia-inducible factor alpha

During the anti-inflammatory and repair phases (about three days after myocardial infarction), inflammation gradually subsides, and myofibroblasts proliferate to form scar tissue. Ly-6C^hi^ monocytes differentiate into reparative macrophages. This differentiation depends on Nr4a1, and the lack of Nr4a1 can lead to abnormal inflammation in macrophages, poor healing, exacerbated fibrosis, and heart failure.^837^ Reparative macrophages express high levels of CD206 and MerTK, low levels of Ly-6C, MHC-II, and CCR2, and secrete a range of anti-inflammatory and tissue fibrogenic cytokines, such as IL-10, TGF-β, HIF-α, VEGFA, and SPP1.^757,838,839^ Among these, TGF-β/Smad3 signaling is crucial as it targets fibroblasts to stimulate their migration, transdifferentiation, and the synthesis of collagen and fibronectin.^840^ IL-10, as a multifunctional anti-inflammatory cytokine, has a dual role in whether myocardial tissue undergoes fibrosis. Hypoxia-induced VSIG4 promotes the expression of IL-10 in M2 macrophages, facilitating the transformation of cardiac fibroblasts into myofibroblasts.^841^ On the other hand, IL-10 can inhibit the human antigen R (HuR)/MMP9 signaling pathway and activate STAT3 to suppress collagen deposition.^842^ Basophils can promote the conversion of Ly6C^hi^ macrophages into CD206^+^ macrophages by secreting IL-4 and IL-13, aiding in the healing of necrotic cardiac tissue.^843^ Research has shown that targeting NPM1 can reprogram the metabolism of reparative macrophages from inflammatory glycolysis to oxygen-driven mitochondrial energy production, thereby enhancing the reparative function of cardiac macrophages and promoting cardiac repair after myocardial infarction.^844^ However, other studies have found that mitochondrial metabolism influences the exocytosis of macrophages. Deficiency in mitochondrial complex I in macrophages can promote glycolysis, increase mitochondrial ROS production, exacerbate early inflammatory responses, and weaken exocytosis, thereby hindering the proliferation activation of fibroblasts and scar formation after myocardial infarction.^845^ This indicates that the metabolic mode of macrophages directly impacts the prognosis of MI. Studies have identified a group of Bhlhe41^+^ macrophages on day seven after MI. These cells can inhibit the activation of myofibroblasts, prevent excessive fibrosis, and limit infarct expansion.^846^ Additionally, sEVs derived from M2 macrophages can reduce the pro-inflammatory CCR2^+^ macrophage subgroup, which is beneficial for cardiac repair post-AMI^847^ (Fig. 5).

In the future, macrophage-targeted therapies hold great promise for treating cardiovascular diseases (CVD). Macrophages are crucial in heart injury, fibrosis, and diseases such as atherosclerosis (AS). By regulating macrophage recruitment, polarization, and function, we can effectively influence inflammation, tissue repair, and remodeling of the heart and blood vessels. Current research shows that different types of macrophages have distinct roles in various stages of cardiovascular diseases. For example, pro-inflammatory M1 macrophages are typically associated with acute inflammation and tissue damage, while anti-inflammatory M2 macrophages aid tissue repair and suppress inflammation. Future therapeutic strategies should focus on regulating these functions and account for the dynamic changes in macrophage behavior during different pathological stages.

In atherosclerosis, macrophages ingest oxidized low-density lipoproteins (oxLDL), transforming into foam cells, which promote plaque formation and inflammation. Dysfunctional foam cells exacerbate arterial hardening and increase the risk of plaque rupture, leading to acute events such as myocardial infarction (MI). Therefore, blocking the release of inflammatory mediators from macrophages or inducing the clearance of foam cells could be a potential strategy for preventing AS. In MI, macrophages play a role in the inflammatory response following injury and the repair process. CCR2^+^ macrophages are recruited during the acute inflammatory phase and exhibit pro-inflammatory effects, while CCR2^-^ macrophages help tissue remodeling during the repair phase by releasing anti-inflammatory factors and promoting fibrosis. However, excessive fibrosis can lead to further deterioration of heart function. Thus, future therapies should aim to balance macrophages’ pro-inflammatory and anti-inflammatory functions, avoiding excessive inflammation or fibrosis. Utilizing small molecules, nanoparticles, or gene therapies to regulate macrophage polarization and function precisely will be key in future cardiovascular disease treatments.^757^

Furthermore, with advances in single-cell sequencing technology, the complexity of macrophage subtypes has become increasingly apparent. Different diseases and their stages may correspond to different macrophage subtypes, offering more targets for personalized therapies. Future research should also focus on exploring emerging macrophage functions, such as macrophage extracellular traps (MET), which have shown potential therapeutic value in infections and other diseases but remain understudied in the cardiovascular field. By gaining a deeper understanding of the diverse roles of macrophages and their interactions with other immune cells, endothelial cells, and fibroblasts, future therapeutic strategies will be able to intervene in the onset and progression of cardiovascular diseases more precisely, improving treatment outcomes and minimizing adverse effects.

### Neurodegeneration disease

Over the past 15 years, numerous risk factors for neurodegenerative diseases have been identified. Among these, factors closely related to brain macrophages, such as TREM2 or APOE-positive macrophages, have been highlighted.^848,849^ These macrophage-related risk factors underscore the critical role of brain-associated macrophages/microglia in the pathogenesis of neurodegenerative diseases.^850^ This is particularly evident in Alzheimer’s Disease (AD) and Parkinson’s Disease (PD), the two most common neurodegenerative disorders. As the global population increasingly ages, the number of individuals suffering from neurodegenerative diseases such as AD and PD continues to rise. Although AD and PD represent distinct neurodegenerative pathologies, inflammatory responses play a crucial role in the progression of the pathophysiology of these diseases. These inflammatory responses can trigger toxic neuronal activation, leading to neuronal death and the formation of abnormal protein aggregates. The macrophages in the brain consist of various types, including microglia, the brain’s resident tissue macrophages originating from embryonic yolk sacs,^71^ and those located in the meninges, choroid plexus, and perivascular areas. Additionally, macrophages derived from circulating monocytes are recruited during disease or inflammatory processes. Historically, microglia have been primarily associated with neurodegenerative diseases. Recent research also finds that perivascular macrophages can participate in the pathology of Alzheimer’s disease by SPP1, which induces phagocytic states and synaptic phagocytosis in microglia, preventing synaptic loss when SPP1 expression is inhibited.^851^ Brain boundary-associated macrophages (BAMs) produce free radicals by binding to Aβ through the innate immune receptor CD36, leading to neurovascular dysfunction, cerebral amyloid angiopathy (CAA), and cognitive impairments^852^ Furthermore, anti-Aβ antibody (3D6) forms immune complexes with vascular amyloid deposits, which activate CD169^+^ perivascular macrophages, enhancing the expression of inflammatory signals and extracellular matrix remodeling genes (e.g., TIMP1 and MMP9), thereby increasing vascular permeability and recruitment of inflammatory monocytes.^853^ In PD, microglia are typically categorized into M1 and M2 phenotypes. Unlike many other diseases, in the early stages of Parkinson’s disease, M2 microglia primarily produce anti-inflammatory cytokines, alleviating neuroinflammation and promoting tissue repair. However, as the disease progresses, microglia, continually activated by stimuli such as α-synuclein, environmental toxins, or pathogens, transition to the M1 phenotype. This shift to M1 microglia exacerbates the inflammatory cascade, ultimately resulting in progressive neuronal loss.^854–856^

#### Alzheimer’s disease

Alzheimer’s disease (AD) is characterized by progressive cognitive decline and memory loss, closely linked to abnormal deposition of amyloid-β (Aβ) and pathological phosphorylation of tau protein in the brain.^857,858^ During this process, macrophages attempt to clear these pathological proteins through phagocytosis. However, their capacity is often limited as the disease progresses, potentially exacerbating inflammatory responses and neuronal damage. Moreover, studies have shown that anti-TNF-α antibodies may protect AD.^859^ The pathogenic macrophages in AD include disease-associated inflammatory (DIM) and disease-associated microglia (DAM). DIMs predominantly express various pro-inflammatory genes, such as IL-6, IL-1β, and TNF-α, which are crucial in promoting the pathological state.^860^ Conversely, DAMs primarily express genes like Spp1, Igf1, Gpnmb, and Dkk2, which are involved not only in the phagocytosis of amyloid-β aggregates but also in activating immune regulatory pathways, demonstrating their complex functionality.^39,266^ Research has found that 25-hydroxycholesterol (25-HC) can induce DAMs to produce IL-1β, leading to neuroinflammation.^861^ Dimethyl malonate (DMM), by inhibiting succinate dehydrogenase (SDH), reduces mitochondrial biogenesis in DIMs, converting them to an anti-neuroinflammatory phenotype.^862^ Despite commonalities in gene expression between DAM and DIM, particularly in Apoe and Trem2^863^ expression, their origins and functions differ significantly. DAMs primarily originate from embryonic sources and depend on the expression of the triggering receptor expressed on myeloid cells 2 (TREM2) to exert protective effects in the brain. In contrast, DIMs arise from circulating monocytes and, although they also express TREM2, do not depend on it and are typically associated with inflammatory states in the brain.^850,860^ This distinction reflects their differing pathological roles and potential therapeutic targets in AD. Peripheral MDMs can effectively reduce amyloid plaques in AD brain tissue,^864^ but the infiltration of aged macrophages can exacerbate the condition.^865^ The erythropoietin-related signaling pathway plays a significant role in halting AD progression. It enhances phagocytic activity, levels of Aβ-degrading enzymes, and the clearance rate of Aβ in peripheral macrophages. A deficiency in the EPO receptor in peripheral macrophages lowers the clearance rate of Aβ, leading to increased peripheral and cerebral levels of Aβ and accelerated progression of AD.^866^ IL-34 inhibits the differentiation of bone marrow-derived monocytes into macrophages, reducing the uptake of Aβ42 fibrils and oligomers and decreasing the expression of proteins such as CD36, TREM2, and MMP9, thereby contributing to the progression of AD.^867^ Furthermore, enhanced glycolysis-induced histone lactylation in macrophages can induce the production of β-amyloid (Aβ).^868^ Inhibiting the smad3 signaling pathway in macrophages can effectively promote the efflux of Aβ from the brain.^869^ The knockout of METTL3 in macrophages weakens m6A modification in the mRNA of DNA methyltransferase 3 A (DNMT3A), impairing the YTHDF1-mediated translation of DNMT3A. Depletion of METTL3 leads to a downregulation of ATAT1 and reduced acetylation of α-tubulin, thus enhancing macrophage migration and clearance of Aβ.^870^

#### Parkinson’s disease

Parkinson’s disease (PD) is a well-recognized neurodegenerative disorder, primarily manifesting as motor dysfunction and autonomic nervous system impairments. The neuropathology of PD is characterized by the progressive loss of dopaminergic neurons in the substantia nigra pars compacta (SNpc), and the formation of intraneuronal protein aggregates known as Lewy bodies and Lewy neurites, predominantly composed of insoluble α-synuclein.^871^ Microglia-mediated neuroinflammation plays a crucial role in the pathogenesis and progression of PD and is inversely correlated with the survival of dopaminergic neurons.^872,873^ Under normal conditions, α-synuclein exists as a soluble monomer. However, under cellular stress, misfolded α-synuclein directly contributes to neurotoxicity and activates immune cells, triggering neuroinflammatory lesions. Pathological α-synuclein activates the expression and kinase activity of LRRK2 in monocytes, inducing their recruitment to the brain. Inhibition of LRRK2 kinase may alleviate the detrimental pro-inflammatory monocyte responses in the brain.^874^ Moreover, macrophages or microglia overexpressing LRRK2 exhibit significantly reduced lysosomal degradation activity, exacerbating PD pathology.^875^ Furthermore, α-synuclein promotes the polarization of microglia to the M1 phenotype while activating NADPH oxidase, expressing pro-inflammatory cytokines and ROS, leading to sustained and progressive neurotoxicity, forming a vicious cycle.^876^ Additionally, overexpression of α-synuclein in microglia leads to phagocytic exhaustion and produces a large amount of oxidative toxicity, ultimately causing severe degeneration of DA neurons.^877^ α-Synuclein also enhances microglial glycolysis through PKM2, stabilizing the M1 phenotype and blocking mitochondrial metabolism.^878,879^ Disruptions in microglial mitochondrial fission, autophagy interruptions, and other disturbances lead to the activation of NLRP3, resulting in a more severe neuroinflammatory response.^880–882^ Inhibition of NLRP3 effectively promotes microglial polarization to the M2 phenotype and reduces the accumulation of α-synuclein.^883,884^ Research also reveals that microglia-derived exosomes can transfer misfolded α-synuclein between cells, exacerbating neuronal damage.^885–887^ Perivascular macrophages play a significant role in PD, predominantly expressing genes like CD68 and MHC-II for antigen presentation and T-cell activation.^888,889^ PAAN/MIF nuclease inhibition can prevent neurodegenerative changes in Parkinson’s disease, protecting against neurodegeneration induced by α-syn PFF, AAV-α-syn overexpression, or MPTP toxicity.^890^

The heterogeneity of macrophages in Alzheimer’s and Parkinson’s disease presents new directions for future treatments. In both neurodegenerative diseases, inflammatory responses are closely linked to neuronal damage, with macrophages playing a critical role. Studies have shown that different macrophage subtypes exhibit distinct functions at various stages of these diseases, such as disease-associated microglia (DAM) and disease inflammatory macrophages (DIM) in Alzheimer’s disease and BAM (border-associated macrophages) in Parkinson’s disease. These cells contribute to regulating inflammation, clearance of pathological proteins, and either protecting or damaging neurons through different pathways. Future research should further investigate the specific roles of these macrophage subtypes in disease progression, especially how they influence the development of the disease through molecular pathways.

Additionally, the integrity of the BBB is becoming increasingly important in Alzheimer’s and Parkinson’s diseases. It has been found that damage to the BBB may affect the function of macrophages and microglia, exacerbating inflammatory responses. Therefore, future therapeutic strategies need to focus on repairing the BBB and modulating macrophage functions when the BBB is compromised to reduce neuroinflammation and protect neurons. With the rapid advancement of single-cell sequencing technologies, new macrophage subtypes are being discovered, offering more possibilities for personalized therapies. For example, targeting specific pro-inflammatory or anti-inflammatory macrophage subtypes, along with molecular-targeted drugs, antibody treatments, or gene editing technologies, may reduce disease-related inflammation and improve patient outcomes. Research into genes like TREM2 has revealed the potential protective role of macrophages in clearing pathological protein aggregates and regulating inflammation, which could lead to innovative therapies based on these targets.

### Metabolic disorders

In current medical research, obesity and insulin resistance are recognized as key precursor states for metabolic diseases such as type 2 diabetes and non-alcoholic fatty liver disease (NAFLD). Obesity is not merely an independent health issue but also a catalyst for various metabolic disorders, particularly when it leads to insulin resistance, significantly increasing the risk of type 2 diabetes. NAFLD, which directly reflects the liver’s response to obesity and insulin resistance, is one of the most common chronic liver diseases worldwide,^891^ and is closely linked to type 2 diabetes. These metabolic conditions and diseases are often associated with chronic low-grade inflammation,^892,893^ where cytokine signaling can interfere with insulin pathways, impairing glucose uptake and uncontrolled fat breakdown, ultimately resulting in ectopic lipid storage and propagating insulin resistance in a vicious cycle.^894^

Macrophages are the most common type of immune cells in adipose tissue, both in genetic and diet-induced obesity. Studies have shown that obesity significantly increases the number of macrophages in the adipose tissue of mice; for instance, while macrophages may constitute about 5% of all adipose tissue cells in mice of normal weight, this proportion can surge to as much as 50% in obese mice.^895–898^ The classification and polarization of adipose tissue macrophages (ATMs) remain a focal point of research in metabolic immunology. Beyond the traditional M1/M2 polarization applicable across various tissues and disease models, macrophages’ unique metabolic activation (MMe) is distinguished as a distinct subtype due to the adipose tissue (AT) environment rich in lipids, glucose, and insulin. The expression of metabolic-related genes and membrane receptors such as CD36, PLIN2, and ABCA1^899,900^ characterizes this subtype. MMe polarization features an enriched expression of lipid-handling genes,^900^ and adipocytes in the AT environment also release lipid-rich exosomes, inducing MMe polarization in MDMs.^901^ Additionally, CD9^+^ adipose tissue macrophages (ATMs) and TREM2 macrophages within AT display robust lipid uptake and processing capabilities^902,903^; TREM2 macrophages belong to a subset of lipid-associated macrophages (LAM).

#### Insulin resistance and Type 2 diabetes

Insulin resistance is a key precursor to Type 2 diabetes, with most patients experiencing obesity, a primary human cause of insulin resistance.^904^ Beyond insulin resistance, Type 2 diabetes also involves impaired insulin secretion, glucose intolerance, and hyperglycemia. In healthy/lean adipose tissue (AT), alternatively, activated M2 macrophages secrete anti-inflammatory factors to maintain tissue homeostasis. Glucocorticoids can activate anti-inflammatory macrophages to prevent insulin resistance.^905^ However, in obese individuals, AT induces M1 polarization of macrophages, as previously mentioned. M1 macrophages release a series of pro-inflammatory factors (such as TNF-α, IL-6, and IL-1β) and promote further M1 macrophage infiltration into AT. Pro-inflammatory cytokines activate inflammatory signaling pathways like JNK, ERK, p38, and NF-κB, promoting insulin resistance.^41,896,906,907^ Research has found that eosinophils can prevent this obesity-induced insulin resistance.^908^ Running and calorie expenditure can promote the production of M2-like macrophages through the TRIB3-AKT pathway, alleviating insulin resistance.^909^ Additionally, THP-1 human macrophages conditioned with IL-4 (THP1-IL-4-exo) enhance insulin-dependent glucose uptake by modulating the energy metabolism of macrophages and adipocytes and improving inflammatory responses.^40^ The absence of G protein-signaling modulator 1 (GPSM1) in macrophages can inhibit the pro-inflammatory state of adipose tissue, thus preventing the occurrence of insulin resistance. Mechanistically, the lack of GPSM1 primarily promotes the transcription of TNFAIP3 via the Gαi3/cAMP/PKA/CREB axis, thus inhibiting TLR4-induced NF-κB activation in macrophages.^910^ CITED2 can limit inflammatory responses and metabolic diseases by inhibiting STAT5 activation and promoting BCL6 expression, while macrophage CITED2 promotes obesity and insulin resistance.^911^ Silencing macrophage TXNIP improves hyperuricemia-induced insulin resistance through the IRS2/AKT and NRF2/HO-1 pathways. The rapamycin target complex 1 (mTORC1), and P53 have also been shown to play key roles in obesity-induced insulin resistance. High-nutrient conditions of obesity can shift ATMs from M2 to M1 through mTORC1, exacerbating insulin resistance.^912–914^ Intermittent fasting enhances visceral adipose tissue LPS-associated macrophage inflammatory phenotype through p53-driven adipocyte apoptosis, and inhibiting p53 prevents the accumulation of lipid-associated macrophages, enhancing systemic metabolic flexibility and insulin sensitivity.^915^ The NOTCH signaling pathway is a protective factor against insulin resistance.^916^ Recent studies have shown that IgG, an aging factor, can promote insulin resistance. IgG activates macrophages through the Ras signaling pathway and causes adipose tissue fibrosis, inflammation, and insulin resistance through the TGF-β/SMAD pathway.^917^ In addition, peptidase D (PEPD) can promote a fibro-inflammatory response in macrophages and adipocytes through the EGFR signal, exacerbating insulin resistance.^918^ Interestingly, embryonic vitamin D deficiency suppresses JARID2 expression through epigenetic mechanisms and activates the MEF2/PGC-1α pathway, leading to macrophage infiltration in adipose tissue, secretion of miR106-5p, inhibition of PIK3, and downregulation of AKT signaling, causing insulin resistance.^919^

#### Non-Alcoholic Fatty Liver Disease (NAFLD)

Non-alcoholic fatty liver disease (NAFLD) encompasses a spectrum of liver conditions, including non-alcoholic fatty liver (NAFL), non-alcoholic steatohepatitis (NASH), and cirrhosis. NAFL represents a basic form of fatty liver, characterized by significant lipid accumulation in hepatocytes without substantial inflammation or damage that would impair liver cell function. It is generally considered less severe and, with proper management, may not progress to more serious liver conditions. NASH, on the other hand, not only involves lipid accumulation but also significant steatosis accompanied by inflammation and some degree of fibrosis, which can progress to cirrhosis—a critical precursor to hepatocellular carcinoma.^920–922^ KCs, lipid-associated macrophages (LAMs), and MDMs are recognized as key players in the core pathophysiological development of NAFLD.^923^ Traditionally, it has been challenging to distinguish KCs from other liver macrophages, as they share many common macrophage surface markers. However, advancements in single-cell sequencing technologies have recently identified core genes unique to KCs, including CD5L, VSIG4, CD163, FOLR2, MARCO, and SLC40A1, with specific surface markers such as VSIG4, Clec4F, and FOLR2.^77^ Liver-associated LAMs, often called biliary-LAMs, predominantly express GPNMB, SPP1, TREM2, and CD9.^77,902,924^ MDMs in the liver are typically characterized by high expression of CD11b, intermediate expression of F4/80, and receptors CCR2 and CX3CR1.^925^

In the early stages of non-alcoholic fatty liver disease (NAFLD), excessive fat accumulation in liver cells leads to steatosis. These steatotic hepatocytes can activate KCs through CCL2/CXCL10 signaling and extracellular vesicles. Additionally, free cholesterol ingested by KCs can act as a pro-inflammatory agent, further stimulating their activity.^926–928^ Activated KCs release pro-inflammatory cytokines, such as IL-1β and TNF-α, which inhibit the peroxisome proliferator-activated receptor (PPAR)-α pathway in hepatocytes, thereby impairing lipid metabolism and exacerbating hepatic steatosis.^929^ Moreover, the IL-6/STAT3 pathway can significantly increase susceptibility to NAFLD.^930^ As inflammation progresses, the presence of TIM4^+^ mature Kupffer cells diminishes, replaced by TIM4^-^ Ly6C^hi^ CCR2^+^ MDMs, also known as monocyte-derived Kupffer cells (MoKCs). MoKCs are less effective in promoting triglyceride storage in the liver, thus exacerbating liver injury.^931^ Knocking out these Ly-6C^hi^ macrophages markedly reduces liver inflammation and fibrosis.^931–934^ Research has found that macrophages deficient in STING enhance nuclear YAP activity, reduce lipid accumulation, and increase the expression of autophagy-related proteins. However, dual deficiency of STING and YAP exacerbates lipid deposition.^543^ Inhibiting the dopamine receptor D2 by selectively blocking the YAP signaling pathway in macrophages reduces steatohepatitis.^935^ The Hippo/YAP pathway also plays a significant role in hepatocytes, with YAP-expressing hepatocytes rapidly and effectively activating the expression of proteins that promote fibrosis (COL1A1, TIMP1, PDGFc, TGFβ2) and inflammation (TNF, IL-1β).^936^ Lipid-associated macrophages (LAMs) also play a significant role in promoting steatohepatitis. MS4A7 enhances TREM2^+^ macrophage-induced NLRP3 inflammasome activation through lipid droplet mediation, exacerbating steatohepatitis.^937^ Zbtb18 transcription activates FXR-mediated FAO and CLTC expression, inhibiting the activity of the NLRP3 inflammasome and thus alleviating hepatic steatosis.^938^ Research has shown that the transcription factor XBP1 can also regulate the progression of NASH; macrophages deficient in XBP1 alleviate steatohepatitis by reducing NLRP3 expression and pro-inflammatory cytokine secretion and promote their M2 polarization.^939^ Persistent obesity induces TNF and IL-1β-mediated proteolytic cleavage, leading to the shedding of TREM2 from LAMs, resulting in the loss of efferocytosis, thereby triggering chronic liver inflammation and NASH.^940^

Liver fibrosis is a critical hallmark of late-stage non-alcoholic steatohepatitis (NASH), where progressive, long-term fibrosis may lead to cirrhosis, portal hypertension, and even hepatocellular carcinoma.^941^ The complexity of liver fibrosis arises from the interplay among immune cells like macrophages, hepatic stellate cells, and myofibroblasts.^942^ The fibrotic microenvironment fosters the generation of scar-forming macrophages, which express high levels of IL-1β, SPP1, LGALS3, CCR2, and TNFSF12, contributing further to the fibrotic process.^942^ These macrophages release factors such as TGF-β, TNF, IL-1β, and galectin-3, which activate hepatic stellate cells to produce collagen and exacerbate fibrosis.^943,944^ Hepatic CYR61 polarizes infiltrating monocytes via the IRAK4/SYK/NF-κB signaling pathway, enhancing their inflammatory and fibrogenic transcriptional profiles.^945^ Additionally, XBP1 can promote fibrosis by impairing macrophage mitophagy, releasing mtDNA that activates the STING pathway in macrophages to drive fibrogenesis.^946^ Downregulation of METTL14 affects the translation efficiency mediated by YTHDF1, reducing GLS2 levels, creating an oxidative stress environment, and recruiting CX3CR1^+^ CCR2^+^ MDMs, which in turn activate hepatic stellate cells to promote fibrosis.^947^ Activation of MerTK in macrophages induces phosphorylation of AKT, STAT3, ERK1/2, p38, and upregulation of VEGF-A expression, enhancing the pro-fibrotic phenotype of human hepatic stellate cells.^948^ Mucosal-associated invariant T (MAIT) cells can promote macrophages to adopt a pro-fibrotic phenotype, thus facilitating the progression to cirrhosis.^949^

**Fig. 6:**
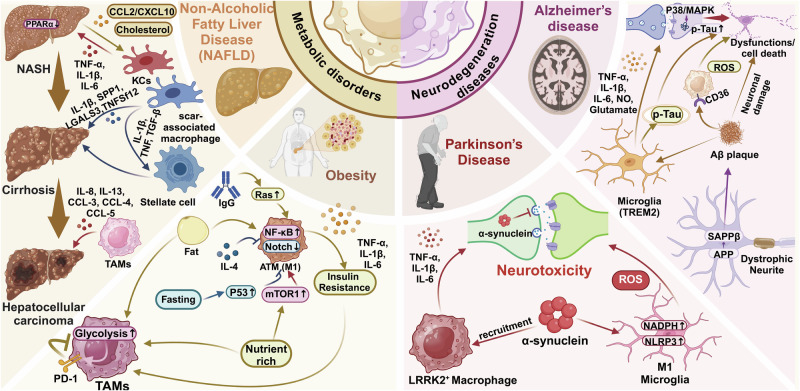
Role of Tissue Macrophages in Diseases. In neurodegenerative disorders such as Alzheimer’s and Parkinson’s diseases, macrophages are implicated in neuronal damage and neurotoxicity, involving the processing of amyloid-beta and alpha-synuclein. In metabolic disorders like obesity, which represents a state of systemic chronic inflammation, macrophages accelerate the development of insulin resistance and affect the efficacy of cell-based immunotherapies through tumor-associated macrophage interactions. Additionally, the diverse functions of macrophages are also evident in the progression of hepatic diseases, including hepatitis, liver cirrhosis, and hepatocellular carcinoma. p-Tau phosphorylated tau, APP amyloid precursor protein, SAPPβ soluble amyloid precursor protein beta, TREM2 triggering receptor expressed on myeloid cells 2, LRRK2 leucine-rich repeat kinase 2, NADPH nicotinamide adenine dinucleotide phosphate (reduced form), NOTCH NOTCH signaling pathway, mTOR1 mechanistic target of rapamycin complex 1, P53 tumor protein p53, CXCL10 C-X-C motif chemokine ligand 10, LGALS3 galectin-3 TNFsf12 tumor necrosis factor superfamily member 12

Hepatocellular carcinoma (HCC), a severe complication arising from inflammation and fibrosis in the context of NAFLD, exhibits higher levels of pro-inflammatory cytokines such as IL-8, IL-13, CCL3, CCL4, and CCL5 compared to patients with NASH, accompanied by an increase in activated monocytes in the bloodstream.^950,951^ Single-cell sequencing reveals that in HCC driven by liver fibrosis, TAMs predominantly express genes such as TREM2, GPNMB, SLC40A1, APOE, C1QA, and C1QB. Fibroblast growth factor (FGF) plays a significant role in fatty liver inflammation and fibrosis. Studies indicate that macrophage-specific knockout of FGFR1 alleviates liver inflammation induced by a high-fat diet (HFD) by inhibiting the activation of MAPKs and the TNF signaling pathway, reducing lipid deposition in hepatocytes, and preventing the activation of hepatic stellate cells.^952^ FGF21 also inhibits the transition from NASH to hepatocellular carcinoma via the hepatocyte-TLR4-IL-17A signaling pathway.^953,954^ Neuregulin 4 has been found to inhibit the development of NASH-associated HCC by limiting the proliferation of TAMs and the exhaustion of cytotoxic CD8^+^ T cells.^955^ Conversely, leptin exacerbates inflammation in NAFLD by promoting CD8^+^ T cell infiltration and mediating apoptosis of hepatocytes and macrophages.^956^ Recent research shows that fatty liver disease upregulates the expression of Rab27a in the liver, promoting the production of extracellular vesicles (EVs). These EVs enhance the activity of YAP protein in liver metastases of colorectal cancer, which, through the production of CYR61, promotes the infiltration of M2 macrophages, thereby fostering cancer cell growth and an immunosuppressive microenvironment^957^ (Fig. 6).

In the future treatment of Non-Alcoholic Fatty Liver Disease (NAFLD) and Non-Alcoholic Steatohepatitis (NASH), macrophage-targeted therapies are becoming a research focus. In recent years, as the critical role of macrophages in the progression of NAFLD and NASH has been further elucidated, therapeutic strategies such as CCR2/CCR5 dual antagonists and TREM2 inhibitors have shown promising results in preclinical and clinical trials. The heterogeneity of macrophages, especially the roles of different macrophage subtypes at various stages of disease, offers the potential for more precise targeted therapies.

Macrophage-related molecular targets, such as CD163 and TREM2, have been identified as potential biomarkers for early detection and monitoring of NAFLD progression. Moreover, adoptive cell transfer therapies targeting macrophages also show great potential in preclinical studies. With advancements in bioinformatics and single-cell sequencing technologies, researchers can more clearly analyze the transcriptomic characteristics of macrophage subtypes and their dynamic behavior in metabolic diseases. This will help better understand the interactions between macrophages and the liver microenvironment, gut, and adipose tissue, providing new targets for personalized treatment of NAFLD and NASH.

In terms of treatment strategies, in addition to traditional lifestyle interventions, drug development is shifting toward multi-targeted combination therapies. Drugs such as PPAR agonists and GLP-1 receptor agonists have shown potential in clinical trials for treating NAFLD and NASH. These drugs can reduce liver fibrosis and inflammation by targeting M2-like macrophage polarization and regulating inflammatory responses. Additionally, macrophage phagocytic activity is being explored as a potential strategy for drug delivery systems, with novel drug carriers like hard-shell microbubbles, liposomes, and polymers utilizing macrophage characteristics for more precise targeted treatments.

### Trauma

Trauma typically refers to mechanical damage inflicted on the human body, resulting in compromised tissue integrity or functional impairment. Whether due to accidental injury, surgical intervention, or other forms of physical harm, trauma initiates a complex cascade of biological responses, with macrophages playing a pivotal role. Macrophages are primarily responsible for the inflammatory response and promoting tissue repair and remodeling following trauma. Immediately post-trauma, a local inflammatory response is typically elicited. During this phase, monocyte-macrophages migrate to the damaged tissue and polarize into the M1 phenotype to clear damaged or necrotic tissue. In the later stages of tissue repair, M2 macrophages dominate at the wound site, curbing local inflammation and facilitating tissue repair. M2 macrophages activate fibroblasts by secreting cytokines such as TGF-β, which significantly enhances collagen deposition and angiogenesis, collectively fostering granulation tissue formation. As previously mentioned, M2 macrophages can be further subdivided into four subtypes: the M2a phenotype, known as alternative macrophages, which promote angiogenesis and scar formation.^36^ The M2c phenotype, called Mreg-like macrophages, plays a crucial role in tissue remodeling by phagocytosing the matrix and preventing excessive fibrosis during repair processes.^958^

#### The role of macrophages in trauma inflammation and repair

During the early stages of trauma-induced inflammation, monocytes are recruited to the wound site under the influence of chemokines. These monocytes polarize into M1 macrophages in response to PAMPs or DAMPs, triggering a robust immune-inflammatory response that helps clear damaged tissue and dead cells. However, the inflammatory response mediated by M1 macrophages can further damage the initially affected tissue, potentially leading to severe sequelae. Reprogramming M1 macrophages into M2 macrophages can effectively mitigate local inflammation and enhance tissue repair by producing anti-inflammatory cytokines, fibrogenic factors, and angiogenic factors.^36,959^ Studies have shown that aspirin-triggered resolvin-D1 (AT-RvD1) and recombinant human interleukin-10 (IL-10), released from polyethylene glycol (PEG)-based hydrogels, promote the recruitment of regenerative immune cells, including CD206^+^ macrophages (M2a/c).^960^ Recent studies have shown that following trauma/inflammatory responses, the activation of GSDMD in macrophages leads to the release of 11,12-EET, which activates the MAPK cascade pathway in muscle stem cells, thereby promoting tissue repair.^961^

In the case of traumatic spinal cord injury, microglia within the lesion core progressively diminish and are replaced by an influx of blood-derived macrophages, exhibiting both anti-inflammatory and pro-inflammatory phenotypes. Peripherally, TMEM119^+^ microglia maintain their numbers through local proliferation and predominantly display a pro-inflammatory phenotype. As time progresses post-injury, inflammation in the lesion core gradually subsides, yet significant APP^+^/e06^+^ neuronal axonal and dendritic damage is detectable in the peripheral tissue, persisting for months or years post-injury.^962^ Studies have indicated that both central and peripheral microglia/macrophages display increased mitochondria and phagosomes upon spinal injury, suggesting these cells are in stress.^963^ However, a subset of MRC1^+^ spinal macrophages can upregulate the expression of the anti-inflammatory mediator CD163, promoting IL-10 release, which mitigates microgliosis and astrogliosis, and alleviates mechanical and thermal hypersensitivity in animal models of neural injury.^964^ Additionally, the lncGBP9 sponge absorbs miR-34a to rescue SOCS3 expression, thereby exacerbating spinal injury through p-STAT1 mediated enhancement of macrophage M1 polarization.^965^ However, biocompatible hyaluronic acid and methylcellulose (HAMC) hydrogels loaded with fatty extracts (FE) can promote macrophage polarization from the inflammatory M1 to the anti-inflammatory M2 phenotype via the STAT6/Arg1 pathway, thus reducing inflammation in spinal injury models.^966^ In intervertebral disc injury models, biomimetic nanomaterials encapsulating MnO2 nanoparticles and TrkA overexpressing macrophage membranes effectively bind various inflammatory and neurotrophic factors, inhibiting inflammation-induced NPC apoptosis, matrix degradation, and neurogenesis. This also significantly reduces ROS production and M1 polarization by macrophages, effectively alleviating disc inflammation.^967^ In skin injuries, LPS increases macrophage cholesterol accumulation in a DNMT1-dependent manner, and the cholesterol content determines cellular stiffness and motility, thus affecting the effective chemotaxis of macrophages at the trauma site.^968^ Studies have shown that in lung injuries caused by mechanical overventilation, besides macrophage WISP1 and TLR4 exacerbating the damage,^969,970^ the angiotensin II type receptor AT2R can inhibit macrophage M1 polarization and alveolar macrophage apoptosis, thereby curbing further injury progression.^971^ Silencing ROCK1 reduces lung injury by inhibiting NLRP3 signaling in M1 macrophages.^972^ Additionally, the absence of YAP lowers pro-inflammatory cytokine levels (IL-1β, IL-6, and TNF-α) and enhances M2 polarization, thus mitigating lung injury.^973^ In a model of long-segment urethral trauma, researchers found that a long-lasting anti-inflammatory and antioxidant hydrogel composed of lipoic acid, small molecule glycine, and γ-Fe2O3 nanoparticles, through mechanical stimulation, achieves rapid re-epithelialization and guides macrophages towards M2 polarization, effectively reducing inflammation and repairing trauma.^974^ Radiation trauma disrupts the vascular microenvironment of HSCs, with studies revealing that post-irradiation residual CD206^+^ macrophages exhibit activated M2 polarization, and the expression of the mechanosensitive ion channel Piezo1 is upregulated in response to mechanical environmental changes induced by bone marrow ablation. Piezo1 activation can promote VEGF-A expression, thereby facilitating the recovery of sinusoidal vessels.^975^

#### Diabetic trauma

Diabetes, characterized as a chronic low-grade inflammatory condition, often results in non-healing wounds in affected individuals.^976^ Unlike the orderly progression of normal wound healing, chronic wounds fail to transition from the inflammatory to the proliferative phase, resulting in persistent, unresolved inflammation.^977^ The pathophysiology of chronic wounds is complex, influenced by multiple factors, including hyperglycemia, venous insufficiency, arterial hyperperfusion, and sustained pressure, which affect the normal function of macrophages.^36,978^ In diabetic wound tissue, the lack of transcription factors FOXM1 and STAT3 significantly reduces chemotaxis of macrophages and neutrophils,^979^ and the phagocytic ability of M1 macrophages in chronic wounds is diminished. This leads to the prolonged presence of damaged tissue and necrotic cells at the wound site, serving as harmful stimuli. The ensuing persistent inflammation promotes the transition of immune cells to an exhausted state and impedes the transition from M1 to M2 macrophages, thus hindering wound healing.^980^ Only after these dysfunctional macrophages have completely cleared pathogens and damaged tissue can the transition from M1 to M2 occur; hence, diabetic wound macrophages exhibit a “slow in, slow out” characteristic.^981^ In diabetic wounds, excessive ROS activate the NF-κB pathway, leading to transcriptional silencing of the nuclear factor erythroid 2-related factor 2 (Nrf2), thereby perpetuating a vicious cycle of oxidative stress and inflammation (Table 1).

**Table 1:** An overview of features of different tissue macrophage populations

Macrophage type	Organ/system	Origin	Main surface markers	Functions	References
Erythroblastic island macrophage	Bone marrow	HSCs-derived myeloid progenitors	F4/80^+^ EPOR^+^	Support for the proliferation, differentiation and maturation of erythroid precursor cells	^155,157^
Osteoclasts	Bone marrow,spleen, blood	Embryonically derivation fused with nuclei from HSC-derived macrophages	TRAP^+^	Bone resorption and remodeling via releasing proteolytic enzymes and acids	^81,157,164,165^
Osteal macrophages(OsteoMacs)	Bone	/	F4/80^+^ CD68^+^ CD169^+^	Regulation of osteoblasts’ activity and the bone matrix’s mineralization immune surveillance support for the hematopoiesis	^170–175^
Alveolar macrophages	Lung	Yolk sac EMPs	CD11c^+^ SiglecF^+^ CXC3R1^−^	Clearance of pulmonary surfactant phagocytosis of inhaled particles immunosurveillance	^12,130,186–189^
Lung interstitial macrophages	Lung	Monocytes in the embryonic period/adulthood	CXC3R1^+^ CD11b^+^ SiglecF^-^	Immune surveillance	^17,194^
Kupffer cells	Liver	Yolk sac EMPs	F4/80^+^ CLEC4F^+^ TIM4^+^	Clearance of cellular debris, senescent cells and pathogens from the blood promotion of immune tolerance; regulation of systemic metabolism of iron and lipids	^12,186,205^
Liver capsular macrophages	Liver	Monocytes	F4/80^+^ CX3CR1^+^ MHCII^+^	Neutrophil recruitment; immune surveillance	^201,207^
Cardiac macrophages (TIMD4 cluster)	Heart	Yolk sac and fetal liver progenitors	TIMD4^+^ LYVE1^+^ MHC-II^low^ CCR2^-^	Maintenance of the structural and functional integrity of the heart via regulating inflammation, fibrosis and tissue repair	^209–213^
Pericardial macrophages	Heart	/	Gata6^+^ or MHCII^+^	Regulation of cardiac damage and prevention of fibrosis after myocardial infarction;antigen presentation	^208,214^
Red pulp macrophages	Spleen	Yolk sac and fetal liver progenitors	F4/80^+^ VCAM1^+^ CD11b^Low^	Clearance of senescent red blood cells, platelets and other cells from the blood	^1,12,27,216^
Marginal zone metallophilic macrophages	Spleen	Adult bone marrow/blood monocytes	CD169^+^	Interaction with the antibody producing B lymphocytes and DCs	^220,221^
Marginal zone macrophages	Spleen	Adult bone marrow/blood monocytes	MARCO^+^ SIGNR-1^+^	Antigen capture	^222^
White pulp macrophages	Spleen	Adult bone marrow/blood monocytes	CD68^+^ F4/80^-^	Phagocytosis and clearance of apoptotic B cells	^130,222^
Sinusoidal macrophages	Lymph nodes	/	CD169^+^	Activation of B and T lymphocytes via delivering captured antigens to DCs	^130,223^
Lamina propria macrophages	Intestine	Mainly adult bone marrow Blood monocytes	CD64^+^ MHC-II^hi^ CD206^+^	Phagocytosis of surrounding material (such as apoptotic cells); antigens collection; support for epithelial stem cell proliferation; induction of microbiota-specific regulatory T Cells	^226–231,233^
Intestinal macrophages	Intestine	/	TIM4^+^ MHCII^+^	Maintenance of intestinal movement; support for the growth and function of neuronal bodies in the enteric nervous system as well as blood vessels	^1,227,228,235–239^
Microglia	Brain	Yolk sac EMPs	F4/80^+^ CX3CR1^+^ CD11b^+^	Immune sentinel functions, maintenance of brain homeostasis via scavenging apoptotic cells as well as regulating neurogenesis and synaptic activity	^71,131,186,240,242,259–262^
Brain perivascular macrophages	Brain	Yolk sac EMPs, monocytes	/	Regulation of CSF flow dynamics; promotion of proper dynamics of the ECM	^242,265,266^
Skeletal muscle macrophages	Skeletal muscle	Embryonic and bone marrow precursors	CD11b^+^ F4/80^+^ CD64^+^	Maintenance of tissue homeostasis; promotion of muscle growth and regeneration	^271^
Kidney-resident macrophages	Kidney	Yolk sac EMPs	CD64^+^ F4/80^+^ CD11c^+^	Monitor and clearance of macromolecules(such as circulating Immune complex); promotion of renal vascular and ureteric bud branching development	^276^
White adipose tissue macrophages	Lean white adipose tissue	Yolk sac EMPs	F4/80^+^ CD11b^+^ CD206^+^	Regulation of lipid metabolism, inflammatory responses and energy expenditure in adipose tissue	^3,12^
Langerhans cells	Skin	Yolk sac EMPs	/	Regulation of immune defense system; maintenance of skin homeostasis	^80,240,286,291,293,299–301^

In diabetic wounds, keratinocytes induce the expression of the NLRP3 gene in wound macrophages through IL-1 receptor-mediated signaling, enhancing inflammasome activation in the presence of PAMPs and DAMPs, thereby inhibiting wound healing.^982^ However, suppose the content of Nrf2 is normal in the wound. In that case, it can promote keratinocytes to secrete CCL2, which drives macrophage chemotaxis and induces their epidermal growth factor (EGF) expression, thereby stimulating keratinocyte proliferation.^983^ Recent research has developed a nanoenzyme hydrogel spray composed of oxidized alginate and methacrylic acid gelatin, which can restore macrophage Nrf2 transcriptional activity in vitro, inhibiting the production of ROS, particularly hydroxyl radicals.^984^ Interestingly, the traditional Chinese medicine formula ‘San Huang Anti-inflammatory Prescription’ activates the AMPK/NRF2 signaling pathway, downregulating the HMGB1-mediated abnormal inflammatory microenvironment and improving diabetic foot conditions.^985^ The expression of CD64 also plays a crucial role in the healing of diabetic wounds; in CD64 knockout mice, wound healing is significantly delayed, particularly on the seventh day, with a marked reduction in CD163^+^ M2 macrophages infiltrating the wounds of diabetic mice.^986^ The lncH19 expressed by fibroblasts, by inhibiting p53 activity and GDF15 release, alleviates fibroblast cell cycle arrest and increases macrophage infiltration in damaged tissue, thus promoting the healing of diabetic wounds.^987^ Interestingly, exosomes secreted by adipose tissue macrophages from healthy lean mice containing miR-222-3p promote M2 polarization of macrophages, thereby enhancing wound healing in diabetic mice.^988^ Studies show that a flexible liposome containing phosphatidylserine (PS), mimicking apoptotic cells (D-PSLs), can persistently bind to macrophage membranes, effectively promoting M2 macrophage polarization, increasing the expression of the endothelial marker CD31 and accelerating anti-inflammatory and diabetic wound healing processes.^989^ Additionally, a ROS-scavenging hydrogel formed by cross-linking polyvinyl alcohol (PVA) with a ROS-responsive linker, by reducing ROS levels and upregulating the M2 macrophage phenotype, promotes the healing of bacterial-infected diabetic wounds.^990^ Research has found that a hydrogel composite of ginseng-derived sEVs (G-sEV) and Mg^2+^ not only promotes the chemotaxis and neurogenic differentiation of mesenchymal stem cells but also encourages M1 macrophages to reprogram into an M2 phenotype that promotes angiogenesis while creating a suitable immune microenvironment for wound healing.^991^ Studies indicate that macrophages cultured to mimic the reparative functions of TAMs, termed TAMEMs, outperform the primary macrophage phenotypes (M0, M1, M2) in vitro in terms of inhibiting inflammation, promoting angiogenesis, and activating fibroblasts, effectively enhancing the healing of diabetic mouse skin wounds^992^ (Table 2).

**Table 2:** Role of tissue macrophages in diseases

Disease Category	Specific Diseases	Macrophage Subtypes	Secretory Phenotype	Biological Function	References
Cancer	tumor microenvironment	M1 macrophages (CD14, CD16/CD33, CD40, CD86, CD80, iNOS, and TLR2)	IL-1α, IL-1β, IL-6, IL-12, IL-23, CXCL9, CXCL10, CCL5, COX-2, and TNF-α	Phagocytosis of tumor cells, enhancing the function of antigen presentation, and releasing inflammatory cytokines to activate the anti-tumor efficacy of adaptive immunity	^86,560^
		M2 macrophages (M2a: CD206, CD163, and Arg-1; M2b: CD86, HLA-DR; M2c: CD163, CD206, and MerTK; M2d: TLR2, TLR4, TLR7, and TLR9)	IL-10, IL-4, TGF-β, Arg-1, PGE2, VEGF, MMPs, IDO, and glucocorticoids	Promoting tumor growth, remodeling the tumor metabolic immune microenvironment, enhancing its invasiveness and metastasis, angiogenesis, and immune evasion	^1117,1118^
		Tie2^+^ TAMs	VEGF, PDGF-β, and CECR1	Promoting the development and maturation of tumor vasculature	^594^
		SPP1^+^ TAMs	MMP9, MMP12, MMP14, MMP19, VEGF-A, collagen, IGF-1	Promoting angiogenesis and tumor growth	^615,1119^
		C1Q^+^ TAMs	IL-10, IDO	Promoting Treg recruitment, inhibiting the effector function of CD8 T cells, and promoting M2 macrophage polarization	^1120,1121^
		FCN1^+^ TAMs	Not clarified	Related to tumor-associated inflammatory functions	^1122^
		CCL18^+^ TAMs	CCL18	Promoting tumor proliferation and shaping the tumor immunosuppressive microenvironment	^1122,1123^
	glioblastoma	CD11b^+^ CD163^+^ TAMs	PTN	Promoting the growth and development of glioblastoma	^538^
		TIM3^+^ VISTA^+^ TAMs	Not clarified	hindering the production of pro-inflammatory TAMs	^659^
		Hypoxia-TAMs	ADM	Promoting tumor vascular instability, affecting the efficacy of anti-tumor drugs	^612^
		CD68^+^ SOX2^+^ TAMs	Not clarified	Shaping the immune microenvironment leads to resistance to ICB therapy	^651^
	Breast cancer	Lyve-1^+^ TAMs	PDGF-C	Expanding the perivascular stromal cell population to create an environment conducive to angiogenesis	^581^
		APOE^+^ TAMs	Not clarified	Promoting CD8^+^ T cell exhaustion	^635^
		STAB1^+^ TREM2^+^ TAMs	Not clarified	Shaping the immunosuppressive microenvironment	^636^
	Ependymoma	CCL2^+^ TAMs	IL-1β, CCL3, CCL4	Promoting inflammatory responses to inhibit tumor occurrence and development	^561^
		CD44^+^ TAMs	VEGFA	Promoting tumor angiogenesis	
Inflammation and autoimmune disease	Rheumatoid arthritis (RA)	CX3CR1^hi^ Ly6C^low^ F4/80^low^ IA/IE^low^ Macrophages (arthritis-associated osteoclastogenic macrophages)	IL-6, TNF-α	Promoting RA joint destruction	^37^
		CD11b^+^ Flt-1^+^ GRK2^+^ macrophages	VEGF	Promoting synovitis and angiogenesis	^684^
	Systemic lupus erythematosus (SLE)	CD40L^+^ Macrophages (M1)	IL-1β, IFN-γ, CXCL10, CCL2, IL-6, and TNF-α	Activating B cells, promoting antibody production, and exacerbating systemic inflammatory responses	^698^
	Systemic sclerosis (SSc)	CD163^+^ Macrophages (M2)	TGF-β, PDGF, and CCL18	Promoting skin and lung fibrosis	^734^
		SPP1^+^ Macrophages	TGF-β, PDGF		^737^
		FCGR3A^+^ Macrophages	IL-6, CCL18		^741^
		CD163^+^ CXCL10^+^ Fli^-^ (Mixed M1/M2 phenotype) Macrophages	M1 and M2-associated cytokines		^750^
Cardiovascular diseases	Myocardial Infarction (MI)	Ly-6C^−^ MHC-II^hi^ CX3CR1^hi^ CD206^int^ MerTK^+^ CD11c^low^ CCR2^−^ CD64^+^ Macrophages	Not clarified	Phagocytosis and immune surveillance	^770^
		Ly-6C^−^ MHC-II^hi^ CX3CR1^hi^ CD206^int^ MerTK^+^ CD11^chi^ CCR2^+^ CD103^−^ CD64^+^	TNF-α, IL-1, IL-6, MCP-1, and MMPs	Clearing damaged tissue but also potentially exacerbating myocardial remodeling and fibrosis	
		Ly-6C^−^ Nr4a1^+^ CD206^+^ MerTK^+^ Macrophages	IL-10, TGF-β, HIF-α, VEGFA, and SPP1	Promoting tissue repair and healing as well as fibrosis	^837^
		Bhlhe41^+^ Macrophages	Not clarified	Preventing excessive myocardial fibrosis and promoting repair	^846^
	Atherosclerosis (AS)	CCR2^+^ Macrophages (M1)	IL-1β, IL-6, TNF-α, MMP1, MMP3, and MMP10	Promoting the progression of atherosclerosis and causing plaque instabilit	^766^
		CCR2^-^ Macrophages (M2)	IL-10, TGF-β, MMP11, MMP12, MMP15	Suppressing inflammation and promoting cholesterol efflux	
		SR-A^+^ CD36^+^ LOX-1^+^ Macrophages (LAMs)	IL-1β, IL-6, TNF-α, and MMPs	Forming plaques and promoting their instability and necrosis	^785^
Neurodegeneration disease	Alzheimer’s disease	SPP1^+^ TREM2^+^ DAMs	SPP1	Participating in the phagocytosis of amyloid-beta and involved in activating immune regulatory pathways	^71^
		CD36^+^ BAMs	ROS	Causing neurovascular dysfunction, cerebral amyloid angiopathy (CAA), and cognitive impairment	^851^
		CD169^+^ DIMs	TIMP1, MMP9	Increasing inflammatory responses and extracellular matrix remodeling	^852^
	Parkinson’s Disease	CD11b^+^ TMEM119^+^ LRRK2^+^ Microglia	Not clarified	Exacerbating neuroinflammatory responses and neurotoxicity	^874^
		CD68^+^ MHC-II Microglia	TNF-α, IL-1β	Promoting antigen presentation and T cell activation	^887^
Metabolic disorders	Non-Alcoholic Fatty Liver Disease (NAFLD)	CD36^+^ PLIN2^+^ ABCA1^+^ TREM2^+^ Macrophages (ATMs)	Not clarified	Possessing strong capabilities in fat uptake and metabolism	^898,901^
		TIM4^+^ VSIG4^+^ CLE4F^+^ FOLR2^+^ Macrophages (KCs)	IL-1β, ΤNF-α	Inhibiting the PPARα pathway in hepatocytes, thereby impairing lipid metabolism and exacerbating hepatic steatosis	^77^
		TIM4^-^ Ly-6C^hi^ CCR2^+^ Macrophages (MoKCs)	IL-1β, ΤNF-α		^931^
		TREM2^+^ Macrophages (LAMs)	IL-1β, ΤNF-α		^937^
		SPP1^+^ LGALS3^+^ CCR^+^ TNFSF12^+^ Macrophages	TGF-β, TNF, IL-1β, and galectin-3	Promoting liver fibrosis and leading to cirrhosis	^942^
		TREM2^+^ GPNMB^+^ SLC40A1^+^ APOE^+^ C1QA^+^ Macrophages (TAMs)	TNF-α, IL-6, TGF-β, and MCP-1	Shaping an immunosuppressive microenvironment in liver cancer, promoting angiogenesis, and liver cancer growth	^952^
Trauma	Wound microenvironment	CD206^+^ Macrophages (M2a)	TGF-β, VEGF	Promoting wound angiogenesis and scar tissue formation	^36^
		CD163^+^ CD206^+^ MerTK^+^ Macrophages (Mreg)	TGF-β	Phagocytosing excessive matrix to prevent fibrosis	^958^
	Traumatic spinal cord injury	TMEM119^+^ Macrophages	IL-1β, IL-6, TNF-α	Causing persistent inflammation around the lesion and damage to neuronal axons and dendrites	^962^
		MRC1^+^ Macrophages	IL-10	Reducing the formation of microglia and astrocytes in neural injury	^964^

In trauma healing and the treatment of diabetic wounds, macrophages play a crucial role, with their dynamic changes during different stages of the wound determining the progression of inflammation and tissue repair. Studies have shown that the phenotypic plasticity of macrophages is essential for normal wound healing. The prolonged presence of M1 macrophages delays wound closure, while M2 macrophages help suppress inflammation and promote tissue repair. With the development of technologies such as single-cell sequencing, our understanding of the role of macrophages in chronic wounds has deepened, particularly in complex conditions like diabetic foot ulcers, where dysregulation of macrophage phenotypes may exacerbate wound healing delays.

Currently, macrophage-based therapeutic strategies can be categorized into two main approaches: pharmacological interventions and ex vivo macrophage transplantation. Pharmacological interventions aimed at modulating macrophage polarization to reduce the inflammatory effects of M1 macrophages or promote their transition to M2 have shown potential in treating chronic wounds. Additionally, the technology of gene manipulation to control macrophage phenotypes, especially through siRNA delivery, is advancing, but its clinical application requires further research. Furthermore, ex vivo activation of macrophages for transplantation has also been explored in wound treatment. Although this approach can accelerate wound healing under normal conditions, its application in diabetic wounds still faces challenges, such as phenotypic instability and interference with the wound microenvironment.

## Therapeutic targets and strategies

Macrophages play an active role in inflammation and resolution, which makes them promising targets of autoimmune diseases and tumor immunotherapy. It is important to decide the direction of macrophage intervention when it comes to cancer or non-cancer disease. For example, in cancer treatment, inhibiting pro-tumoral M2 macrophages or reprogramming them toward an pro-inflammatory M1 phenotype can be beneficial to enhance anti-tumor immune responses. Or we can choose target immune checkpoints (e.g., PD-1, CTLA-4) that may involve macrophages to enhance anti-tumor immunity. For inflammatory diseases, modulating macrophage activity is the key to reduce inflammation and promote tissue repair. In chronic inflammatory diseases (e.g., rheumatoid arthritis, inflammatory bowel disease), M1 macrophages are often overactivated, contributing to tissue damage and sustained inflammation. In such cases, inhibiting M1 macrophages or promoting an M2-like anti-inflammatory response is beneficial for reducing inflammation and promoting tissue repair. However, as M2-like macrophages are pro-fibrotic and M1-like phenotype area nti-fibrotic. In fibrotic diseases, strategies include inhibiting pro-fibrotic signals like TGF-β, reprogramming macrophages to an anti-fibrotic M1 phenotype, promoting ECM degradation, and blocking macrophage recruitment. In conclusion, current targeting strategies towards macrophages in cancer include depleting, reprograming, activating/inhibiting the recruitment, and modulation of metabolism and function. (Table 3).

**Table 3:** The clinical trials of macrophage-based agents

References	Strategy	Agent/Therapy	Target	Disease	Enrollment	Trial phase	Combination	NCT number
Abdou (2023)^1124^	CAR macrophages (CAR-M)	CT-0508	Her-2	solid tumors	18	1	Pembrolizumab	NCT04660929
Abdou (2024)^1125^	CAR macrophages (CAR-M)	CT-0525	Her-2	solid tumors	na	1	/	NCT06254807
Annunziata (2020)^1126^	CAR macrophages (CAR-M)	/	Mesothelin	Advanced Ovarian Cancer and Peritoneal Mesothelioma	11	1	/	NCT03608618
	Macrophage transplantation	/	/	Chronic anal fissure	199	3	/	NCT00507364
Chernykh (2016)^1127^	Macrophage transplantation	/	/	Stroke	13	1	/	NCT01845350
Sawitzki (2020)^1128^	Macrophage transplantation	Donor M reg (Mreg_UKR)	/	Renal failure	8	1/2	/	NCT02085629
	Macrophage transplantation	ixmyelocel-T	/	Peripheral arterial disease	86	2	/	NCT00468000
	Macrophage transplantation	ixmyelocel-T	/	Osteonecrosis	11	3	/	NCT00505219
	Macrophage transplantation	ixmyelocel-T	/	Critical limb ischemia	41	3	/	NCT01483898
Patel (2016)^1129^	Macrophage transplantation	ixmyelocel-T	/	IDCM	114	2	/	NCT01670981
Henry (2014)^1130^	Macrophage transplantation	ixmyelocel-T	/	DCM	61	2	/	NCT01020968, NCT00765518
Brana (2015)^1131^	Blocking CCL2/CCR2 axis	Carlumab (CNTO888)	CCL2	solid tumors	53	1	chemotherapy	NCT01204996
Pienta (2013)^1132^	Blocking CCL2/CCR2 axis	Carlumab (CNTO889)	CCL2	PC	46	2	/	NCT00992186
Raghu (2015)^1133^	Blocking CCL2/CCR2 axis	Carlumab (CNTO890)	CCL2	pulmonary fibrosis	126	2	/	NCT00786201
Sandhu (2013)^1134^	Blocking CCL2/CCR2 axis	Carlumab (CNTO891)	CCL2	solid tumors	44	1	/	NCT00537368
	Blocking CCL2/CCR2 axis	Bindarit	CCL2	Diabetic Nephropathy	na	2	/	NCT01109212
Colombo (2016)^1135^	Blocking CCL2/CCR2 axis	Bindarit	CCL2	coronary stent restenosis	152	2	/	NCT01269242
	Blocking CCL2/CCR2 axis	mNOX-E37 (Spiegelmer)	CCL2	Type 2 Diabetes Mellitus	na	1/2	/	NCT01085292
	Blocking CCL2/CCR2 axis	S0916 (MLN1202; plozalizumab; TAK-202)	CCR2	Bone Metastases	na	2	vedolizumab + nivolumab + ipilimumab	NCT01015560
	Blocking CCL2/CCR2 axis	S0916 (MLN1202; plozalizumab; TAK-203)	CCR2	Advanced Melanoma	na	1	vedolizumab + nivolumab + ipilimumab	NCT02723006
	Blocking CCL2/CCR2 axis	PF-4136309 (INCB8761)	CCR2	Metastatic Pancreatic Patients	na	2	nab-paclitaxel + gemcitabine	NCT02732938
Venturini (2023)^1136^	Blocking CCL2/CCR2 axis	BMS-813160	CCR2	Non-small Cell Lung Cancer (NSCLC) or Hepatocellular Carcinoma (HCC)	36	2	Neoadjuvant Nivolumab	NCT04123379
	Blocking CCL2/CCR2 axis	BMS-741672	CCR2	Insulin Resistance	na	2	/	NCT00699790
	Blocking CCL2/CCR2 axis	Cenicriviroc (CVC;TAK-652; TBR-650)	CCR2/CCR5 and HIV-1/2	HIV 1-Infected	na	3	/	NCT01092104
	Blocking CCL2/CCR2 axis	Cenicriviroc (CVC;TAK-652; TBR-651)	CCR2/CCR5 and HIV-1/2	HIV 1-Infected	na	2	Truvada or Sustiva Plus Truvada	NCT01338883
	Blocking CCL2/CCR2 axis	Cenicriviroc (CVC;TAK-652; TBR-652)	CCR2/CCR5 and HIV-1/2	COVID-19	na	1/2	/	NCT04593940
Behrens (2015)^1137^	Blocking Csf1/Csf1r axis	Otilimab (MOR103)	GM-CSF	RA	24	1/2	/	NCT01023256
Genovese (2020)^1138^	Blocking Csf1/Csf2r axis	Otilimab (MOR103)	GM-CSF	RA	39	2	MTX	NCT02799472
Buckley (2020)^1139^	Blocking Csf1/Csf3r axis	Otilimab (MOR103)	GM-CSF	RA	222	2	MTX	NCT02504671
Fleischmann (2023)^1140^	Blocking Csf1/Csf4r axis	Otilimab (MOR103)	GM-CSF	RA	1625	3	Tofacitinib	NCT03970837
Fleischmann (2023)^1140^	Blocking Csf1/Csf5r axis	Otilimab (MOR103)	GM-CSF	RA	1537	3	Tofacitinib	NCT03980483
Taylor (2023)^1141^	Blocking Csf1/Csf6r axis	Otilimab (MOR103)	GM-CSF	RA	56	3	Sarilumab	NCT04134728
	Blocking Csf1/Csf7r axis	Otilimab (MOR103)	GM-CSF	RA	na	3	csDMARD	NCT04333147
Schett (2020)^1142^	Blocking Csf1/Csf7r axis	Otilimab (MOR103)	GM-CSF	OA	44	2	/	NCT02683785
Constantinescu (2015)^1143^	Blocking Csf1/Csf7r axis	Otilimab (MOR103)	GM-CSF	MS	31	1/2	/	NCT01517282
Patel (2022)^1144^	Blocking Csf1/Csf7r axis	Otilimab (MOR103)	GM-CSF	COVID-19	1156	2	/	NCT04376684
	Blocking Csf1/Csf4r axis	Lenzilumab(KB003)	GM-CSF	RA	na	2	/	NCT00995449
Molfino (2016)^1145^	Blocking Csf1/Csf5r axis	Lenzilumab(KB003)	GM-CSF	Asthma	160	2	/	NCT01603277
Patnaik (2020)^1146^	Blocking Csf1/Csf6r axis	Lenzilumab(KB003)	GM-CSF	CMML	160	1	/	NCT02546284
Temesgen (2022)^1194^	Blocking Csf1/Csf7r axis	Lenzilumab(KB003)	GM-CSF	COVID-19	520	3	/	NCT04351152
Oluwole (2022)^1147^	Blocking Csf1/Csf7r axis	Lenzilumab(KB003)	GM-CSF	Large B-cell lymphoma	6	1/2	Axicabtagene Ciloleucel	NCT04314843
	Blocking Csf1/Csf7r axis	TJM2 (TJ003234)	GM-CSF	Healthy Adult Subjects	na	1	/	NCT03794180
	Blocking Csf1/Csf7r axis	TJM2 (TJ003234)	GM-CSF	RA	na	1	/	NCT04457856
	Blocking Csf1/Csf3r axis	TJM2 (TJ003234)	GM-CSF	COVID-19	384	1/2	/	NCT04341116
Tanaka (2018)^1148^	Blocking Csf1/Csf4r axis	Namilumab (MT203)	GM-CSF	Healthy Adult Subjects	24	1	/	NCT02354599
Huizinga (2017)^1149^	Blocking Csf1/Csf5r axis	Namilumab (MT203)	GM-CSF	RA	24	1	/	NCT01317797
Taylor (2019)^1150^	Blocking Csf1/Csf6r axis	Namilumab (MT203)	GM-CSF	RA	108	2	MTX	NCT02379091
	Blocking Csf1/Csf7r axis	Namilumab (MT203)	GM-CSF	RA	na	2	MTX	NCT02393378
Papp (2019)^1151^	Blocking Csf1/Csf7r axis	Namilumab (MT203)	GM-CSF	Psoriasis	122	2	/	NCT02129777
Worth (2024)^1027^	Blocking Csf1/Csf7r axis	Namilumab (MT203)	GM-CSF	Axial Spondyloarthritis	60	2	/	NCT03622658
	Blocking Csf1/Csf7r axis	Gimsilumab (KIN-1901)	GM-CSF	Ankylosing Spondylitis	na	1	/	NCT04205851
Criner (2022)^1026^	Blocking Csf1/Csf3r axis	Gimsilumab (KIN-1901)	GM-CSF	COVID-19	225	2	/	NCT04351243
Kivitz (2016)^1152^	Blocking Csf1/Csf3r axis	Gimsilumab (KIN-1901)	GM-CSF	RA	51	1	/	NCT01357759
Burmester (2011)^1153^	Blocking Csf1/Csf4r axis	Mavrilimumab (CAM-3001)	GM-CSFR	RA	32	1	/	NCT00771420
Burmester (2017)^1154^	Blocking Csf1/Csf5r axis	Mavrilimumab (CAM-3001)	GM-CSFR	RA	326	2	/	NCT01706926
Weinblatt (2018)^1022^	Blocking Csf1/Csf6r axis	Mavrilimumab (CAM-3001)	GM-CSFR	RA	138	2	/	NCT01715896
Burmester (2018)^1023^	Blocking Csf1/Csf7r axis	Mavrilimumab (CAM-3001)	GM-CSFR	RA	442	2	/	NCT01712399
Burmester (2013)^1195^	Blocking Csf1/Csf7r axis	Mavrilimumab (CAM-3001)	GM-CSFR	RA	239	2	/	NCT01050998
Cid (2022)^1155^	Blocking Csf1/Csf7r axis	Mavrilimumab (CAM-3001)	GM-CSFR	Giant cell arteritis	42	2	/	NCT03827018
Cremer (2021)^1024^	Blocking Csf1/Csf7r axis	Mavrilimumab (CAM-3001)	GM-CSFR	COVID-19	40	2	/	NCT04399980, NCT04463004, and NCT04492514
	Blocking Csf1/Csf7r axis	Mavrilimumab (CAM-3001)	GM-CSFR	COVID-19	na	2	/	NCT04397497
	Blocking Csf1/Csf7r axis	Mavrilimumab (CAM-3001)	GM-CSFR	COVID-19	na	2/3	/	NCT04447469
Kuemmel (2022)^1156^	Blocking Csf1/Csf3r axis	Lacnotuzumab (MCS110)	CSF1	BC	34	2	Carboplatin +Gemcitabine	NCT02435680
	Blocking Csf1/Csf4r axis	Lacnotuzumab (MCS110)	CSF1	Pigmented villonodular synovitis	36	2	/	NCT01643850
	Blocking Csf1/Csf5r axis	Lacnotuzumab (MCS110)	CSF1	GC	na	2	PDR001	NCT03694977
Tap (2019)^1157^	Blocking Csf1/Csf6r axis	Pexidartinib (PLX-3397)	CSF1R	Tenosynovial giant cell tumor	174	3	/	NCT02371369
	Blocking Csf1/Csf7r axis	Pexidartinib (PLX-3397)	CSF1R	PC	na	1	/	NCT02472275
Manji (2021)^1158^	Blocking Csf1/Csf7r axis	Pexidartinib (PLX-3397)	CSF1R	Sarcoma	18	1	Sirolimus	NCT02584647
	Blocking Csf1/Csf7r axis	Pexidartinib (PLX-3397)	CSF1R	BC	na	1/2	Eribulin	NCT01596751
Cassier (2019)^1159^	Blocking Csf1/Csf7r axis	Pexidartinib (PLX-3397)	CSF1R	Pancreatic or colorectal cancers	19	1	Durvalumab	NCT02777710
Lee (2020)^1160^	Blocking Csf1/Csf3r axis	Pexidartinib (PLX-3397)	CSF1R	solid tumors	11	1	/	NCT02734433
Rosenbaum (2019)^1161^	Blocking Csf1/Csf4r axis	Pexidartinib (PLX-3397)	CSF1R	Gastrointestinal stromal tumor	2	1	MEK162	NCT03158103
Lin (2020)^1162^	Blocking Csf1/Csf5r axis	BLZ945	CSF1R	solid tumors	146	1	PDR001	NCT02829723
	Blocking Csf1/Csf6r axis	ARRY-382	CSF1R	Metastatic cancer	na	1	/	NCT01316822
Johnson (2022)^1163^	Blocking Csf1/Csf7r axis	ARRY-382	CSF1R	solid tumors	76	2	Pembrolizumab	NCT02880371
Siddiqui (2021)^1164^	Blocking Csf1/Csf7r axis	Edicotinib (JNJ-40346527)	CSF1R	PC	25	1	/	NCT03177460
Dowlati (2021)^1165^	Blocking Csf1/Csf7r axis	IMC-CS4(LY3022855)	CSF1R	solid tumors	52	1	/	NCT01346358
Autio (2019)^1166^	Blocking Csf1/Csf7r axis	IMC-CS4(LY3022855)	CSF1R	BC, PC	34	1	/	NCT02265536
Falchook (2021)^1167^	Blocking Csf1/Csf3r axis	IMC-CS4(LY3022855)	CSF1R	solid tumors	72	1	Durvalumab, tremelimumab	NCT02718911
	Blocking Csf1/Csf4r axis	IMC-CS4(LY3022855)	CSF1R	Melanoma	na	1/2	Vemurafenib cobimetinib	NCT03101254
	Blocking Csf1/Csf5r axis	IMC-CS4(LY3022855)	CSF1R	Pancreatic ductal adenocarcinoma	na	1	Cyclophosphamide, pembrolizumab, GVAX	NCT03153410
Sankhala (2017)^1168^	Blocking Csf1/Csf6r axis	Cabiralizumab (FPA008)	CSF1R	Tenosynovial giant cell tumor	22	2	/	NCT02471716
	Blocking Csf1/Csf7r axis	Cabiralizumab (FPA008)	CSF1R	lymphoma	na	2	Nivolumab	NCT03927105
Weiss (2021-1)^1053^	Blocking Csf1/Csf7r axis	Cabiralizumab (FPA008)	CSF1R	Melanoma, NSCLC, renal cell carcinoma	26	1	Nivolumab	NCT03502330
Davis (2023)^1169^	Blocking Csf1/Csf7r axis	Cabiralizumab (FPA008)	CSF1R	BC	50	1/2	Nivolumab	NCT04331067
	Blocking Csf1/Csf7r axis	Cabiralizumab (FPA008)	CSF1R	Advanced tumors	na	1	Nivolumab	NCT03158272
Wang-Gillam (2019)^1170^	Blocking Csf1/Csf3r axis	Cabiralizumab (FPA008)	CSF1R	solid tumors	40	1	Nivolumab	NCT02526017
Gomez-Roca (2022)^1171^	Blocking Csf1/Csf4r axis	Emactuzumab (RO5509554)	CSF1R	solid tumors	221	1	Atezolizumab	NCT02323191
Machiels (2020)^1172^	Blocking Csf1/Csf5r axis	Emactuzumab (RO5509554)	CSF1R	solid tumors	37	1	RO7009789	NCT02760797
Gomez-Roca (2019)^1173^	Blocking Csf1/Csf6r axis	Emactuzumab (RO5509554)	CSF1R	solid tumors	99	1	Paclitaxel	NCT01494688
Rahim (2023)^1174^	Blocking Csf1/Csf7r axis	Emactuzumab (RO5509554)	CSF1R	HNSCC	10	2	Atezolizumab	NCT03708224
Ko (2023)^1175^	Blocking Csf1/Csf7r axis	Emactuzumab (RO5509554)	CSF1R	Pancreatic ductal adenocarcinoma or GC	108	1/2	Atezolizumab	NCT03193190
Hong (2021)^1176^	Blocking Csf1/Csf7r axis	TPX-0022	CSF1R	solid tumors	52	1	/	NCT03993873
Rosenbaum (2021)^1177^	Blocking Csf1/Csf7r axis	DCC-3014	CSF1R	Sarcoma	13	1	Avelumab	NCT04242238
Gelderblom (2024)^1178^	Blocking Csf1/Csf7r axis	DCC-3014	CSF1R	Advanced tumors	69	1/2	/	NCT03069469
Choi (2023)^1179^	Blocking Csf1/Csf7r axis	Q702	CSF1R	solid tumors	22	1	/	NCT04648254
	Blocking Csf1/Csf7r axis	SNDX-6532	CSF1R	solid tumors	na	1	Durvalumab	NCT03238027
Baretti (2023)^1180^	Blocking Csf1/Csf7r axis	SNDX-6532	CSF1R	Bile duct cancer	5	2	Durvalumab	NCT04301778
Patel (2021)^1181^	Blocking CD47-SIRPα axis	TTI-622	SIRPα	lymphoma or myeloma	42	1	Rituximab, PD-1 inhibitor, proteasome-inhibitor regimen	NCT03530683
Strati (2021)^1182^	Blocking CD47-SIRPα axis	CC-95251	SIRPα	solid and hematologic tumors	18	1	/	NCT03783403
Champiat (2021)^1183^	Blocking CD47-SIRPα axis	BI 765063 (OSE-172)	SIRPα	solid tumors	50	1	BI 754091	NCT03990233
Narkhede (2023)^1184^	Blocking CD47-SIRPα axis	FSI-189	SIRPα	NHL	9	1	/	NCT04502706
Daver (2023)^1097^	Blocking CD47-SIRPα axis	Magrolimab (Hu5F9-G4)	CD47	AML	87	1	/	NCT03248479
Sikic (2019)^1185^	Blocking CD47-SIRPα axis	Magrolimab (Hu5F9-G4)	CD47	solid tumors	62	1	/	NCT02216409
Brierley (2019)^1186^	Blocking CD47-SIRPα axis	Magrolimab (Hu5F9-G4)	CD47	Acute myeloid leukemia, myelodysplastic syndrome	19	1	/	NCT02678338
Roschewski (2023)^1187^	Blocking CD47-SIRPα axis	Magrolimab (Hu5F9-G4)	CD47	relapsed/Refractory Diffuse Large B-Cell Lymphoma	17	1	Acalabrutinib	NCT03527147
	Blocking CD47-SIRPα axis	Magrolimab (Hu5F9-G4)	CD47	B-cell Malignancies	na	1	Obinutuzumab and Venetoclax	NCT04599634
Fisher (2020)^1188^	Blocking CD47-SIRPα axis	Magrolimab (Hu5F9-G4)	CD47	colorectal tumors	78	1	Cetuximab	NCT02953782
Lakhani (2020)^1189^	Blocking CD47-SIRPα axis	Magrolimab (Hu5F9-G4)	CD47	solid tumor	34	1	Avelumab	NCT03558139
	Blocking CD47-SIRPα axis	Magrolimab (Hu5F9-G4)	CD47	T-Cell Lymphoma	na	1/2	mogamulizumab	NCT04541017
	Blocking CD47-SIRPα axis	Magrolimab (Hu5F9-G4)	CD47	AML	13	1	Atezolizumab	NCT03922477
Daver (2022)^1190^	Blocking CD47-SIRPα axis	Magrolimab (Hu5F9-G4)	CD47	Acute Myeloid Leukemia	38	1/2	Azacitidine (AZA) with Venetoclax (VEN)	NCT04435691
Drakaki (2020)^1191^	Blocking CD47-SIRPα axis	Magrolimab (Hu5F9-G4)	CD47	urothelial carcinoma	130-305	1/2	2 L cancer immunotherapy (CIT) combination	NCT03869190
Maakaron (2022)^1192^	Blocking CD47-SIRPα axis	Magrolimab (Hu5F9-G4)	CD47	DLBCL	33	1/2	Rituximab	NCT02953509
Ansell (2021)^1047^	Blocking CD47-SIRPα axis	TTI-621	CD47	Hematologic Malignancies	164	1	Rituximab/nivolumab	NCT02663518
Ko (2022)^1054^	Activating CD40-CD40L pathway	Sotigalimab (APX005M)	CD40	Esophageal and Gastroesophageal Junction Cancers	34	2	Paclitaxel	NCT03165994
Weiss (2021-2)^1196^	Activating CD40-CD40L pathway	Sotigalimab (APX005M)	CD40	NSCLC or metastatic melanoma	10	1/2	Nivolumab	NCT03123783
	Activating CD40-CD40L pathway	Sotigalimab (APX005M)	CD40	Advanced Pancreatic Cancer or Colorectal Cancer	na	1	Imiquimod	NCT02600949
Barlesi (2020)^1055^	Activating CD40-CD40L pathway	Selicrelumab	CD40	Advanced/metastatic solid tumors	140	1	Atezolizumab	NCT02304393
	Activating CD40-CD40L pathway	Selicrelumab	CD40	Metastatic Solid Tumors	94	1	Vanucizumab or bevacizumab	NCT02665416
Ko (2023)^1175^	Activating CD40-CD40L pathway	Selicrelumab	CD40	Pancreatic ductal adenocarcinoma or GC	108	1/2	Atezolizumab + Chemotherapy	NCT03193190
Yardley (2019)^1193^	Activating CD40-CD40L pathway	Selicrelumab	CD40	Metastatic or Locally Advanced Breast Cancer	na	1/2	Atezolizumab	NCT03424005

*AE* adverse events, *RA* Rheumatoid Arthritis, *MS* Multiple Sclerosis, *CMML* Chronic myelomonocytic leukemia, *MTX* Methotrexate, *BC* Breast cancer, *GC* Gastric cancer, *NHL* non-Hodgkin lymphoma, *PC* prostate cancer, *HNSCC* Head and neck squamous cell carcinoma, *IDCM* Ischemic Dilated Cardiomyopathy

Besides medicine intervention, clinical trials of adoptive cell therapy are under rapid development. Using macrophages to bring cytokines (e.g., IFNα, IL-12) to the tumor site and consequently activate an immune response were investigated.^993,994^ Compared to traditional drug-delivery ways, the various delivery systems (cell, viral, and nanoparticle-based) provide more possibility for those with drug resistance or low permeability of BBB. Through such delivery systems, peptide, DNA, miRNA, circRNA, and etc. can be administrated as key modulators of macrophage differentiation and polarization, including TAMs.^995,996^

### Macrophages-based targeted drugs (FDA-approved drugs and clinical trials)

Csf-1/Csf-1r axis is a signaling pathway crucial for the development and maturation of macrophages. After deletion of CSF-1R, the number of macrophages is significantly reduced, which has been studied in the clinic based on preclinical data showing a delay in tumor outgrowth in different tumor models.^997–999^ CSF-1R is primarily targeted for inhibition in therapeutic settings because CSF-1R signaling promotes the survival, proliferation, and differentiation of TAMs. CSF-1R blockade reduced the ability to unleash the immune-stimulatory capacity of TAMs with a skewing of MHC IIlow to MHC IIhi macrophages. Therefore, multiple clinical trials have been conducted with different antibodies and small molecules targeting CSF-1R, alone or in combination with standard treatment or immunotherapy. The small molecule drugs under clinical development and research mainly include Pexidartinib (PLX3397), JNJ-40346527, and BLZ945.

Preclinical studies have demonstrated that Pexidartinib significantly inhibits the growth and metastasis of osteosarcoma in mouse xenograft models.^1000^ Phase I and II clinical trials indicate that Pexidartinib has anti-tumor effects in patients with advanced tenosynovial giant cell tumor, which has been approved by FDA^997,1001,1002^; however, in patients with recurrent glioblastoma, the drug showed tolerability but did not significantly improve 6-month progression-free survival.^1003^ JNJ-40346527 has shown significant efficacy in treating relapsed or refractory Hodgkin lymphoma.^1004^ BLZ945, when combined with insulin-like growth factor receptor (IGF1R) inhibitors and phosphoinositide 3-kinase (PI3K) inhibitors, can reprogram TAMs from a pro-tumoral to an anti-tumoral phenotype and inhibit glioma progression,^1005^ with clinical trials targeting advanced solid tumors underway.

Emactuzumab (RG7155), a monoclonal antibody (mAb) targeting CSF-1R, has also entered clinical trials. Emactuzumab is a humanized mAb that binds to CSF-1R and prevents its dimerization. Preclinical data show that emactuzumab can significantly reduce the number of CSF-1R^+^ CD163^+^ macrophages and increase T cell infiltration in the TME.^997^ Results from a phase I clinical trial for tenosynovial giant cell tumor revealed that emactuzumab treatment had no dose-limiting toxicity, with common side effects being facial edema, weakness, and pruritus. During the dose-expansion phase of the trial, 24 out of 28 patients had objective responses, and 2 achieved complete responses.^1006^

However, attempts to target TAMs with Csf-1r-inhibiting therapies have met with disappointing clinical results. On the one hand, there is a restriction in the dosage that long-term loss of macrophages can cause an imbalance in body homeostasis. On the other hand, a small but discernible macrophage with high CD163 expression was found to impede the responses to T cell-based immunotherapy, which could be a potential therapeutic target.^1007^ The CD163^hi^ M2 macrophages highly express several tumor-promoting macrophage markers and have a functional anti-inflammatory transcriptome profile, but the Csf-1r expression is low.

Another treatment strategy involves using bisphosphonates to selectively eliminate TAMs, with clodronate being the most widely used.^1008^ Clodronate is encapsulated in liposomes. When macrophages phagocytose these liposomes, lysosomal phosphatases dissolve them, gradually releasing the clodronate. Once accumulated in the macrophages sufficiently, clodronate induces macrophage apoptosis, leading to their clearance. Studies have shown that this therapy can significantly reduce macrophage infiltration and limit metastasis growth in models of lung cancer bone metastasis,^1009^ breast cancer metastasis,^1010^ and mouse melanoma.^1011^ In an RCT involving 1069 primary operable breast cancer patients, clodronate significantly reduced the occurrence of bone metastases.^473^ Moreover, clodronate combined with chemotherapy drugs such as cisplatin and sorafenib exhibits synergistic anti-tumor effects in treating various tumor types.^1012,1013^ However, at the clinical level, the results of clodronate treatment in different cancers have been inconsistent, indicating that optimization of combination treatment regimens or extended clinical trials may be necessary.^473^

Immunotherapies usually require a more activated immune environment. GM-CSF treatment can regulate the proliferation and/or activation of myeloid cells to enhance immune responses, which has diverse strategies. For example, direct administration of GM-CSF is often used in combination with other therapies, especially the monoclonal antibodies, nivolumab (Opdivo) and pembrolizumab (Keytruda) in the clinic and clinical development.^1014,1015^

Sargramostim (tradename Leukine) is a GM-CSF that functions as an immunostimulatory, mainly used in combination with other cancer immunotherapy or as vaccine adjuvant. Up to now, chemotherapy + sargramostim in the treatment of Acute Myelogenous Leukemia and sargramostim after autologous and allogenic BMT in Non-Hodgkin’s lymphoma (NHL), acute lymphoblastic leukemia (ALL) and Hodgkin’s disease are approved by FDA.^1016^ Similar to Sargramostim, Molgramostim is a recombinant GM-CSF that acts as an immunostimulatory. The addition of molgramostim to antibiotic therapy can also reverse sepsis-associated immunosuppression decreases the rate of infectious complications in sepsis.^1017–1020^

GM-CSF as a vaccine adjuvant can be beneficial. Still, it depends on the specific context and goals of the vaccine, as adding it may induce side effects and overstimulation of the immune system. In a phase 1 trial of a DNA vaccine (pTVG-AR, MVI-118) in prostate cancer, persistent IFNγ immune responses were observed irrespective of GM-CSF adjuvant.^1021^ In a phase 2 trial of a DNA vaccine (pTVG-HP) and nivolumab in prostate cancer, among 14 patients to whom GM-CSF was added, three (21%) developed any subsequent PSA decline.^1014^ From this, we conclude that while GM-CSF may have provided a modest improvement for a few patients, it was not required as an adjuvant.

However, GM-CSF also plays a more pronounced role in autoimmune diseases.GM-CSF inhibitors (e.g., mavrilimumab) are being explored in clinical trials for conditions like rheumatoid arthritis and COVID-19-induced cytokine storms.^1022–1024^ Until now, other GM-CSF inhibitors, like Gimsilumab (KIN-1901) and Namilumab (MT203) have not shown significant suppression of key inflammatory pathways and therapeutic effects compared to placebo.^1025–1027^

The increase of TAMs in tumor tissues is primarily driven by the recruitment of monocytes via the CCL2-CCR2 signaling axis. CCL2 is a potent chemoattractant for monocytes, T cells, and NK cells. Studies in mouse models have demonstrated the crucial role of CCL2 and other chemokines in TAM recruitment.^1028^ Tumor cells release CCL2, which attracts cells expressing the CCR2 receptor to the tumor site. Inhibition of CCL2 signaling has been shown to suppress tumor growth and metastasis in various experimental models.^1029–1033^ However, concerns have arisen regarding the long-term efficacy of this approach. In mouse breast cancer models, discontinuation of anti-CCL2 treatment led to accelerated lung metastasis and increased monocyte recruitment, resulting in the death of the mice.^1034,1035^ Moreover, as several neutralizing antibodies targeting CCL2 have entered clinical trials, none of these studies have reported a sufficient therapeutic effect by inhibiting the CCL2/CCR2 axis as monotherapy.^1036–1038^

Among the leading candidates are carlumab (CNTO888), a monoclonal antibody against CCL2, and PF-4136309 (INCB8761), a small molecule inhibitor targeting CCR2. Carlumab, a human immunoglobulin G1κ (IgG1κ) antibody, has been shown to inhibit TAM recruitment and angiogenesis in prostate and ovarian cancer mouse models, thereby suppressing tumor growth and enhancing the efficacy of chemotherapy.^1029,1039,1040^ In a phase Ib clinical trial for advanced pancreatic cancer, the combination of PF-04136309 with FOLFIRINOX (a regimen including oxaliplatin, irinotecan, folinic acid, and fluorouracil) showed improved therapeutic outcomes compared to FOLFIRINOX alone.^1041^ However, when combined with nab-paclitaxel and gemcitabine in a phase 1b trial of previously untreated metastatic pancreatic ductal adenocarcinoma (mPDAC), PF-04136309 showed synergistic pulmonary toxicity and no significantly better clinical responses above nab-paclitaxel and gemcitabine.^1042^

CD47-SIRPα axis inhibits the accumulation of myosin IIA at phagocytic synapses and inhibits the phagocytic function of macrophages, which prevents autoimmunity. However, tumor cells also highly express CD47, achieving immune escape and promoting tumor invasion and metastasis.^1043^ Experiments in mouse transplant tumor models have shown that inhibiting CD47 can promote macrophage phagocytosis of tumor cells and is an effective tumor treatment strategy.^1044,1045^

Several antibodies and small molecule inhibitors targeting CD47 or SIRPα have been tested in clinical trials, for example, Hu5F9, CC-90002, and TTI-621. Magrolimab (Hu5F9-G4) showed promising safety and therapeutic effects in preclinical studies of human AML and pediatric brain tumors. In a recent phase 1b clinical trial including 87 previously untreated AML patients, the median OS was 9.8 months for TP53-mutant patients and 18.9 months for wild-type patients.^1046^ TTI-621, a fully human recombinant protein that blocks the CD47-SIRPα axis, has enhanced macrophage-mediated phagocytosis of tumor cells in aggressive AML and B-cell lymphoma. In a first-in-human phase I study involving 164 patients with relapsed or refractory hematologic malignancies, an overall response rate (ORR) of 13% was observed.^1047^

Some apoptosis-resistant mechanisms in cancer cells along with the hypoxia of TME can limit the effectiveness of photodynamic therapy (PDT). To address this challenge, He et al. developed an innovative biomimetic nanoplatform that integrates oxygen-enhanced PDT, ferroptosis induction, and CD47-SIRPα blockade. This system ensures precise delivery of chlorin e6 (Ce6) as a photosensitizer, hemin, and PEP20, a CD47 inhibitory peptide. In preclinical trials, mice bearing primary breast tumors exhibited significantly reduced tumor growth and lung metastasis as a result of this approach.^1048^ Moreover, hybrid nanovesicles (hEL-RS17), which are functionalized with the RS17 peptide—an antitumor agent that blocks CD47-SIRPα signaling—were shown to actively target tumor cells and modulate TAM phenotypes to enhance tumor infiltration. When used in combination with the chemotherapeutic agent shikonin, photosensitizer IR820, and immunomodulator polymetformin, hEL-RS17 exhibited superior antitumor efficacy in both 4T1 breast cancer and B16F10 melanoma models.^1049^ Another research shows that genetically engineered cell-membrane-coated magnetic nanoparticles (gCM-MNs) can disable both CD47-SIRPα and CSF-1 mechanism of tumorigenic M2 phenotype, which significantly prolonged overall mouse survival.^1050^

However, the expression of CD47 in a wide range of cells poses a challenge, as CD47 monoclonal antibodies can bind to red blood cells, causing severe anemia. QPCTL (isoQC) offers a targeted approach by modifying CD47 on tumor cells. QPCTL is a pyroglutaminase that facilitates CD47-SIRPα interaction, thus inhibiting macrophage-mediated phagocytosis of tumor cells. In tumor cells not expressing QPCTL, there is reduced pyroglutamination in CD47- SIRPα binding and enhanced macrophage phagocytosis of tumor cells.^1051,1052^ Phase 1 or 2a studies evaluating QPCT or QPCTL inhibitors for neurodegenerative diseases reported no significant toxicity. The development of QPCTL inhibitors, whether used alone or combined with CD47-blocking antibodies, is a promising area for future research.

The CD40 pathway plays a critical role in macrophage-targeting therapy, particularly in immunotherapy and cancer treatment. CD40 is a costimulatory receptor expressed on the surface of macrophages, DCs, and B cells. Its activation, through binding with its ligand CD40L (CD154) expressed on T cells, leads to a series of immune responses that are crucial for activating both innate and adaptive immunity. Engagement of the CD40 pathway significantly increases the production of pro-inflammatory cytokines like TNF-α, IL-12, and IL-6, which are crucial in driving the anti-tumor immune response and promoting inflammation. In TAMs, it promotes the transition from an immunosuppressive M2-like phenotype to a more inflammatory and anti-tumor M1-like phenotype.^473^ Trials of CD40 agonists on macrophages, such as Sotigalimab (APX005M) have reported good tolerance with chemotherapy and immunotherapy.^1053,1054^ And another CD40 agonist, Selicrelumab, In solid tumors, Selicrelumab showed promising effect, especially when combined with immune checkpoint inhibitors.^1055^ However, CD40 activation can lead to a heightened immune response, which could result in cytokine release syndrome. Managing such side effects is a key area of focus.

These molecular targets (Csf-1/Csf-1r, CCL2-CCR2, CD47-SIRPα, CD40-CD40L, etc.) are at the forefront of macrophage-targeting therapy development. They play critical roles in modulating macrophage behavior, either by reprogramming macrophages in the TME, controlling inflammation, or reducing fibrosis. Therapeutics focusing on these hotspots are actively being investigated in clinical trials for a wide range of diseases, including cancer, autoimmune conditions, and chronic inflammatory diseases(Table 3).

### Macrophages-based immunotherapies (FDA-approved drugs and clinical trials)

Due to the critical role macrophages play in tissue repair and inflammation, macrophage-based cell therapies have been utilized in the treatment of various diseases. Current strategies are mainly divided into three: (1) Ex vivo educated or generated macrophages utilizing their innate properties; (2) macrophages as delivery vehicles for small molecules, plasmid DNA, and other therapies; (3) genetically engineered macrophages enhanced for therapeutic benefits, like chimeric antigen receptor-macrophage (CAR-M). Ex vivo educated cells have the most extensive history in macrophage-based therapies. This approach is grounded in the observation of elevated monocyte and macrophage recruitment to tumors in vivo, as well as animal studies that highlight the cytotoxic capabilities of primary macrophages treated with IFNγ.^1056–1058^ Among current completed and ongoing trials of macrophage cell therapies, most are regarding the adoptive transfer of macrophages and ex vivo polarization with different strategies, while some are CAR-M trials.

Ixmyelocel-T is a multicellular therapy designed to treat ischemic conditions. It is composed of three key cell types derived from a patient’s bone marrow: mesenchymal stem cells (MSCs), M2-like macrophages, and hematopoietic cells. These cells work together to promote tissue repair, reduce inflammation, and stimulate angiogenesis. Ixmyelocel-T has been tested in several clinical trials for its potential to treat ischemic cardiovascular conditions. Ixmyelocel-T has shown positive outcomes, improving amputation-free survival and wound healing in critical limb ischemia patients. Clinical studies have demonstrated that Ixmyelocel-T can improve heart function and reduce the occurrence of major adverse cardiovascular events (MACE), slowing the progression of heart failure. In a phase IIA clinical trial (open label), heart failure patients receiving Ixmyelocel-T cells via intramyocardial delivery exhibited improved functional, clinical and quality-of-life outcomes.^1059^ Ixmyelocel-T has been explored as a treatment to acute myocardial infarction. After a heart attack, the heart tissue is damaged by a lack of blood flow. Ixmyelocel-T has been tested to aid in the repair of this damaged tissue. The ixCELL-DCM trial, a Phase IIB study, has completed recruiting patients with end-stage heart failure due to ischemic dilated cardiomyopathy, with 66 patients receiving Ixmyelocel-T and 60 receiving a placebo. This trial aims to assess the efficacy, tolerability, and safety of Ixmyelocel-T compared to placebo, with the cells administered via catheter-based, transendocardial injections. Initial 12-month results indicate that Ixmyelocel-T significantly reduces clinical cardiac events and improves patient outcomes, even though there were no notable changes in left ventricular ejection fraction (LVEF), left ventricular volumes, or six-minute walk distance (6MWD). These early findings suggest that while Ixmyelocel-T may not drastically change certain heart function measurements, it offers meaningful clinical benefits for patients with ischemic heart failure.^1060^ By modifying macrophages to express CARs that target specific tumor antigens, tumor cells expressing CAR molecular ligands can be specifically targeted and directly phagocytosed. Such macrophages are called CAR-macrophages (CAR-M). CAR-M therapies are primarily in preclinical and clinical development, with ongoing trials assessing their safety, feasibility, and efficacy in cancer patients.

A study in mouse tumor models embedded CAR molecules targeting HER2 in macrophages and evaluated the tumor-killing effect of CAR-M.^67^ The results demonstrated that in a human ovarian cancer xenograft mouse model, tail vein injection of CAR-M significantly inhibited tumor growth and prolonged the overall survival of tumor-bearing mice. Additionally, CAR-M reduced the rate of lung metastasis from ovarian cancer cells. The study also found that CAR-M, besides its inherent anti-tumor M1-like phenotype, could convert M2-like TAMs in the TME into an M1-like phenotype and promote T-cell infiltration in tumor tissues. HER2 CAR-M and CD47 CAR-M were generated for target antigen-positive ovarian cancer. Their antigen-specific phagocytosis of ovarian cancer cells was preliminarily verified in vitro and in vivo.^1061^ Preclinical studies of CAR-M have reported promising results in anti-PSCA against pancreatic cancer.^1062^ The phase 1, first-in-human (FIH) study of the anti-HER2 CAR-M, CT-0508 (NCT04660929), and CT-0525 (NCT06254807) in subjects with HER2 overexpressing solid tumors is ongoing. Preliminary results of CT-0508 showed safety, tolerability, CT-0508 infiltration, and immune activation in 7 participants with breast, esophageal, cholangiocarcinoma, ovarian, and parotid gland cancers.

iPSCs-derived, CAR-expressing macrophage cells (CAR-iMac) were generated and tested for tumor treatment.^68^ CAR-iMacs have high yield and purity and exhibit macrophage gene expression profiles, phagocytosis, polarization, and other functions. When co-cultured with CD19-positive lymphoma cells and ovarian cancer cells expressing mesothelin antigen, CAR-iMacs exhibited antigen-dependent phagocytosis and killing functions and displayed an M1-like macrophage phenotype. CAR-iMacs also demonstrated the ability to inhibit tumor growth in mouse models of hematological and solid tumors. However, there is a major hurdle of unstable CAR gene expression in hiPSCs. To solve it, another team used an elongation factor short promoter and the ubiquitous chromatin opening CBX3 element to reduce epigenetic methylation and set up a scalable differentiation system to generate CAR-iMacs that efficiently eradicate CD19-positive leukemia.^1063^ In mouse models, the CD19-CAR-iMacs effectively eradicated CD19-positive leukemia cells, significantly inhibiting tumor growth and improving survival rates of the treated mice. Preclinical studies of CAR-iMacs reported potent anticancer activity against GBM cells.^1064^

CAR-M can be combined with other therapies, such as checkpoint inhibitors, to enhance overall treatment efficacy.

### Nanoparticle-based therapy

Nano-drug delivery system (NDDS) is a drug delivery method developed by using nanotechnology, which encapsulates drugs in nano-carriers and delivers them to specific cells or tissues in a targeted and controlled manner.^1065,1066^ NDDS could deliver drugs to target sites and simultaneously improve the stability and bioavailability of drugs, thereby improving drug treatment’s efficacy and reducing side effects.^1065,1067^ Notably, specifically edited or modified NDDS could penetrate biological barriers, including the BBB, making it difficult to treat certain areas for traditional drugs to reach.^1068,1069^ In clinical and preclinical studies, it has been found that multi-drug combination therapy could reduce the incidence of adverse events and achieve more therapeutic benefits compared to single-drug therapy.^1070–1072^ NDDS may provide a new way or chance for multi-drug therapy of diseases.

Considering the different fractions in which macrophages are involved, macrophage-associated NDDS could be developed based on two main strategies.^1073–1075^ One is macrophage-targeted NDDS. It could specifically target macrophages by nanoparticles modified with specific molecules capable of recognizing receptors/ligands on macrophage surfaces, thereby affecting the pathological processes of macrophages and blocking disease progression.^1073,1074^ The other is NDDS based on macrophages or their secreted extracellular vesicles (EVs). It utilizes one of the following components: living macrophages, macrophage membranes, EVs secreted by macrophages or membranes of EVs to phagocytize or coat nanoparticles, then deliver them to specific tissue sites and help with their functions based on the properties of macrophages or their secreted EVs.^1050,1065,1074,1076,1077^

CSF-1 could affect the proliferation of CSF-1R-expressing cells and promote M2 polarization of macrophages.^761,1078^ pIL-12 + PLX@cR-PssPD could disintegrate at the tumor site and release CSF-1R inhibitor PLX3397 (PLX) and pIL-12. The synchronous inhibition of CSF-1R and the expression of IL-12 ameliorated the TME by stimulating the activation and proliferation of T lymphocytes, reducing myeloid-derived suppressor cells (MDSCs), as well as promoting the repolarization of TAMs and the maturation of DCs, thereby efficiently suppressing tumor growth and metastasis of melanoma and colon cancer.^1079^ Sun et al. developed a nanoparticle that could achieve specific delivery to M2 macrophages by the sialic acid-CD169 axis, and it has been reported to reprogram TAM due to the downregulation of CSF-1R via delivering CSF-1R siRNA in the prostate cancer model.^1080^

CCR2-MM@PLGA/Cur, a CCL2/CCR2 axis targeted multifunctional biomimetic nanoplatform, features an engineered macrophage membrane (MM) coating with increased CCR2 expression and a therapeutic drug-loaded PLGA nanoparticle core. Overexpression of CCR2 on the MM enhanced the delivery of targeted drug to the injury site and reduced macrophage infiltration, pro-inflammatory polarization of microglia, and neuronal apoptosis by sequestering CCL2. It promoted neural regeneration and motor function recovery in mice with spinal cord injury (SCI), providing a comprehensive treatment strategy for SCI.^1081^ Two-dimensional Mg2Si nanosheet(MSN) is developed as a high biocompatibility nanomaterial with super-persistent hydrogen release, and the incorporation of MSN into the chitosan/hyaluronic acid hydrogel (MSN@CS/HA) is designed as a dressing for the repair of deeply burned skin.^1082^ This hydrogel dressing could continuously generate hydrogen molecules for the support of long-term and abundant hydrogen supply, which assists with the wound repair through diverse mechanisms.Specifically, hydrogen molecules promote anti-inflammatory M2 macrophage polarization via increasing CCL2 expression to facilitate angiogenesis and reduce fibrosis.Besides, it locally scavenges overexpressed ROS, leading to the enhanced proliferation and migration capacity of skin cells.^1082^

A nanogel B^BLZ-945^@PAC-PTX offers a “dominolike” barrier elimination strategy in tumors, which promotes immunocyte infiltration for potential immune responses. Deeper spatial penetration of shrunk nanogel (PAC-PTX) could block cell membrane CXCR4,thus decreasing the recruitment of immunosuppressive cell,as well as internalize into tumor cells for tumor-killing and T-cell priming. Once it reaches the tumor site, BLZ-945 conjugated albumin (B^BLZ-945^) could be released by B^BLZ-945^@PAC-PTX under the trigger of adenosine triphosphate (ATP) and then deplete TAMs.^1083^ An engineering CXCL12 biomimetic decoy-integrated versatile immunosuppressive nanoparticle (VIN) for treating ischemic stroke has been reported. The shell of VIN (mesenchymal stem cell membrane overexpressing CXCR4) could facilitate the delivery of nanoparticles to the cerebral ischemic lesions as well as adsorb and neutralize CXCL12 for the inhibition of infiltration of peripheral neutrophils and mononuclear macrophages. Besides, the cGAS inhibitor A151 released from the VIN could inhibit the cGAS-STING pathway in microglia, leading to an M2-type polarization of microglia.^1084^

Kühne et al. designed an efficient NDDS carrying the class I/IIa selective histone deacetylase (HDAC) inhibitor valproic acid (VPA), denoted as the cellulose-based sulfated VPA-coupled (CV-S) NPs. CV-S NPs could release VPA to suppress the TLR4-MyD88-NF-κB signaling axis, attenuating the LPS-mediated inflammatory response in human primary macrophages via reducing the expression and secretion of TNF-α.^1085^ A biomimetic polymer magnetic nanocarrier, PLGA-ION-R837 @ M (PIR @ M), was developed to target TAMs by coating LPS-treated macrophage membranes on the surface. It encapsulated Fe3O4 NPs and TLR7 agonist imiquimod (R837), which could synergistically repolarize TAMs from M2 to antitumor M1 phenotype.^1086^ Resiquimod, also known as R848, is a TLR7/8 agonist shown to enhance cancer immunotherapy by promoting the transformation of M2 macrophages into M1.^1087,1088^ In vitro studies have shown that cathepsin B (CTSB)-responsive programmed brain-targeted delivery system (D&R-HM-MCA) could go through the BBB of glioblastoma (GBM) with high endocytosis efficiency and promote the repolarization of M2 macrophages into M1 type by the delivery of resiquimod.^1089^

CD47 is a glycoprotein highly expressed in many types of tumor cells, and the Inhibition of CD47 binding to SIRPɑ on macrophages could promote the repolarization of TAMs from M2 to M1.^1090^ Zhou et al. developed a BBB-penetrating nano-capsule named NAcp@CD47 to deliver anti-CD47 antibodies and STING agonists. Both in vivo and in vitro studies have shown that NACP@CD47 could improve tumor immunosuppression by promoting phagocytosis of macrophages/microglia and increasing M1-type polarization of TAMs.^1091^ Guo et al. use a layered double hydroxide nanosheet carrier to deliver a CD47 inhibitor RRX-001 and a T-type calcium channel inhibitor TTA-Q6 into lung tumors. TTA-Q6 is released in the tumor’s acidic environment. It induces calreticulin transfer to the cell surface, which could activate macrophages and mature DC, leading to the activation of anti-tumor T cells by promoting antigen presentation. Furthermore, RRX-001 reduced CD47 protein levels to prevent the immune escape of calreticulin-rich cancer cells by enhancing phagocytosis of tumor cells by macrophages.^1092^ In osteosarcoma (OS), RRX-001 and a sonosensitizer IR780 were combined with PEG-PCL nano micelles and OS cell membranes to assemble a biomimetic nano-drug, MPIRx. This nano drug could direct the migration of macrophages into tumor cells, facilitate M1-type polarization, and enhance the phagocytosis of macrophages towards OS cells.^1093^

Gao et al. developed a DNA nanocarrier. When delivered locally, the RP-182 peptide in the nanocarrier could target macrophages and repolarize M2-type TAMs into M1-type.^1094^ Specifically, the RP-182 peptide could activate phagocytosis and autophagy in M2 macrophages by activating CD206, restoring these cells to M1-type and further enhancing their abilities to engulf tumor cells and assist with intratumoral CD8^+^ T cells in tumor antigen recognition.^1095^ Subsequently, the nanoparticles could introduce ErbB2-targeted CAR into macrophages, programmed to be CAR-macrophages (CAR-M). CAR-M could engulf tumor cells and recruit T cells, NK cells, and APCs, especially DCs, leading to a wide range of adaptive immune responses.^1094^

Wang et al. developed a biodegradable NDDS named siCD40/NPs to deliver CD40 siRNA (siCD40) into HSCs, myeloid progenitors, mature DCs, and macrophages. The inhibition of CD40 in HSCs and myeloid progenitors could prevent their differentiation towards DCs and macrophages. Besides, siCD40/NPs could reduce the amounts of peripheral DCs and macrophages as well as their abilities to activate alloreactive T cells, which suppresses alloimmune responses and prolongs allograft survival in mouse models of skin allotransplantation.^1096^ For improving the treatment of colon cancer, Ding et al. developed a liposome-based nano-drug named LIC, which encapsulates phosphoinositide 3-kinase gamma (PI3Kγ) inhibitor IPI-549 and photosensitizer chlorin e6 (Ce6). In tumor, LIC-mediated immunogenic photodynamic therapy(PDT) synergistically work with MDSCs-targeting immunotherapy, which could promote the DC maturation and CD8 + T cell infiltration, simultaneously reduce the infiltration of MDSCs, immunosuppressive Tregs, and M2-type TAMs, resulting in the inhibition of tumor growth.^1097^ Song et al. designed an albumin nanoparticle named Nano-PI. It contained the immunomodulators IPI-549 and paclitaxel (PTX), which could promote the repolarization of M2 macrophages to M1 type.^1098^

L-arginine (L-Arg) and an inhibitor of Arg-1 L-norvaline (L-Nor) were loaded in a multifunctional nanoplatform named HN-HFPA. This nanoplatform could deplete intracellular GSH, produce ROS under light irradiation and release L-Arg for the NO generation after the degradation of hyaluronic acid and GSH-mediated disintegration, leading to the tumor immunogenic cell death (ICD). Besides, the L-Nor could suppress the overexpression of Arg-1 in M2 macrophages, which interfered with the balance of arginine metabolism and reversed the immunosuppressive tumor microenvironment (ITM) via increasing the ratios of M1 macrophages and CD8^+^ T cells, finally resulting in the enhanced antitumor immune responses and tumor metastasis inhibition.^1099^

Gao et al. developed a mannosylated nanotrinity containing imatinib, which could effectively induce in situ macrophage remodeling through a dual approach: activating M1-type polarization while simultaneously inhibiting M2-type polarization. This two-pronged strategy successfully combated the infection of Candida albicans.^1100^ Guanabenz (GBZ)-loaded lipoaspirate nanoparticles (Lipo-NPs) could reduce TLR4-induced inflammatory cytokines secretion by macrophages, providing a promising approach for treating inflammatory diseases.^1101^ Shi et al. designed a prodrug-formulated liposome named lipoprodrug to safely and effectively deliver cabazitaxel, which could reduce its systemic toxicity in vivo. It could decrease tumor invasiveness and reprogram the TIM via promoting proinflammatory macrophage polarization.^1102^ Nanoparticles loaded with an antihelminthic drug, Niclosamide (Ncl) (Ncl-NPs), could prevent and reverse pulmonary fibrosis by inhibiting M2-type macrophage polarization and blocking TGF-β/Smad and STAT3 signaling pathways.^1103^

Although there is a lot of research on NDDS-based macrophage immunotherapy, the number of projects approved for clinical trials is very limited. OP-101, a nanotherapeutic compound composed of generation-4 hydroxyl-terminated poly(amidoamine) dendrimer and N-acetyl cysteine (NAC), could target activated macrophages and improve outcomes in many preclinical models of systemic inflammation and neuroinflammation.^1104–1108^ Besides, in a multicenter 2a clinical trial, researchers evaluated the safety and preliminary efficacy of OP-101 in patients with severe COVID-19(NCT04458298). The findings demonstrated that OP-101 was well tolerated in patients with severe COVID-19 and showed promising preliminary efficacy. There was an improvement in the composite clinical outcome of mechanical ventilation or death up to day 60, along with a reduction in inflammation and neurological injury markers. No drug-related adverse events (AEs) were reported in the primary endpoint^1109^ (Table 4).

**Table 4:** The summary of macrophage associated NDDS in several diseases

NDDS name	Agent	Target/Drug category	Combination therapy	Mechanism	Disease	References
pIL-12 + PLX@cR-PssPD	PLX3397(PLX)	CSF-1R inhibition	pIL-12	The promotion of the TAMs repolarization	Melanoma and colon cancer	^1079^
/	CSF-1R siRNA	CSF-1R inhibition	/	The promotion of the TAMs repolarization	Prostate cancer	^1080^
CCR2-MM@PLGA/Cur	CCR2 overexpression on the MM	trapping CCL2	/	The reduction of macrophage infiltration and microglia pro-inflammatory polarization	Spinal cord injury (SCI)	^1081^
MSN@CS/HA	Hydrogen molecules	increased CCL2 expression	/	The promotion of the anti-inflammatory M2-type polarization	Burn skin wounds	^1082^
B^BLZ-945^@PAC-PTX	BBLZ-945	CSF-1R inhibition	/	The depletion of TAMs	Melanoma and breast cancer	^1083^
	PBA-pAMD-Cholesterol (PAC)	CXCR4 blockade	/	The reduction of macrophage recruitment		
/	CXCR4 overexpression on the MSCs	The neutralization of CXCL12	/	The inhibition of mononuclear macrophages infiltration	Ischemic stroke	^1084^
	A151	cGAS inhibition	/	The promotion of the microglia M2-type polarization via inhibiting cGAS-STING pathway		
CV-S NPs	Valproic acid(VPA)	HDAC inhibitor	/	The suppression of the TLR4-MyD88-NF-κB signaling and TNF-α secretion of macrophages	Inflammation	^1085^
PLGA-ION-R837@M	Imiquimod (R837)	TLR7 agonist	Fe_3_O_4_ NPs	The promotion of the TAMs repolarization	Breast cancer	^1086^
D&R-HM-MCA	Resiquimod(R848)	TLR7/8 agonist	/	The promotion of the TAMs repolarization	Glioblastoma	^1089^
NAcp@CD47	Anti-CD47 antibodies	CD47 blockade	Cyclic di-GMP(CDG)	The promotion of macrophages/microglia phagocytosis and TAMs M1-type polarization	Glioblastoma	^1091^
/	TTA-Q6	T-type calcium channel inhibitor	/	The promotion of antigen presentation by activating macrophages	Lung cancer	^1092^
	RRX-001	CD47 inhibitor	/	The promotion of macrophages phagocytosis		
MPIRx	RRX-001	CD47 inhibitor	IR780	The promotion of phagocytosis and M1-type polarization of macrophages	Osteosarcoma	^1093^
/	RP-182	CD206 activation	/	The activation of phagocytosis and autophagy as well as promotion of the M2-type macrophage repolarization	Brainstem glioma	^1094^
			/			^1095^
siCD40/NPs	CD40 siRNA	CD40 blockade	/	The depletion of macrophages and inhibition of macrophage differentiation	Alloimmune responses in skin allotransplantation	^1096^
/	IPI-549	PI3Kγ inhibitor	Photosensitizer chlorin e6 (Ce6)	The reduction of M2-type TAMs infiltration into tumors	Colon cancer	^1097^
Nano-PI	IPI-549	PI3Kγ inhibitor	Paclitaxel (PTX)	The promotion of M2-type macrophages repolarization	Breast cancer	^1098^
HN-HFPA	L-norvaline (L-Nor)	Arg-1 inhibitor	/	The increase of M1-type macrophages ratio in tumor	Triple negative breast cancer (TNBC)	^1099^
*n*-PN/*p*-LP@aero-μGel	Imatinib	Tyrosine Kinase Inhibitors (TKI)	/	The promotion of M1-type macrophage polarization and inhibition of M2-type macrophage polarization	*Candida albicans* Infection	^1100^
GBZ-Loaded Lipo-NPs	Guanabenz(GBZ)	Alpha-2(α2) adrenergic receptor agonist	/	The reduction of TLR4-induced inflammatory cytokines secretion by macrophages	Inflammation	^1101^
lipoprodrug	Cabazitaxel	Taxanes	/	The promotion of proinflammatory macrophage polarization	Melanoma	^1102^
Ncl-NPs	Niclosamide (Ncl)	Anthelmintic	/	The inhibition of M2-type macrophage polarization	Pulmonary fibrosis	^1103^

### Potential challenges and future directions in targeting macrophages

The current mainstream classification of macrophages is based on their activation mode, molecular expression, and function, which divides them into pro-inflammatory and anti-tumor M1 type as well as pro-tumor and anti-inflammatory M2 type.^58,59^ However, this classification still has some limitations. First, the state of macrophages is dynamic, and they may vary dynamically between types M1 and M2, influenced by environmental and other factors. In addition, macrophages often present in various states and subsets in different tissues, not only characteristic M1 and M2 types. Today, although many studies are focusing on M1 and M2 markers, the specificity of these markers remains a problem, and the criteria used by different groups, as well as their studies, vary. Therefore, the diversity and plasticity of macrophages make the development and application of therapeutic methods targeting macrophage polarization face challenges.^348,761,1110,1111^ Research for identifying specific macrophage polarization markers and the correct and refined distinguishment of macrophage subpopulations are imperative for developing macrophage-targeted therapies.^1074^ In addition, activation of macrophages may induce excessive immune responses, triggering or exacerbating inflammation or several autoimmune diseases.^1112,1113^ Therefore, macrophage therapies need to be designed to strike a balance between safety and efficacy.

Although the research and development of NDDS have led to the optimization of macrophage-specific drugs and immunotherapy, their current application still poses certain challenges. First, the immunogenicity, potential toxicity, and long-term safety of many newly developed human nanomaterials have not been recognized. In addition, the production process of NDDS is complex and highly costly. Besides, the accurate release of drugs from NDDS in vivo still has some technical difficulties and needs further optimization.^1074,1114–1116^

## Conclusions and perspectives

Tissue macrophages play critical roles in maintaining tissue homeostasis, regulating immune responses, and mediating pathological processes in various diseases. The origins, heterogeneity, and functional polarization of tissue macrophages have been extensively investigated in recent decades. Primitive hematopoietic progenitors, EMPs, and HSCs have all been identified as sources for tissue macrophages, with EMPs and EMP-derived macrophage precursors (PreMacs) being the major contributors to TRMs during embryonic development. The tissue microenvironment significantly shapes the identity and function of tissue macrophages, as evidenced by the tissue-specific transcriptional profiles and phenotypic features. Further research is needed to fully elucidate these different progenitor populations’ ontogeny and relative contributions to TRMs.

Macrophages exhibit diverse biological functions, including pathogen clearance, antigen presentation, tissue repair, and iron metabolism regulation. The dysregulation of macrophage activities has been closely associated with the pathogenesis of various diseases, such as cancer, autoimmune disorders, neurodegenerative diseases, and metabolic disorders. In the TME, TAMs can promote or suppress tumor progression through complex crosstalk with other cellular components. In autoimmune disorders, macrophages release pro-inflammatory cytokines that can perpetuate inflammation and immunity. Likewise, macrophages infiltrate the adipose tissue and release pro-inflammatory cytokines, contributing to systemic insulin resistance. In neurodegenerative diseases, macrophages may interact with neurons and release neurotoxic substances that promote neuronal death. It should be noted that the precise molecular mechanisms underlying this tissue-specific programming still need to be fully understood and warrant further investigation. While we know more about how macrophages respond in their particular niches, we still need to learn how different macrophages (produced from HSCs versus EMPs) inside a tissue integrate and react to the same signals. Besides, it is still unknown how long-term this intercellular interaction impacts tissue integrity and function or if these different macrophage populations might affect the tissue niche. The specific mechanisms and factors regulating tissue macrophages’ self-renewal and long-term maintenance also require deeper exploration. Luckily, with advanced technologies, such as single-cell genomics, spatial transcriptomics, genetic manipulation, advanced imaging, high-dimensional flow cytometry and mass cytometry, and bioinformatics approaches, in combination with traditional cell and molecular biology techniques, we will gain unprecedented insights into the complex biology of tissue macrophages, their origins, interactions, and roles in maintaining tissue homeostasis and function.

Additionally, the increasing understanding of the heterogeneity and plasticity of tissue macrophages has provided promising opportunities for developing novel therapeutic strategies. Macrophages can exhibit pro-inflammatory (M1) or anti-inflammatory (M2) phenotypes in response to environmental cues. Therapies aim to shift the balance towards a desired macrophage phenotype, such as promoting M2 macrophages to enhance tissue repair or dampen excessive inflammation. Further elucidation of the molecular mechanisms governing macrophage polarization and function will help refine these therapeutic approaches and unlock the full clinical potential of macrophage-targeted interventions. Pathogenic macrophages can accumulate and contribute to disease progression in some diseases, such as cancer or fibrosis. Therapies focus on selectively depleting or reprogramming these harmful macrophage populations to restore tissue homeostasis. Strategies include using antibody-drug conjugates, liposomal drug delivery, or genetic approaches to target and eliminate specific macrophage subsets. Besides, Macrophages can be utilized as “Trojan horses” to deliver therapeutic agents to disease sites. Macrophages can internalize and transport drugs, nanoparticles, or gene therapy vectors to target tissues, facilitating localized drug delivery. This approach can improve the therapeutic index by increasing drug accumulation at the disease site and reducing off-target effects. Specially, autologous or allogeneic macrophages can be engineered or polarized ex vivo and then administered to patients. These macrophage-based cell therapies can supplement or replace dysfunctional macrophages, modulate the immune response, or promote tissue regeneration. Approaches include ex vivo polarization of macrophages to enhance their regenerative properties before reintroduction into the body. This has implications for treating conditions like myocardial infarction and chronic wounds, where promoting tissue repair is crucial. Challenges include the scalability of macrophage production and ensuring the long-term survival and functionality of the transferred cells. Specific signaling pathways that regulate macrophage function, such as those involved in inflammation, phagocytosis, or metabolism, can be targeted by small-molecule inhibitors or biologics. This approach aims to fine-tune macrophage behavior without depleting the entire macrophage population. Identifying the appropriate therapeutic targets and balancing macrophage modulation’s beneficial and deleterious effects remain key challenges. Lastly, a new field of study is the interaction between macrophages and the microbiota. According to recent research, the gut microbiota might affect the polarization and activity of macrophages, affecting immunological responses and systemic inflammation. Gaining insight into these relationships may help develop new treatment approaches that use the microbiota to control macrophage activity in conditions like metabolic disorders and inflammatory bowel disease. Some problems need to be solved: 1. How to better regulate the TME and realize the transformation of macrophages from tumor-promoting to inhibitory? 2. How to design a more effective macrophage-based drug delivery system? 3. How to enhance the cytotoxic and anti-tumor functions of CAR-M cells? 4. How to develop more effective strategies for eliminating or depleting specific harmful macrophage subsets? 5. More accurate macrophage typing and prediction models are needed to guide individualized therapy. 6. Ensuring macrophage-based therapies are safe and do not cause off-target effects. 7. Ongoing research is needed to understand and counteract resistance mechanisms involved in macrophage-targeted therapies. 8. Identifying which specific strains or metabolites of the microbiome are beneficial or harmful to macrophage function.

In conclusion, tissue macrophages are indispensable in maintaining homeostasis and mediating pathological processes. Continued research on the origins, heterogeneity, and functional regulation of tissue macrophages will advance our understanding of their roles in health and disease and guide the development of innovative macrophage-targeted therapies.
